# Supplementary material for: Modelling EWS::FLI1 protein fluctuations reveal determinants of tumor plasticity in Ewing sarcoma
Source: EMBO Mol Med. 2026 Jan 3;18(2):646–76. doi: 10.1038/s44321-025-00364-7 (PMC12905378; doi:10.1038/s44321-025-00364-7)
Supplement: Supplementary file 1 — Appendix [file 44321_2025_364_MOESM1_ESM.pdf]

Modelling EWS::FLI1 protein fluctuations reveal determinants of tumor plasticity in Ewing sarcoma

Veveeyan Suresh[1]*, Christoph Hafemeister[1]*, Andri Konstantinou[2,3], Sarah Grissenberger[1], Caterina Sturtzel[1], Martha M Zylka[1], Florencia Cidre-Aranaz[4], Andrea Wenninger-Weinzierl[1], Karla Queiroz[5], Dorota Kurek[5], Martin Distel[1,6], Anna Obenauf[2], Thomas G.P Grünewald[4], Florian Halbritter[1], Heinrich Kovar[1,7#], Valerie Fock[1#]

*equal contribution

Jointly supervised the work and corresponding authors

Table of Contents

Appendix Figure S1. 2

Appendix Figure S2 3

A

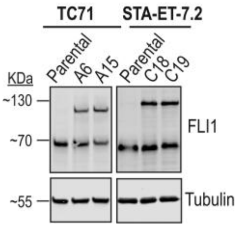

Appendix Figure S1 – C-terminal EF tagging leads to EF gene duplication in TC71 and STA-ET-7.2 cells.
(A) Western blot analysis showing protein levels of endogenous untagged EF (~70 kDa) and dTAG-fused (~130 kDa) EF in parental (TC71, STA-ET-7.2) and EF-dTAG clones (TC71-A6, A15; STA-ET-7.2-C18, C19). Tubulin was used as a loading control. Representative images from one of the biological replicates are shown.

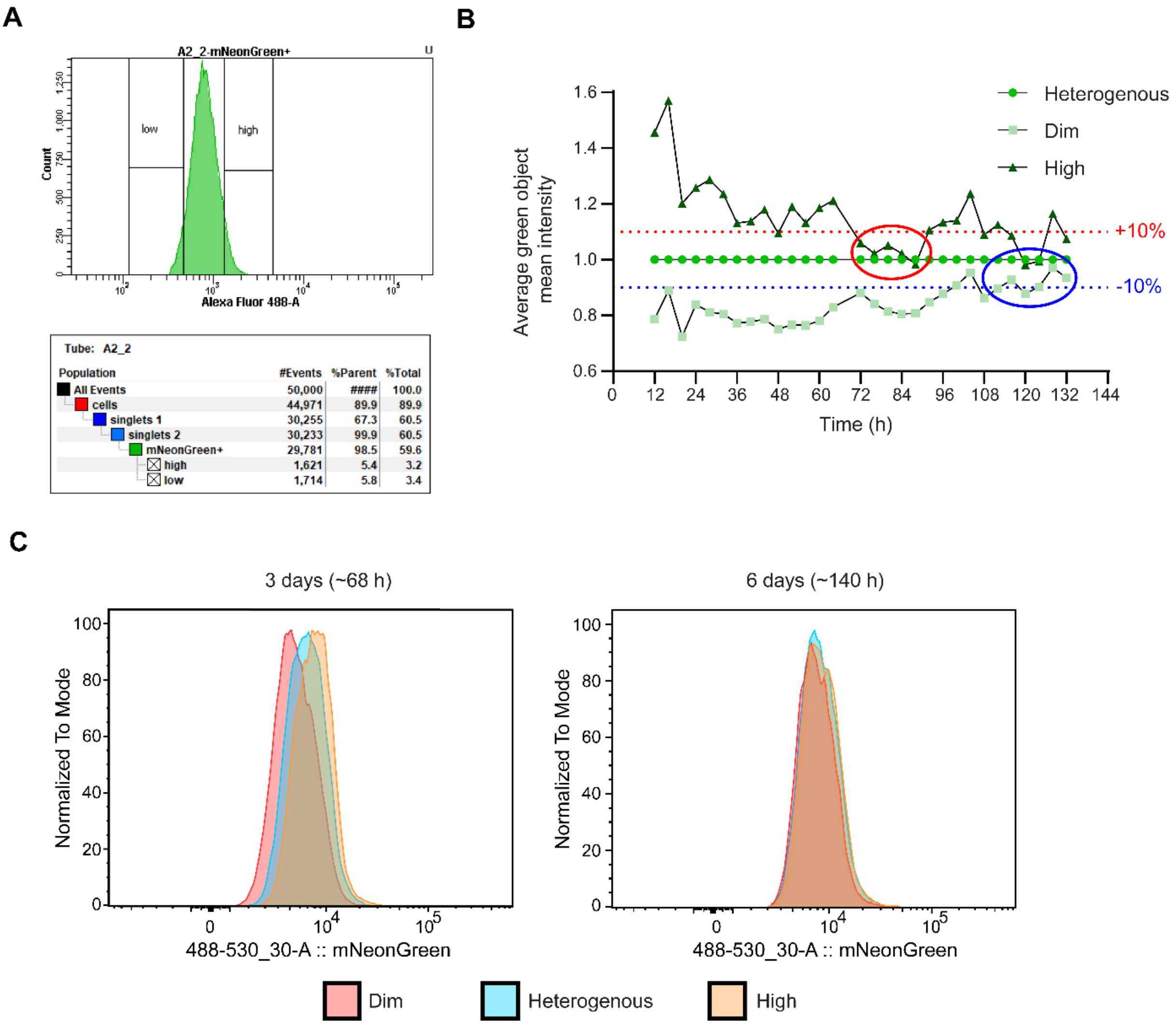

Appendix Figure S2 - Dynamic nature of EF expression in A2.2 dTAG cells.

(A) Sorting strategy of A2.2 EF-dTAG clone into subpopulations with low (mNG^{Dim}) and high (mNG^{High}) EF-mNG fluorescence intensity (n =1).

(B) Quantification of average green object mean intensity (Incucyte S3-C2) of sorted subpopulations normalized to the heterogeneous unsorted A2.2 population, starting 12 h after sorting. Dashed lines denote the equilibrium threshold (±10%), used to define the timepoint at which sorted populations converge with the heterogeneous state and remain within this range for at least three consecutive measurements. Red circle indicates the equilibrium timepoint of EF-mNG^{High} cells, blue circle indicates that of EF-mNG^{Dim} cells (n =1).

(C) Flow cytometry analysis of mNG fluorescence intensity in the sorted and heterogeneous populations at day 3 and day 6 (n =1).
