# Supplementary figures and images for: Modelling EWS::FLI1 protein fluctuations reveal determinants of tumor plasticity in Ewing sarcoma
Source: EMBO Mol Med. 2026 Jan 3;18(2):646–76. doi: 10.1038/s44321-025-00364-7 (PMC12905378; doi:10.1038/s44321-025-00364-7)

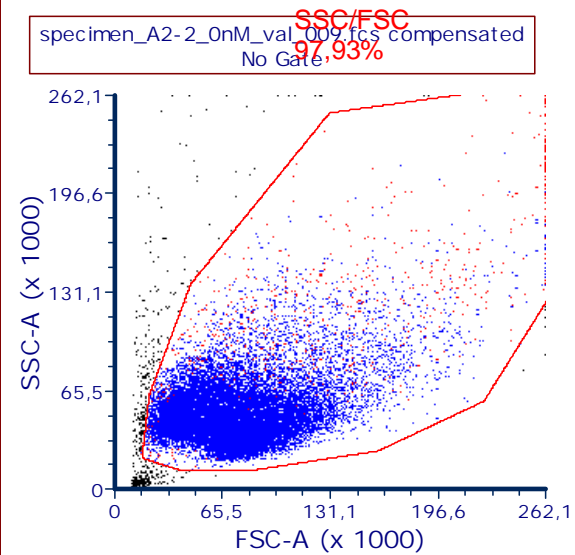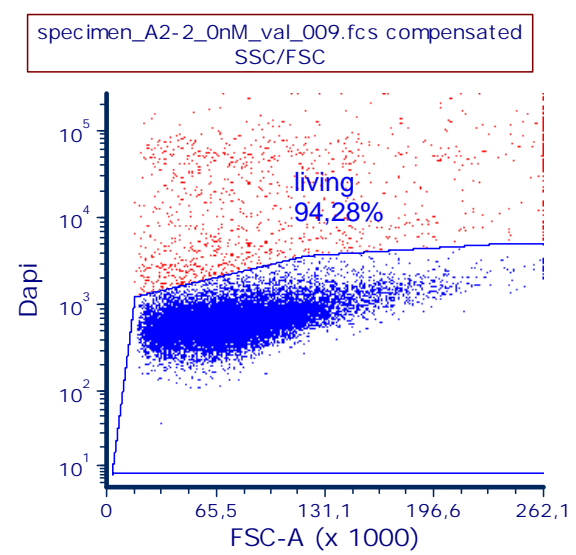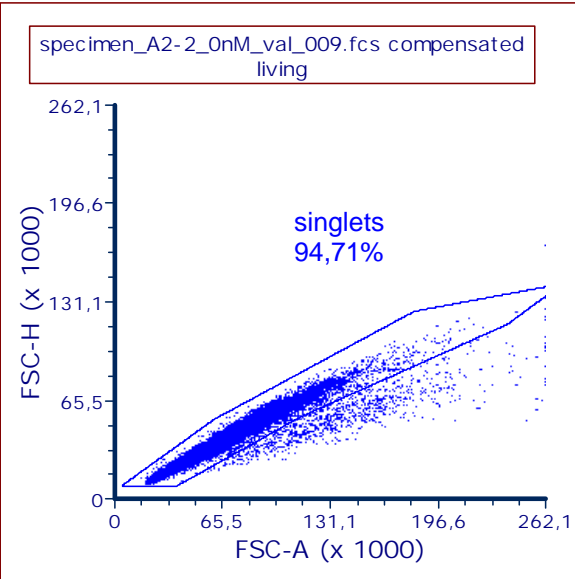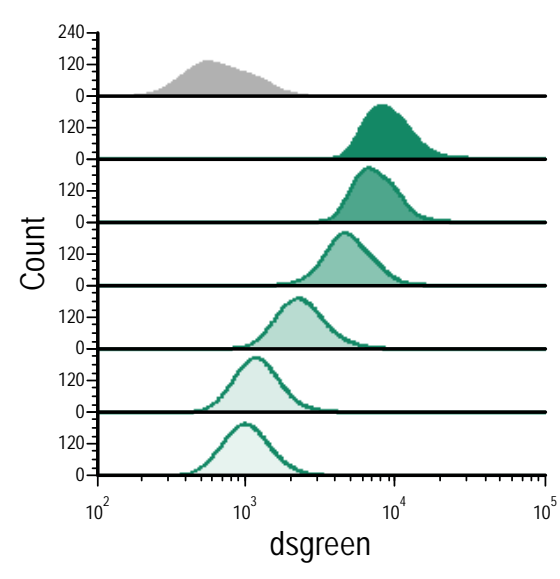

Supplement: Supplementary file 12 — Source data Fig. 1 [file 44321_2025_364_MOESM12_ESM.zip › 1C/A673 A2.2 dTAG treatment 24 hours gating.pdf]

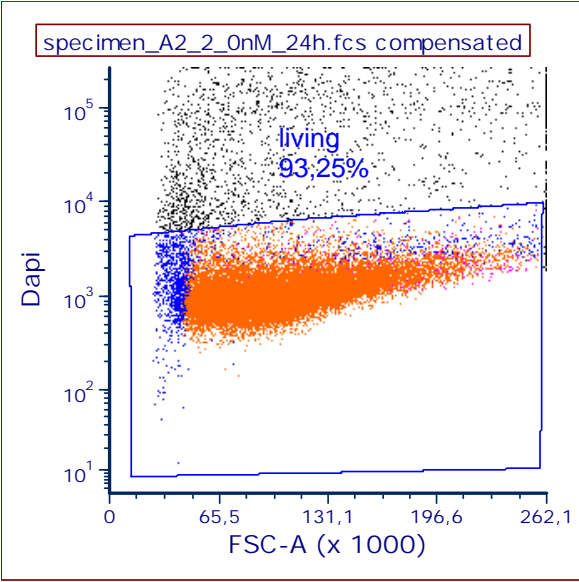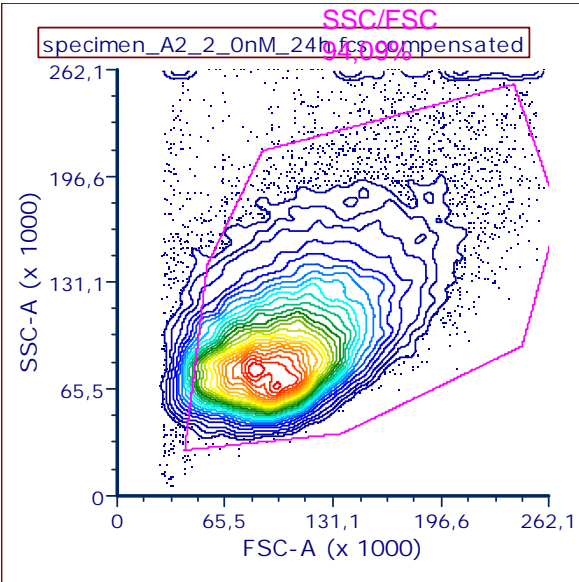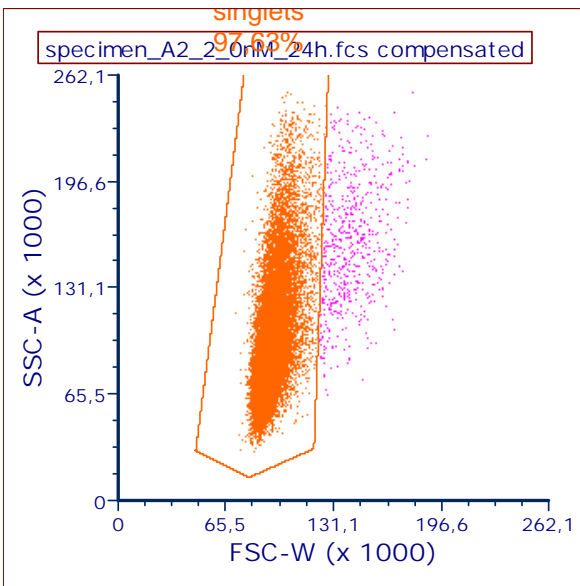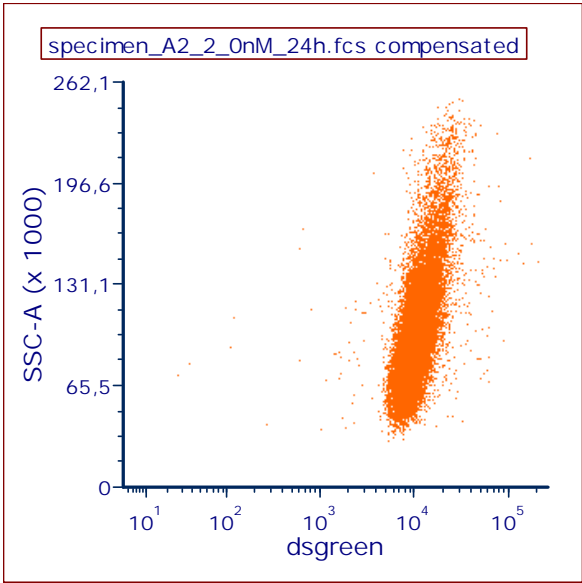

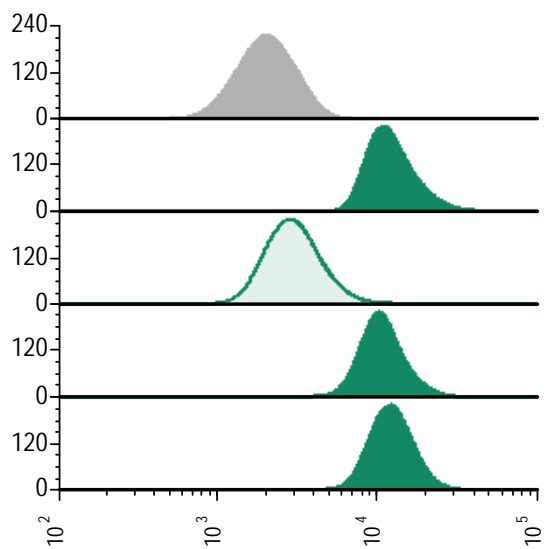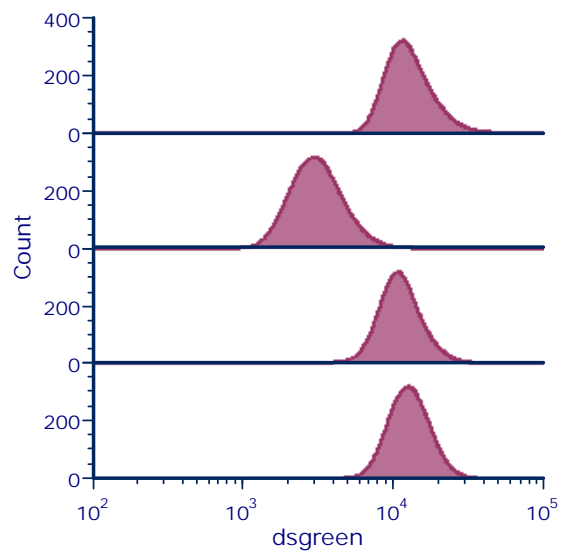

Supplement: Supplementary file 12 — Source data Fig. 1 [file 44321_2025_364_MOESM12_ESM.zip › 1D/A2.2 24 hour dTAGv-1 treatment and washout Gating.pdf]

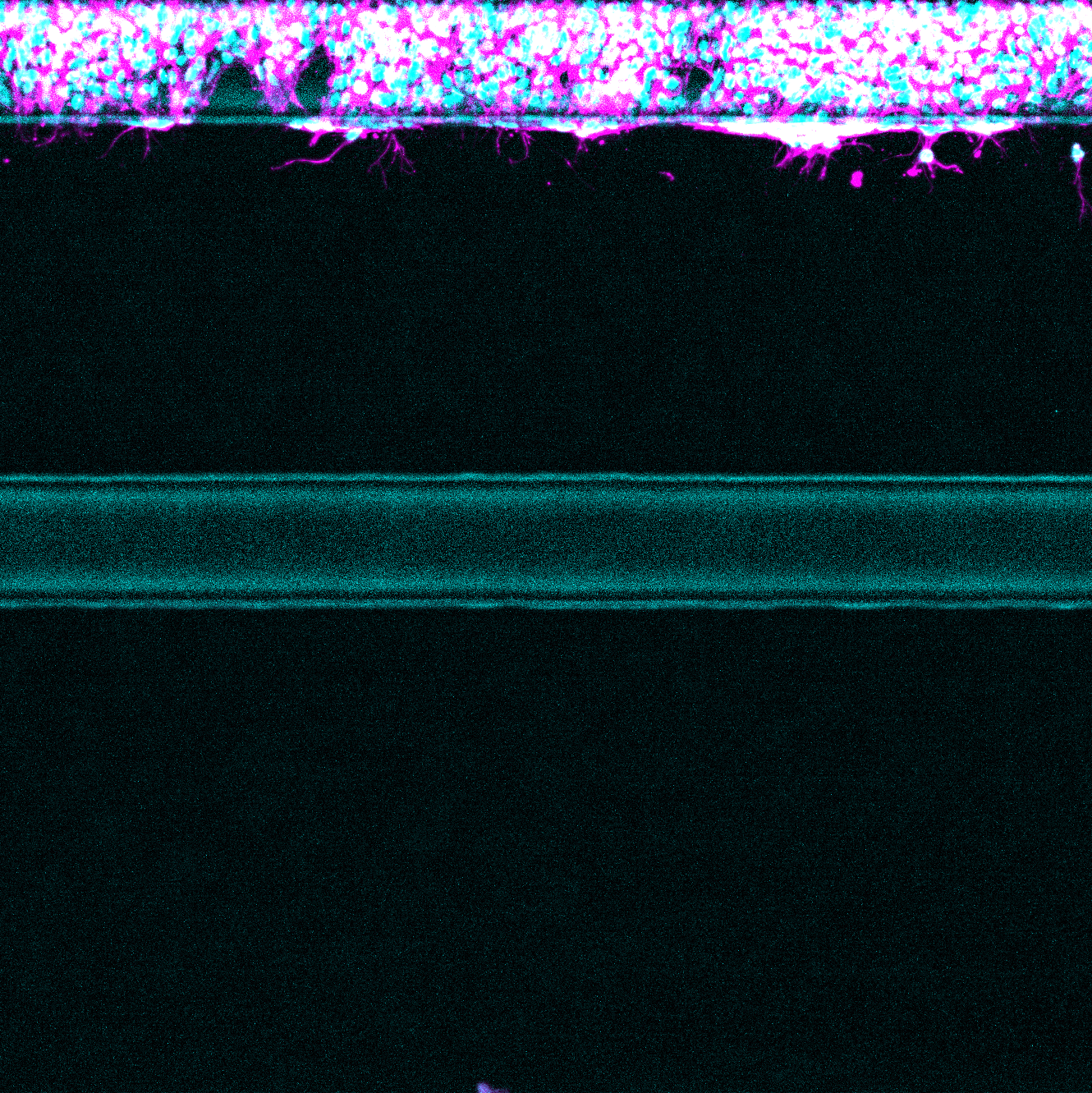

Supplement: Supplementary file 13 — Source data Fig. 2 [file 44321_2025_364_MOESM13_ESM.zip › 2B/A2.2_0nM.tif]

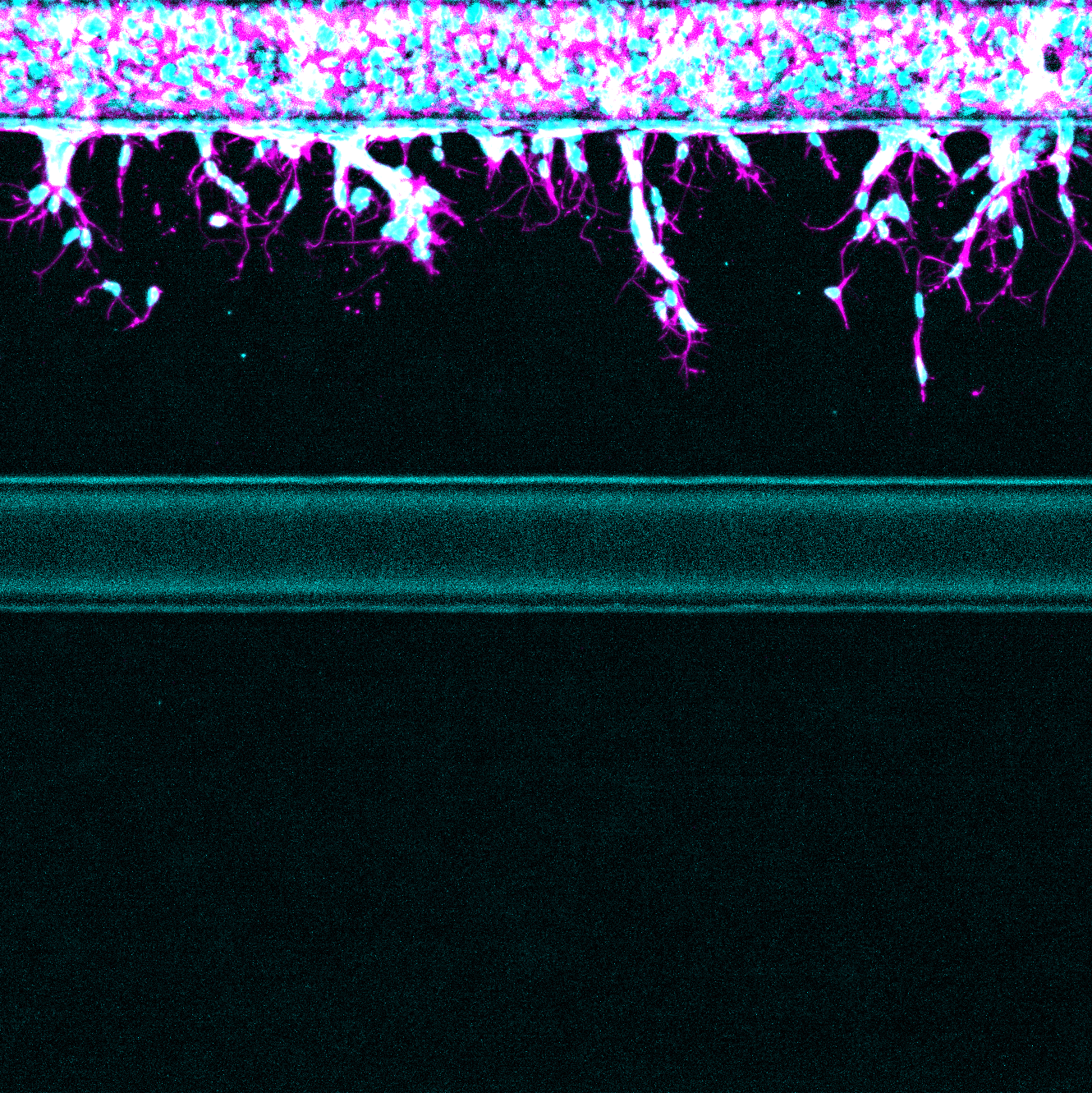

Supplement: Supplementary file 13 — Source data Fig. 2 [file 44321_2025_364_MOESM13_ESM.zip › 2B/A2.2_1.5nM.tif]

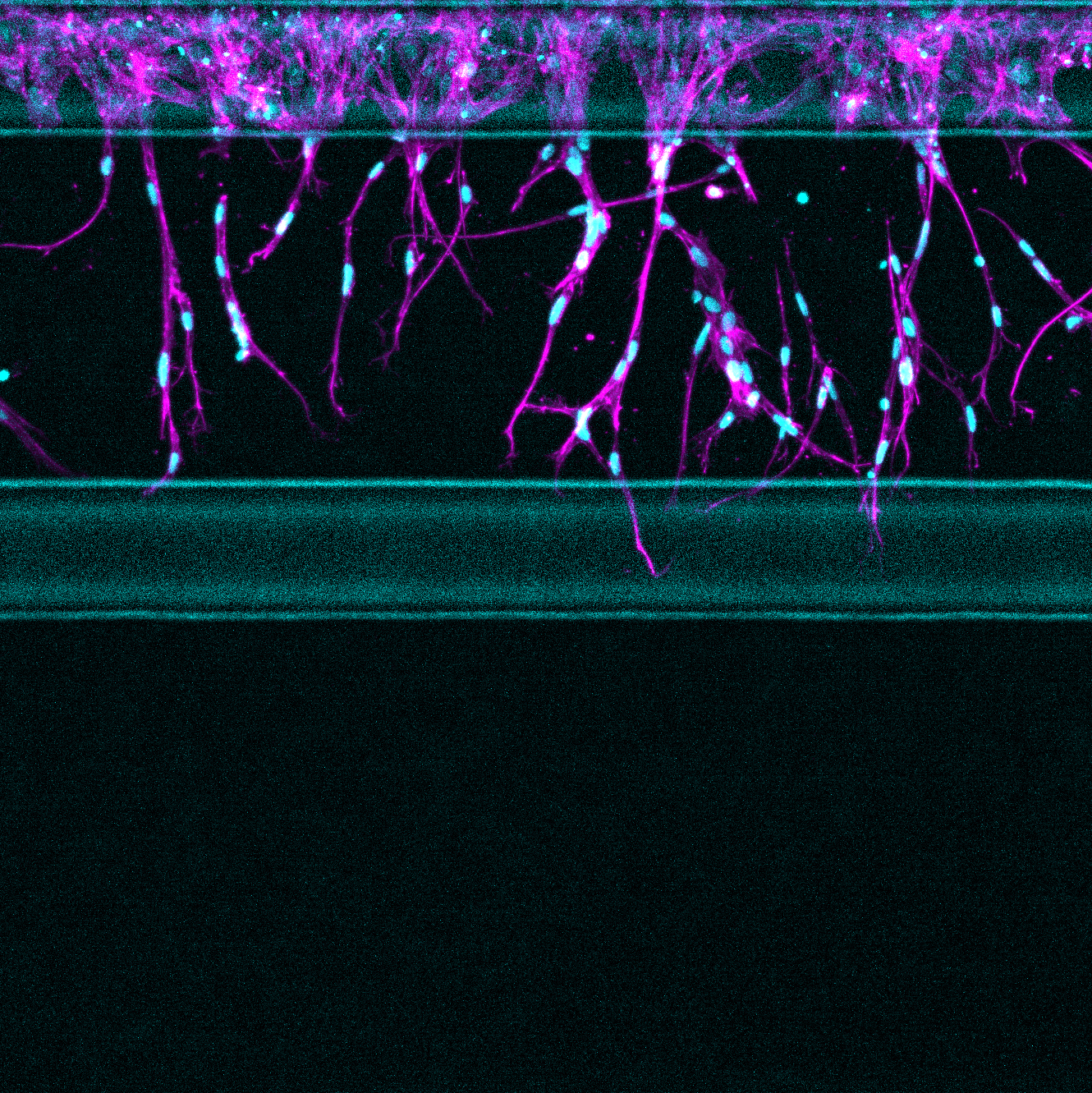

Supplement: Supplementary file 13 — Source data Fig. 2 [file 44321_2025_364_MOESM13_ESM.zip › 2B/A2.2_150nM.tif]

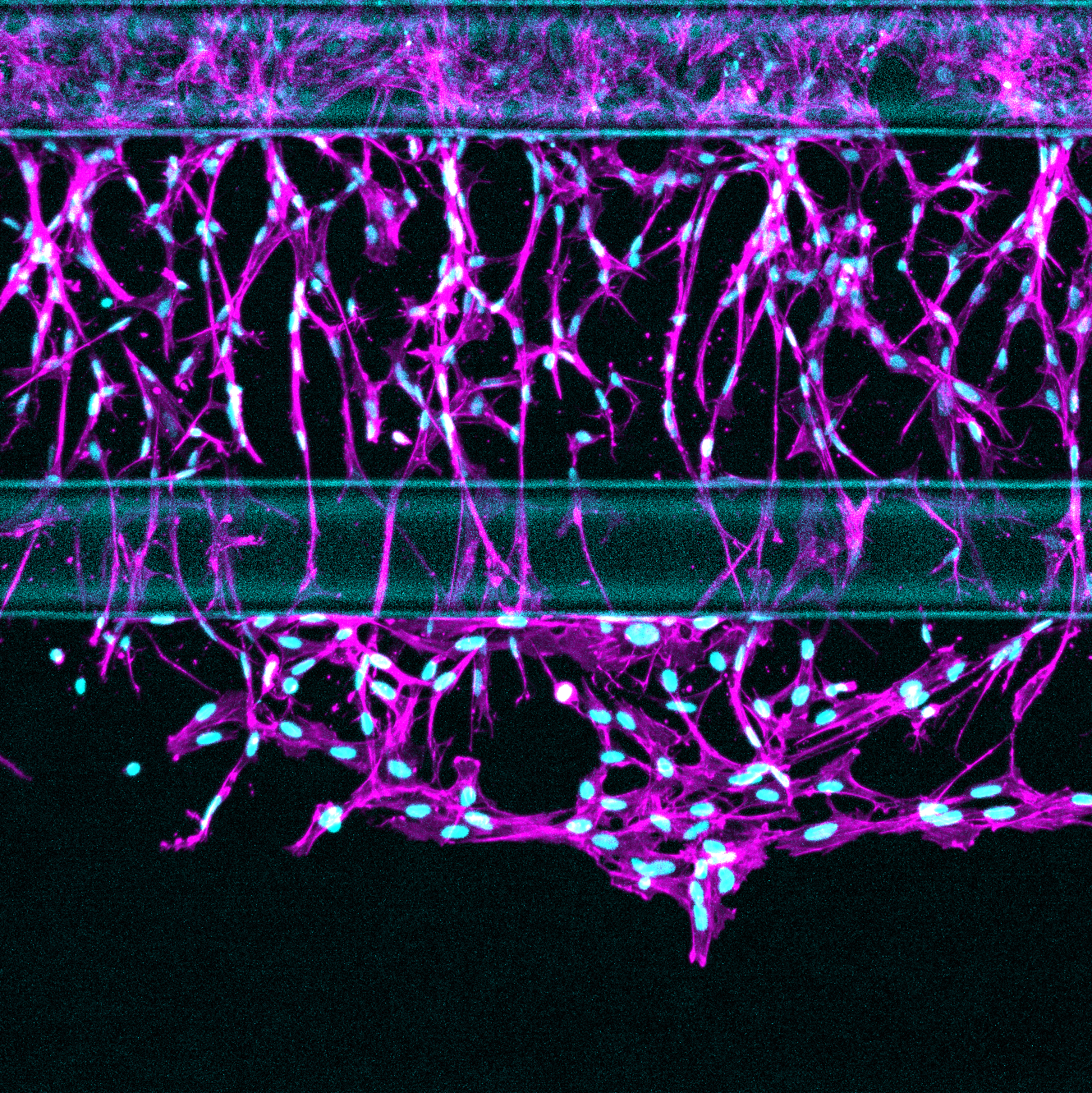

Supplement: Supplementary file 13 — Source data Fig. 2 [file 44321_2025_364_MOESM13_ESM.zip › 2B/A2.2_15nM.tif]

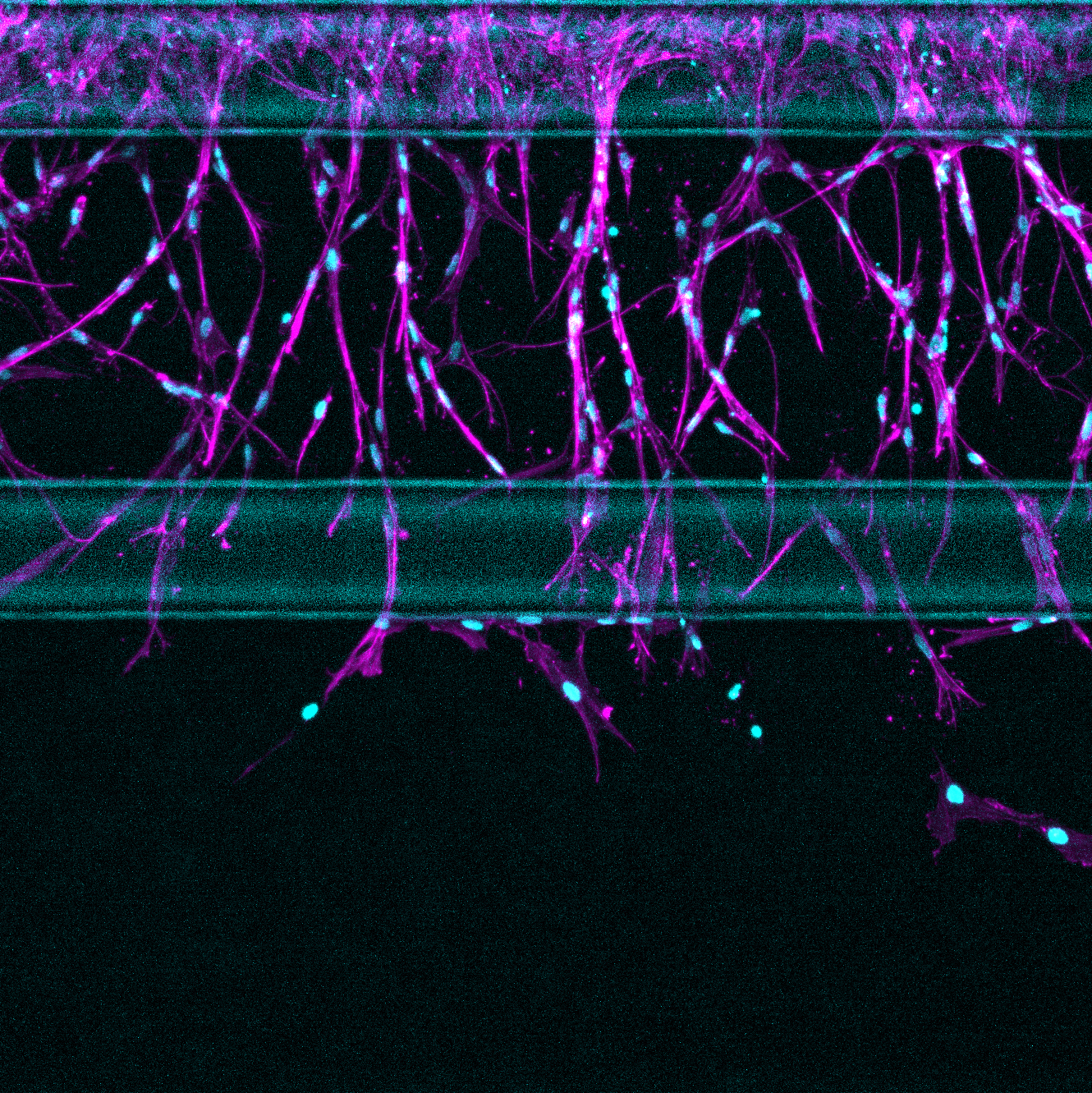

Supplement: Supplementary file 13 — Source data Fig. 2 [file 44321_2025_364_MOESM13_ESM.zip › 2B/A2.2_50nM.tif]

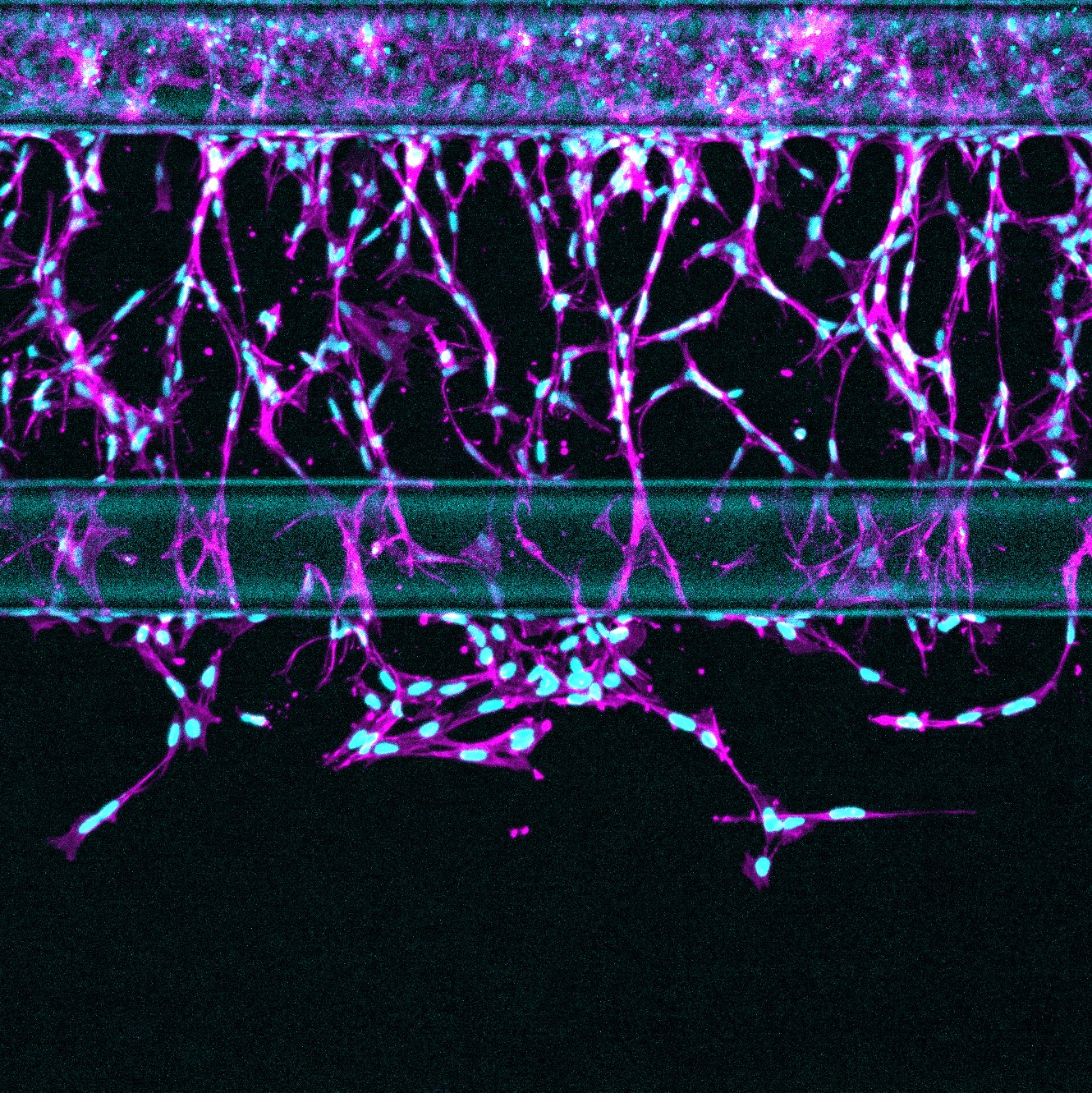

Supplement: Supplementary file 13 — Source data Fig. 2 [file 44321_2025_364_MOESM13_ESM.zip › 2B/A2.2_5nM.tif]

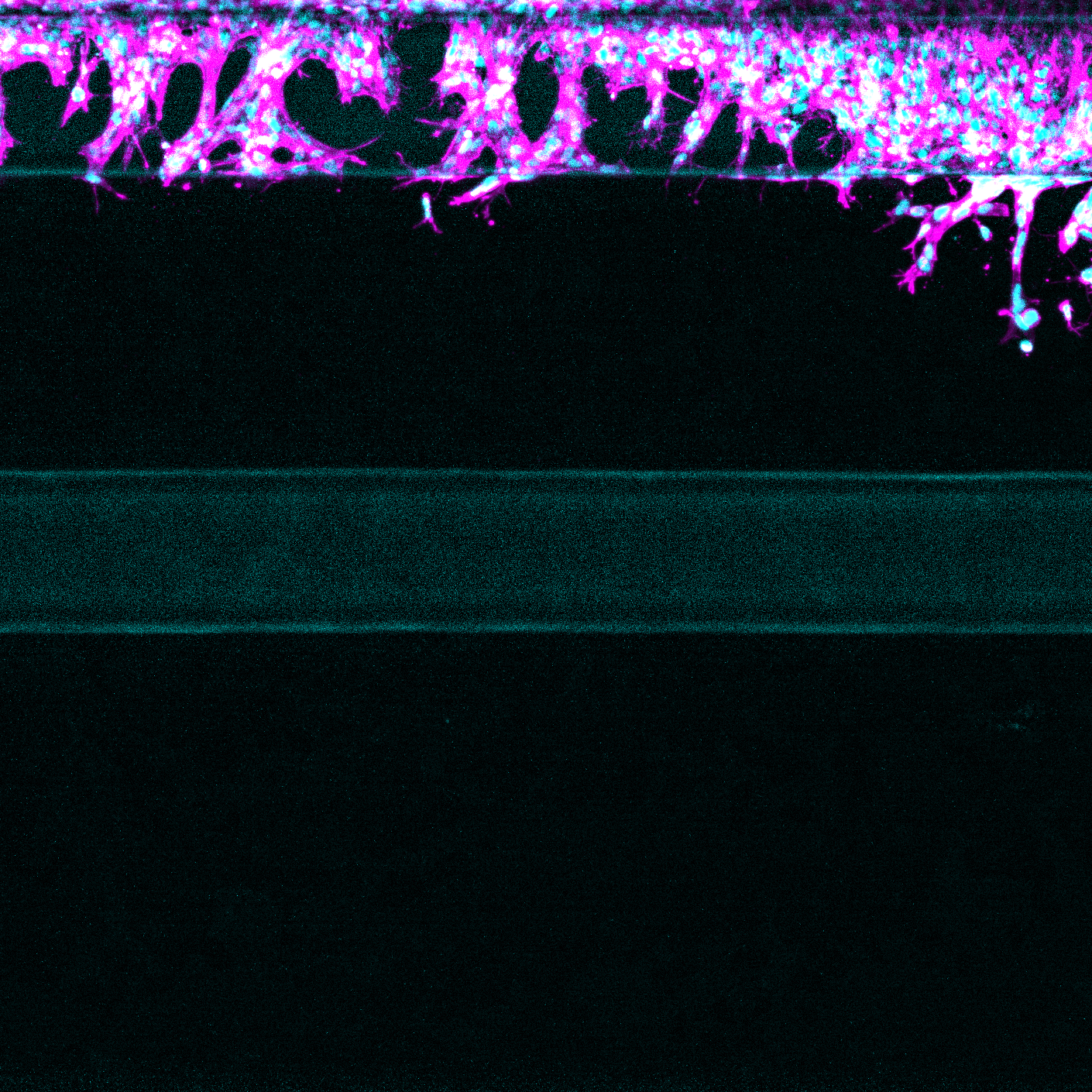

Supplement: Supplementary file 13 — Source data Fig. 2 [file 44321_2025_364_MOESM13_ESM.zip › 2B/B3.1_0nM.tif]

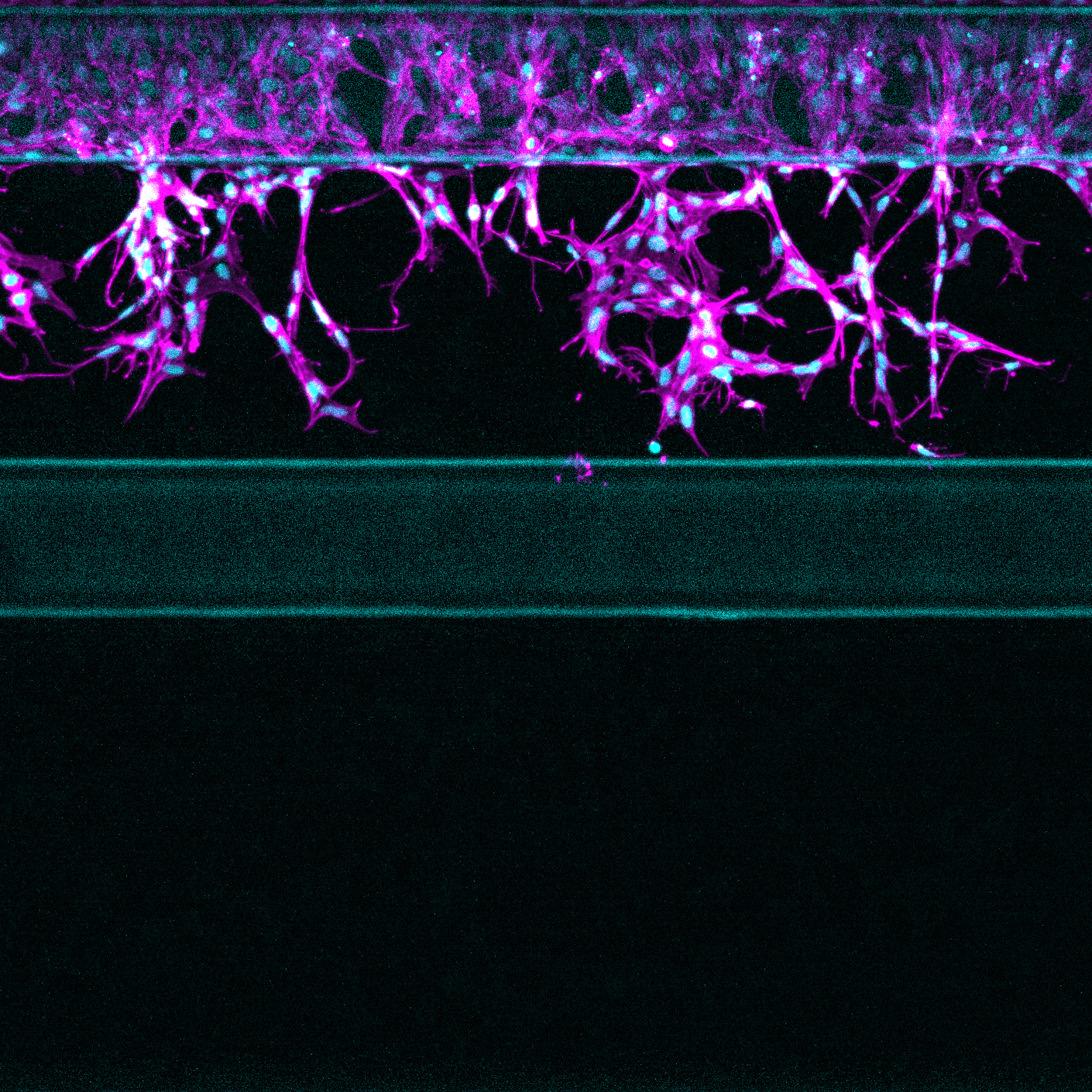

Supplement: Supplementary file 13 — Source data Fig. 2 [file 44321_2025_364_MOESM13_ESM.zip › 2B/B3.1_1.5nM.tif]

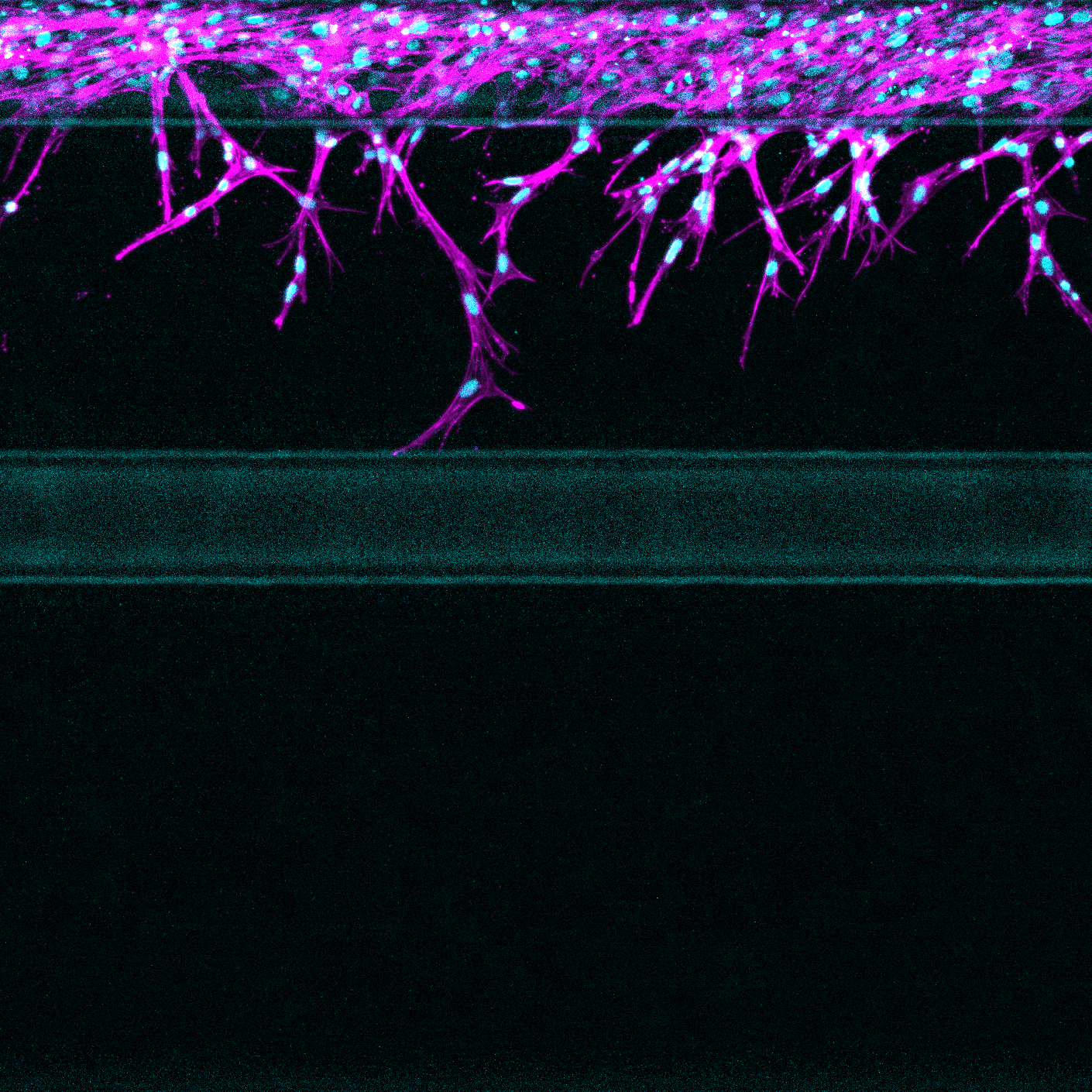

Supplement: Supplementary file 13 — Source data Fig. 2 [file 44321_2025_364_MOESM13_ESM.zip › 2B/B3.1_150nM.tif]

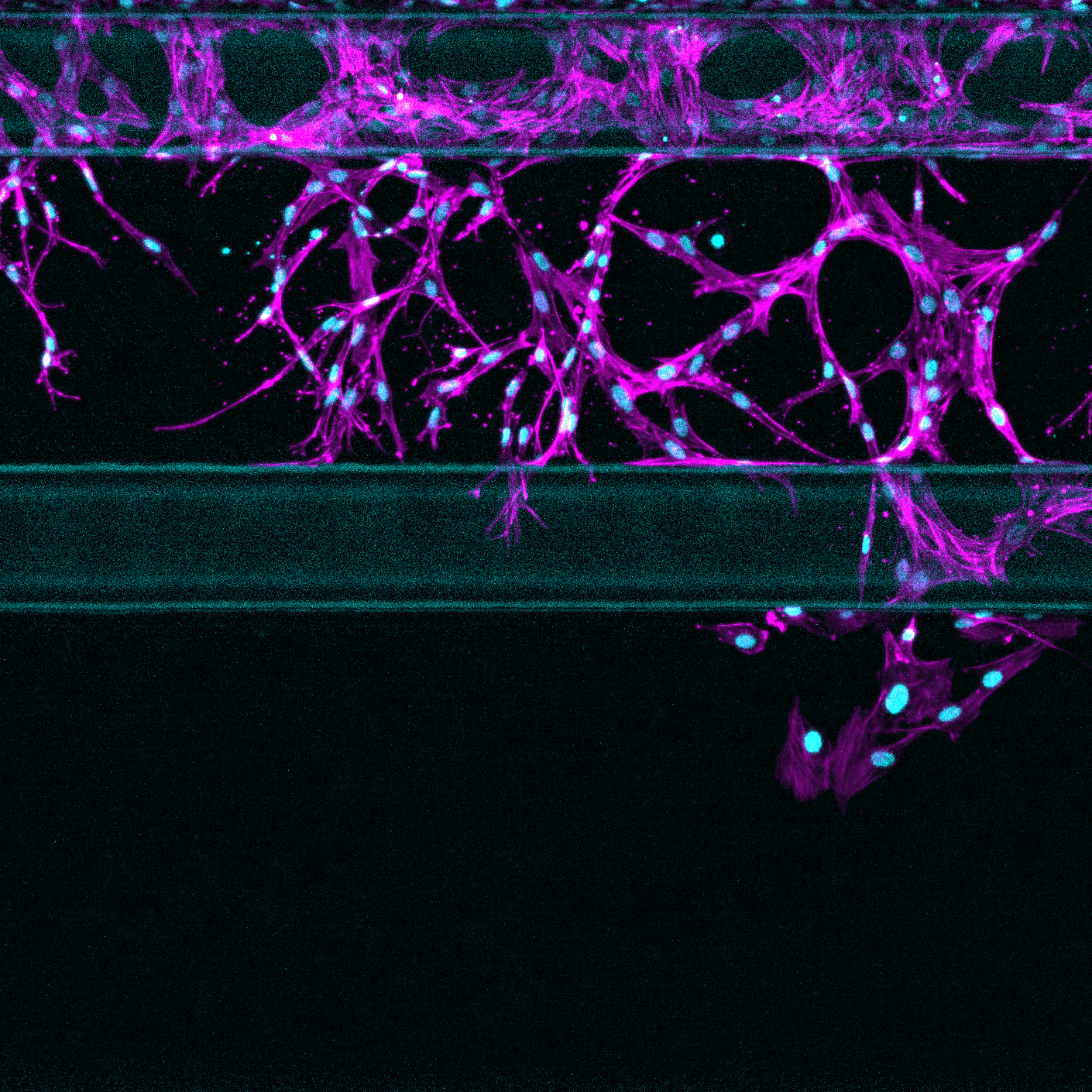

Supplement: Supplementary file 13 — Source data Fig. 2 [file 44321_2025_364_MOESM13_ESM.zip › 2B/B3.1_15nM.tif]

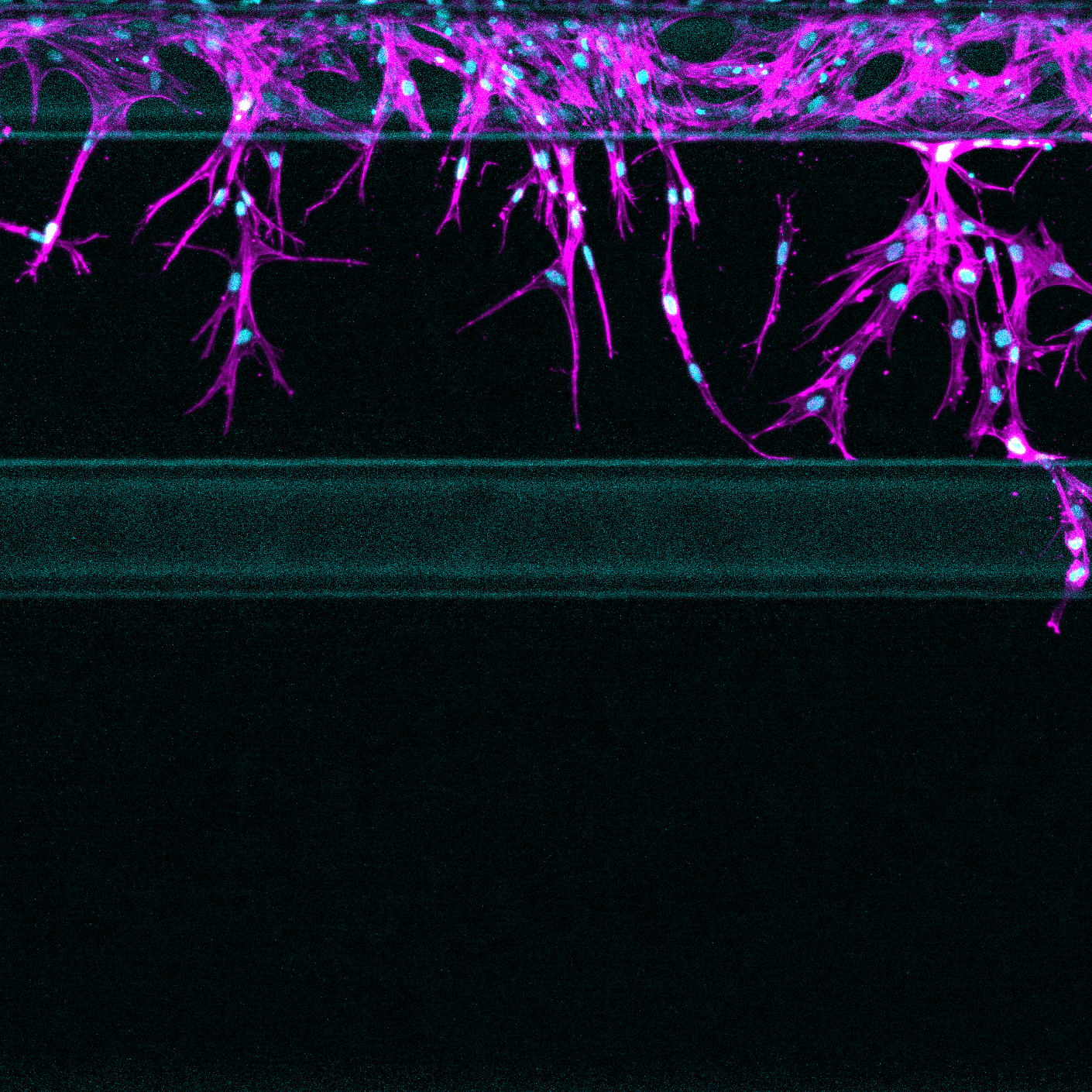

Supplement: Supplementary file 13 — Source data Fig. 2 [file 44321_2025_364_MOESM13_ESM.zip › 2B/B3.1_50nM .tif]

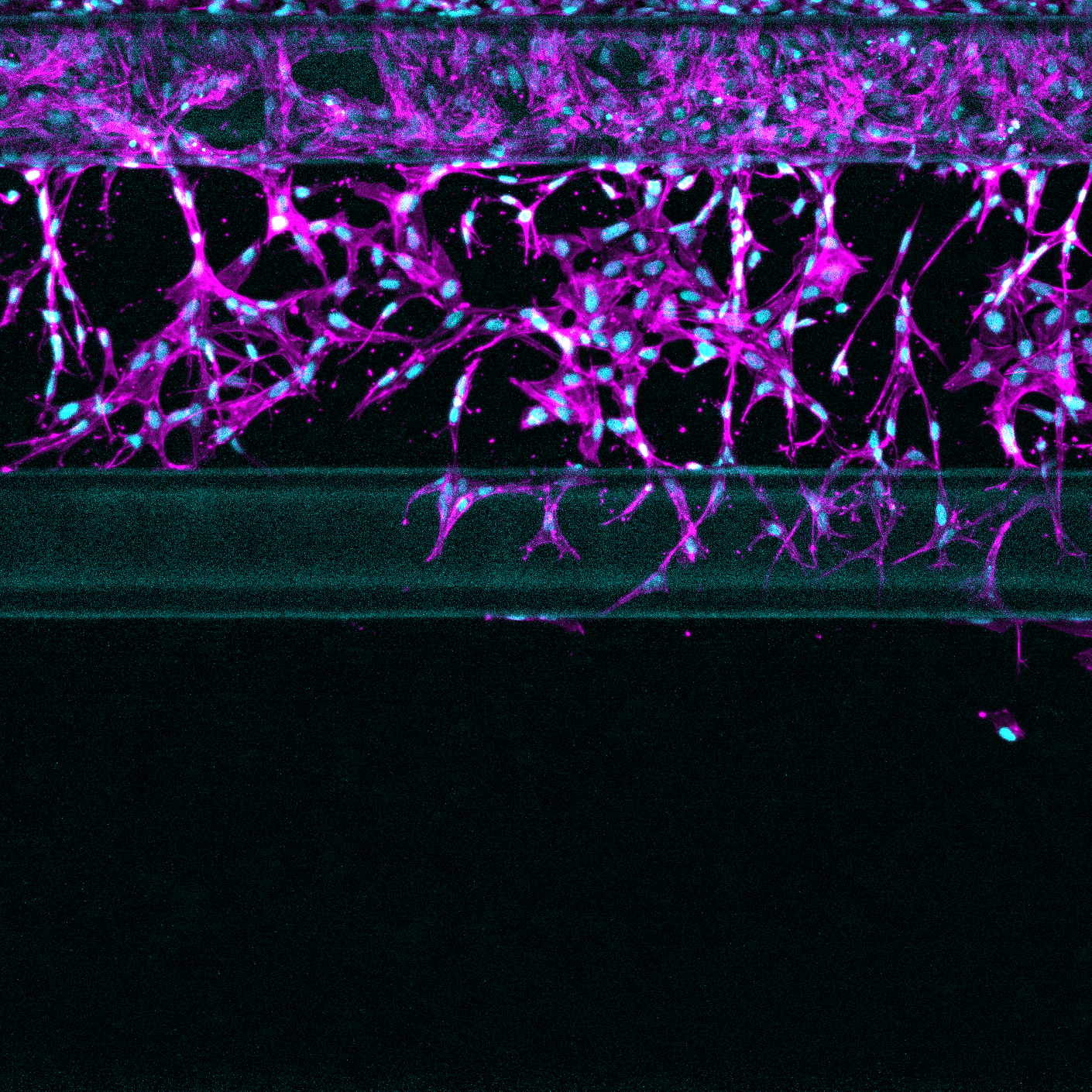

Supplement: Supplementary file 13 — Source data Fig. 2 [file 44321_2025_364_MOESM13_ESM.zip › 2B/B3.1_5nM.tif]

Uncropped 2C

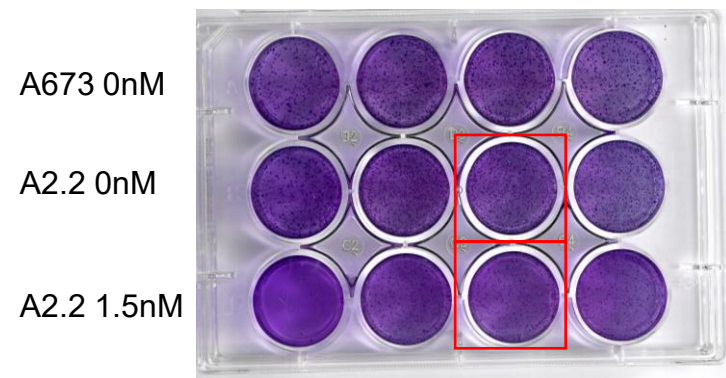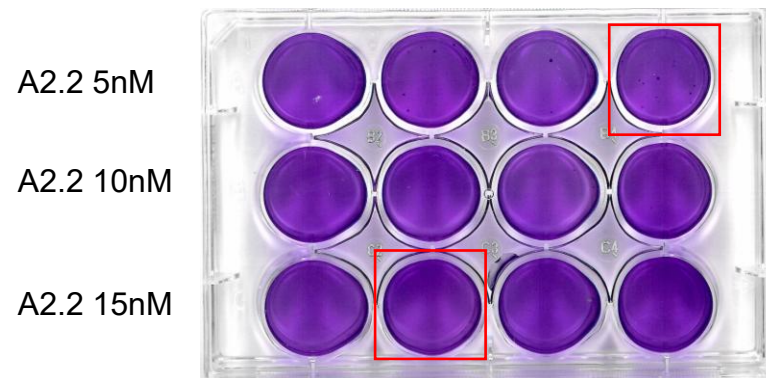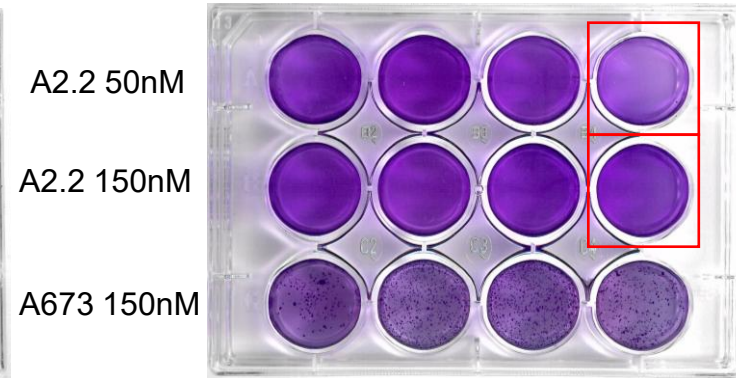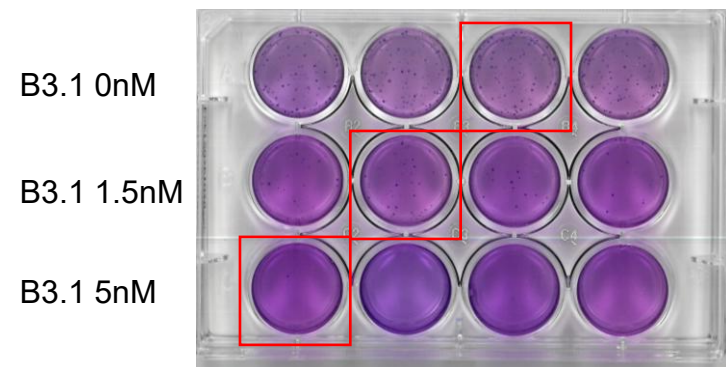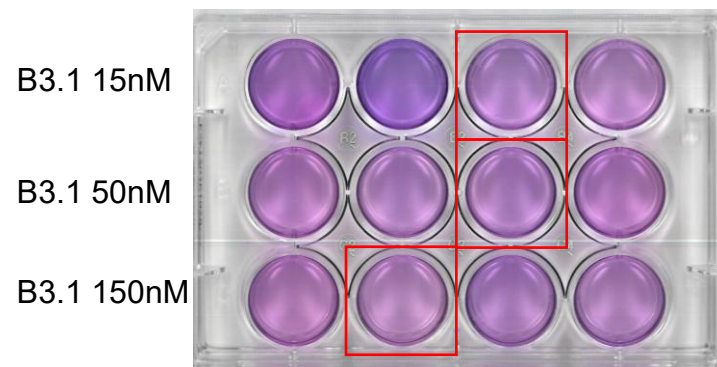

Supplement: Supplementary file 13 — Source data Fig. 2 [file 44321_2025_364_MOESM13_ESM.zip › 2C/Softagar_sourcedata.pdf]

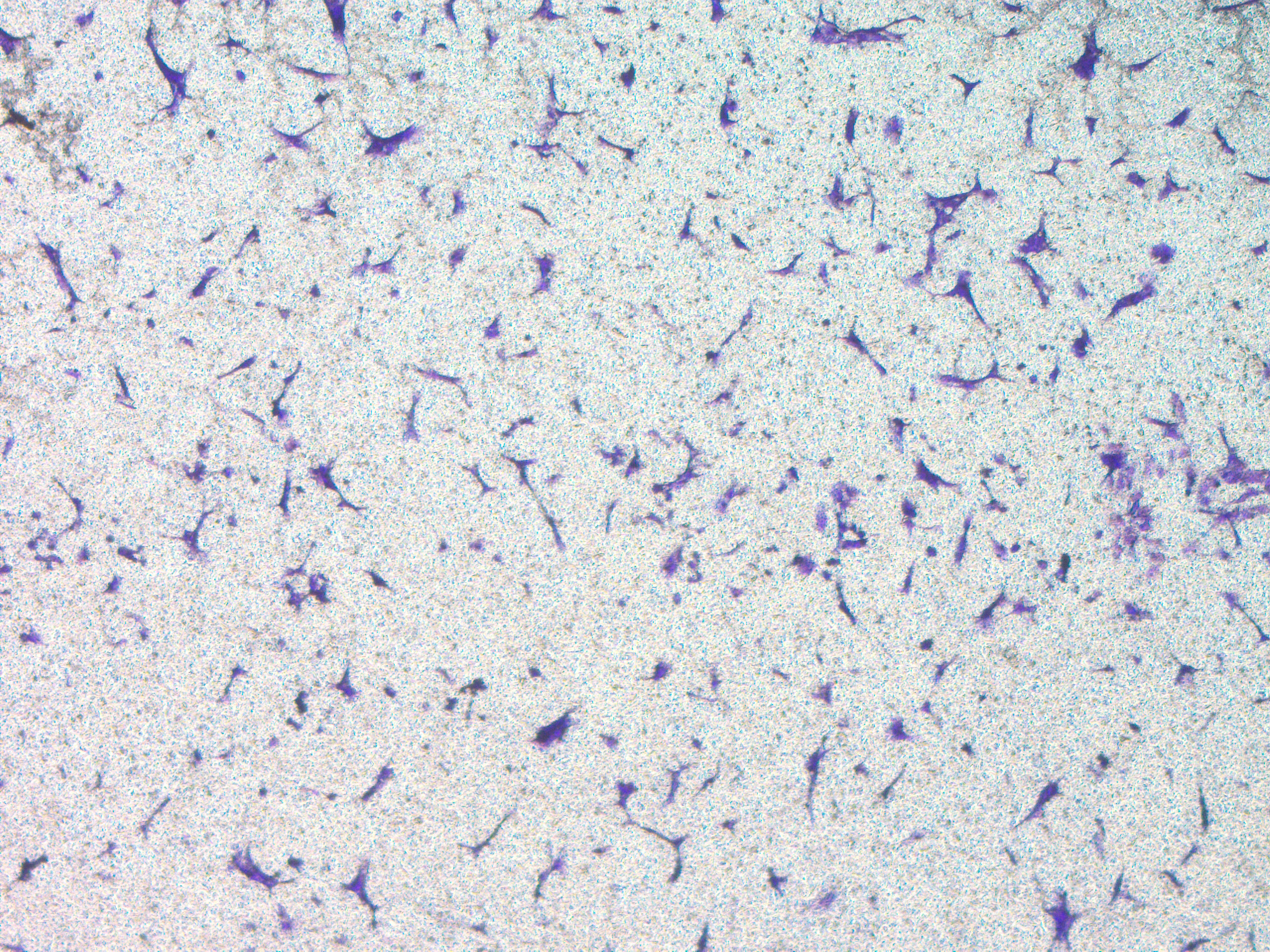

Supplement: Supplementary file 13 — Source data Fig. 2 [file 44321_2025_364_MOESM13_ESM.zip › 2A/A2.2_0nM.tif]

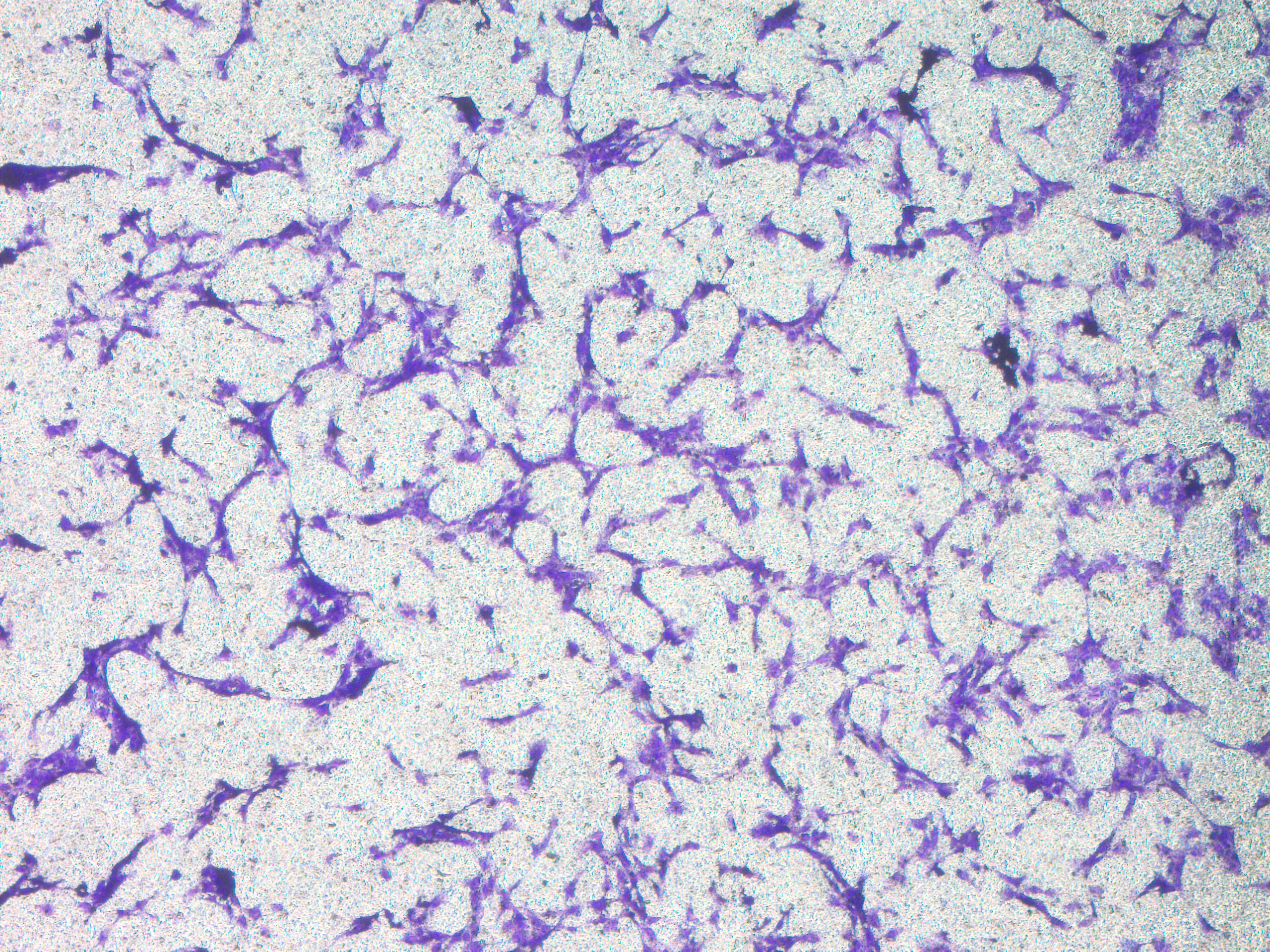

Supplement: Supplementary file 13 — Source data Fig. 2 [file 44321_2025_364_MOESM13_ESM.zip › 2A/A2.2_1.5nM.tif]

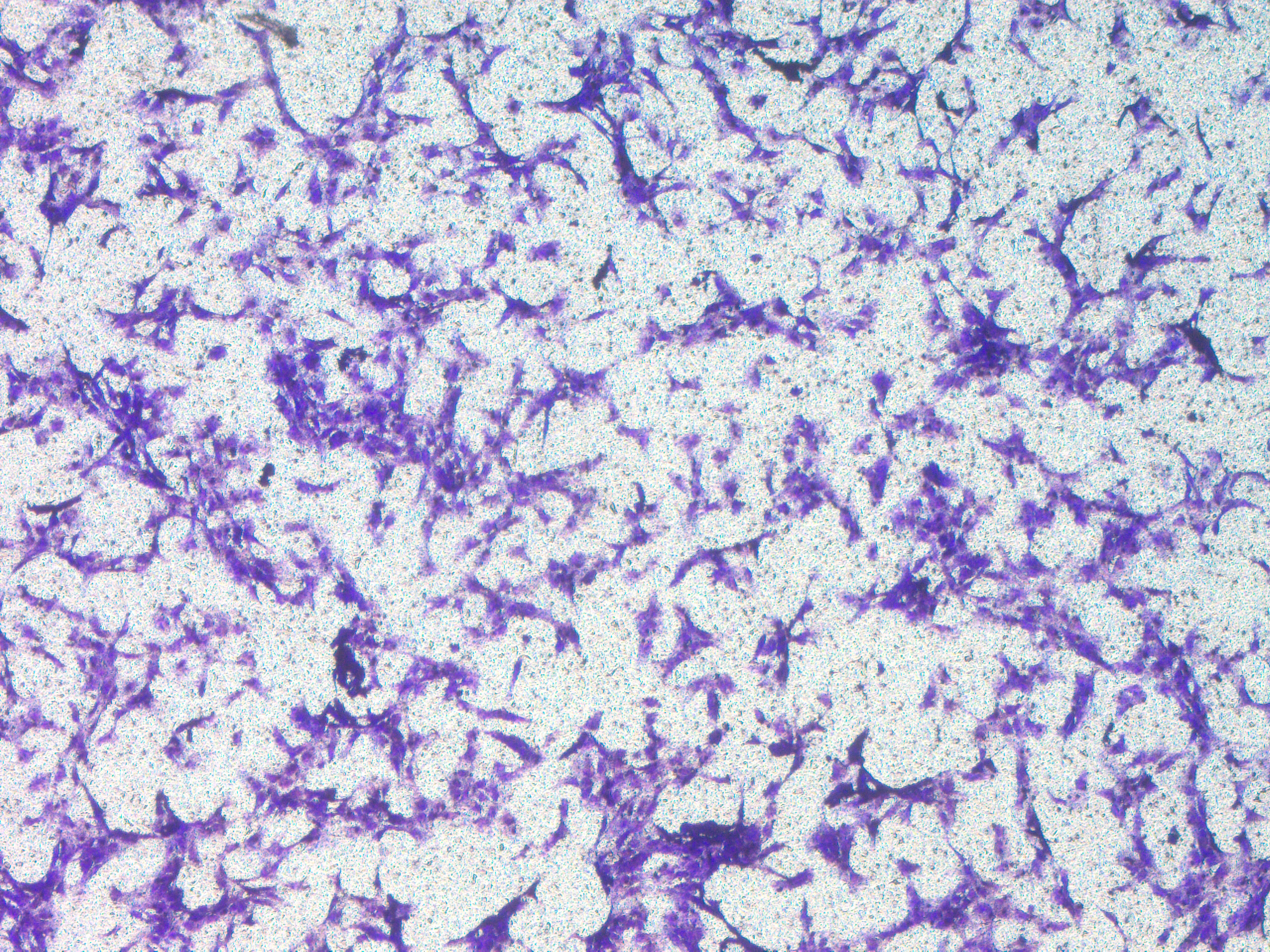

Supplement: Supplementary file 13 — Source data Fig. 2 [file 44321_2025_364_MOESM13_ESM.zip › 2A/A2.2_150nM.tif]

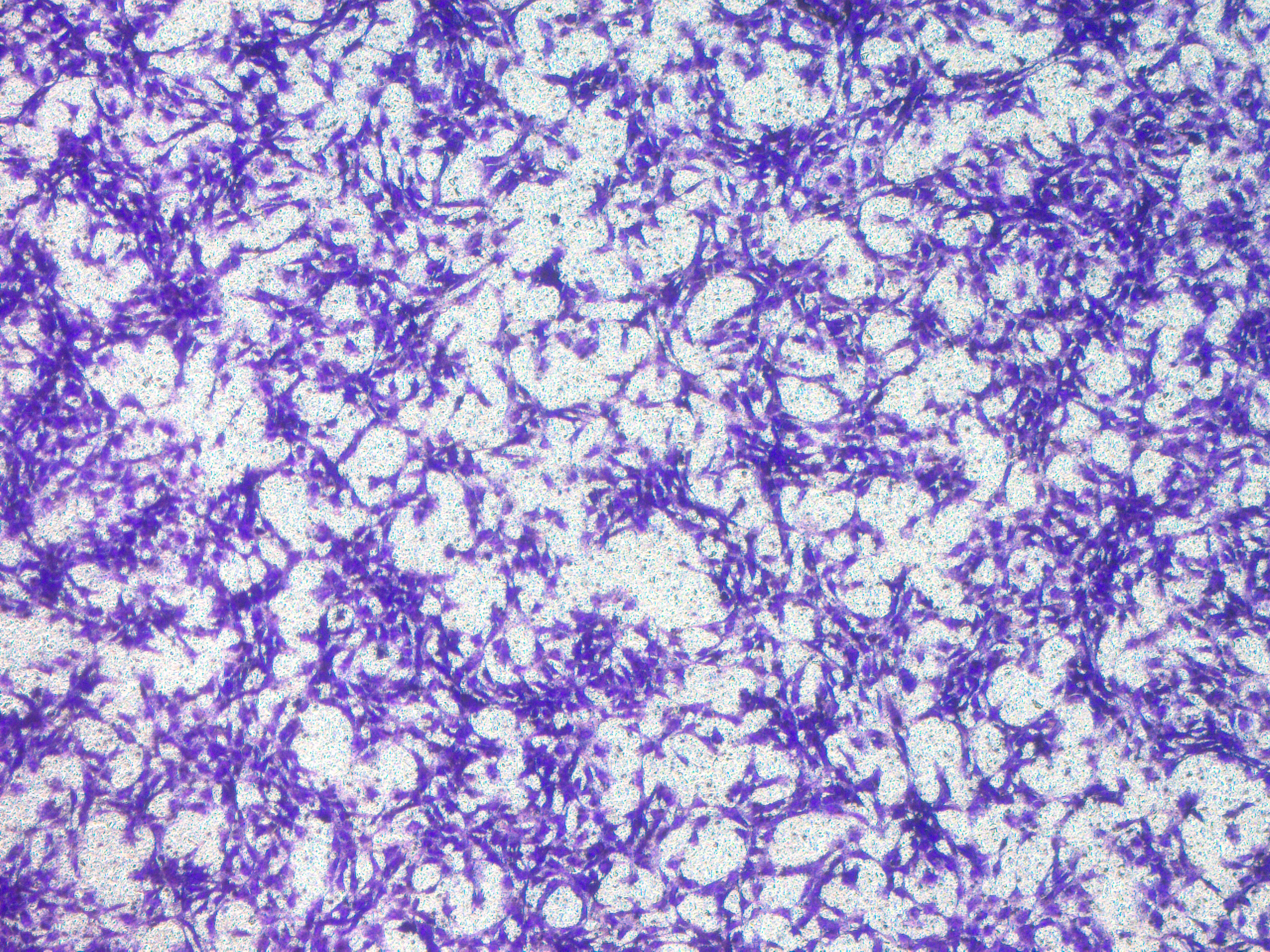

Supplement: Supplementary file 13 — Source data Fig. 2 [file 44321_2025_364_MOESM13_ESM.zip › 2A/A2.2_15nM.tif]

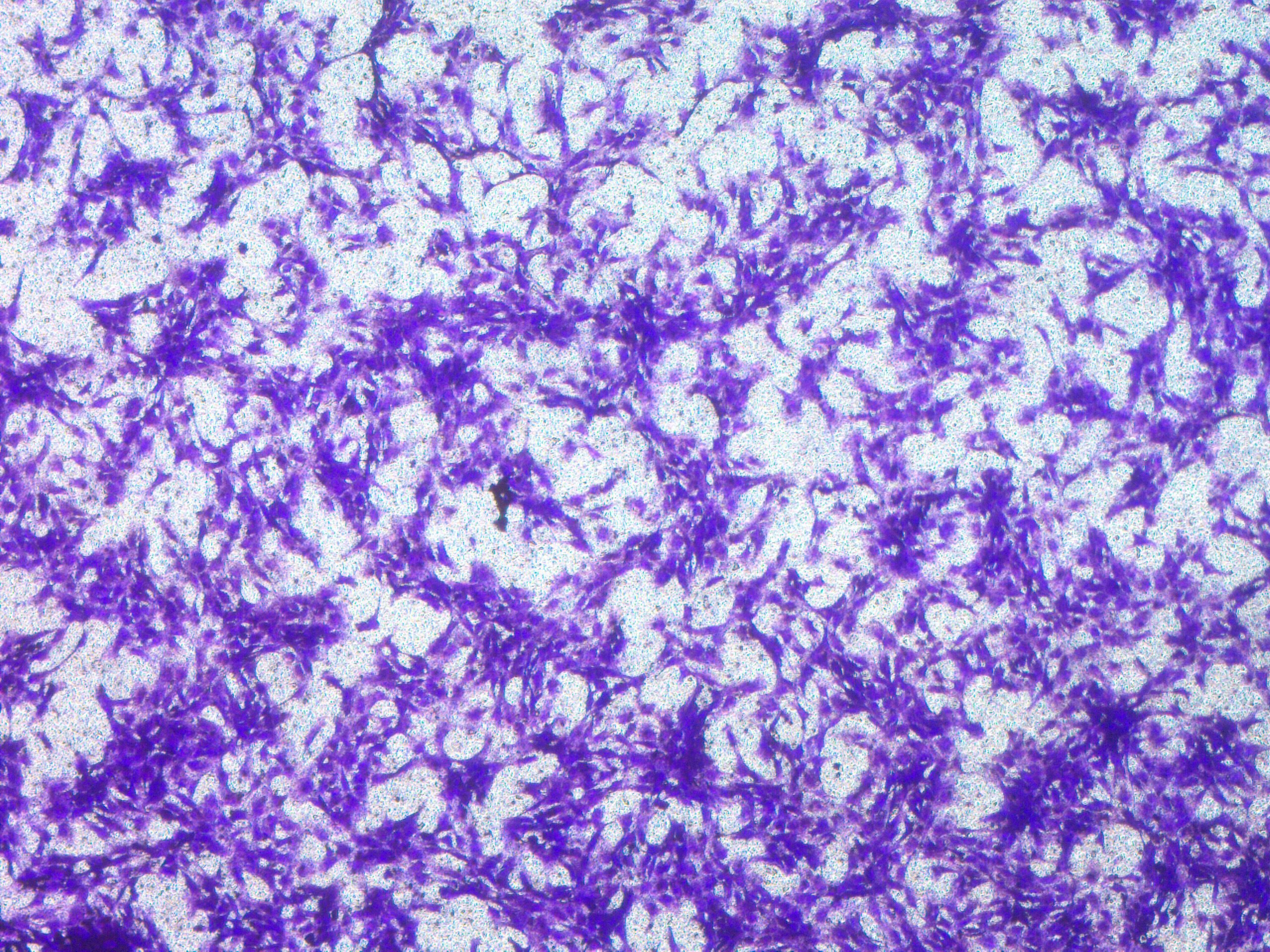

Supplement: Supplementary file 13 — Source data Fig. 2 [file 44321_2025_364_MOESM13_ESM.zip › 2A/A2.2_50nM.tif]

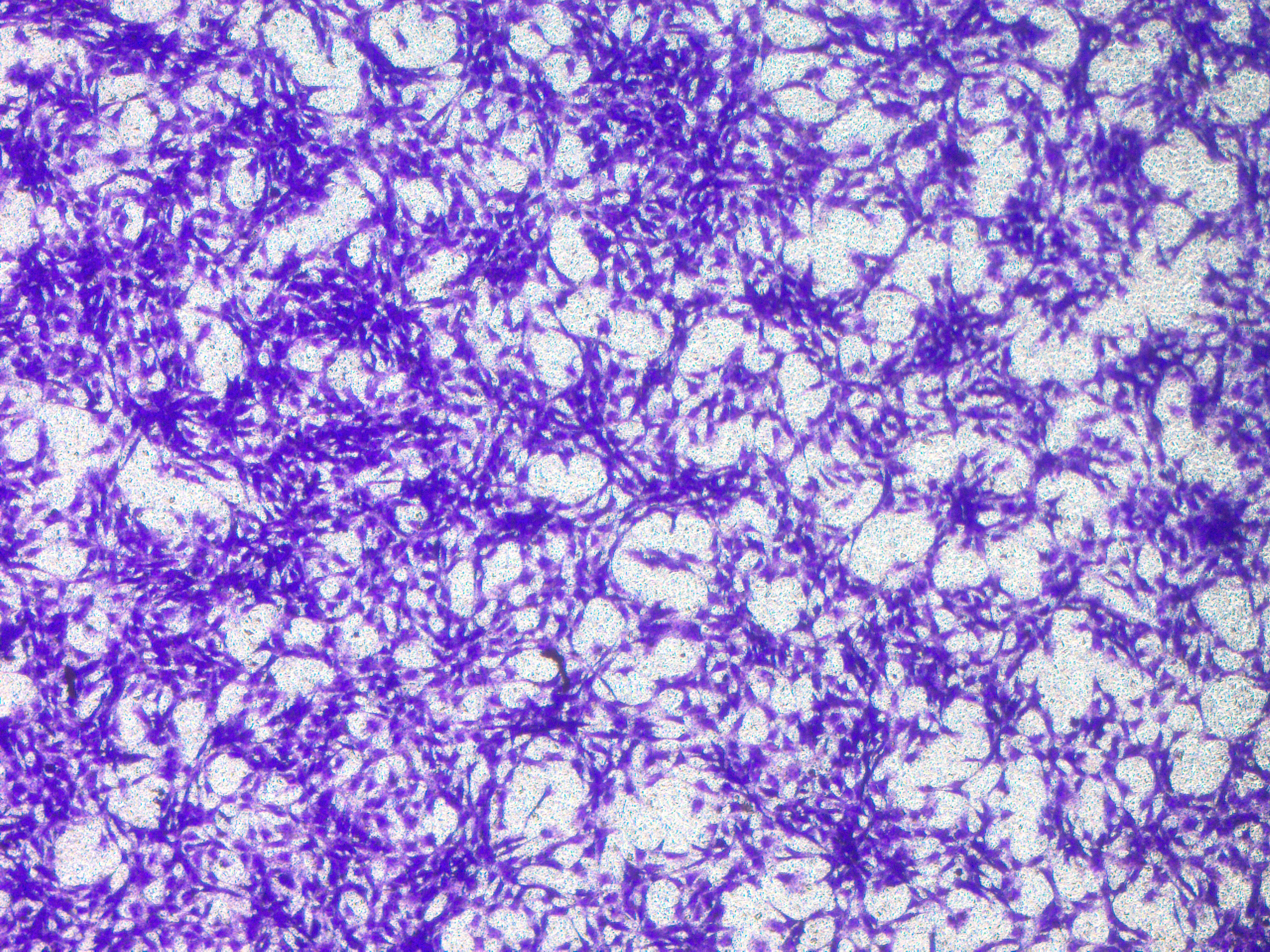

Supplement: Supplementary file 13 — Source data Fig. 2 [file 44321_2025_364_MOESM13_ESM.zip › 2A/A2.2_5nM.tif]

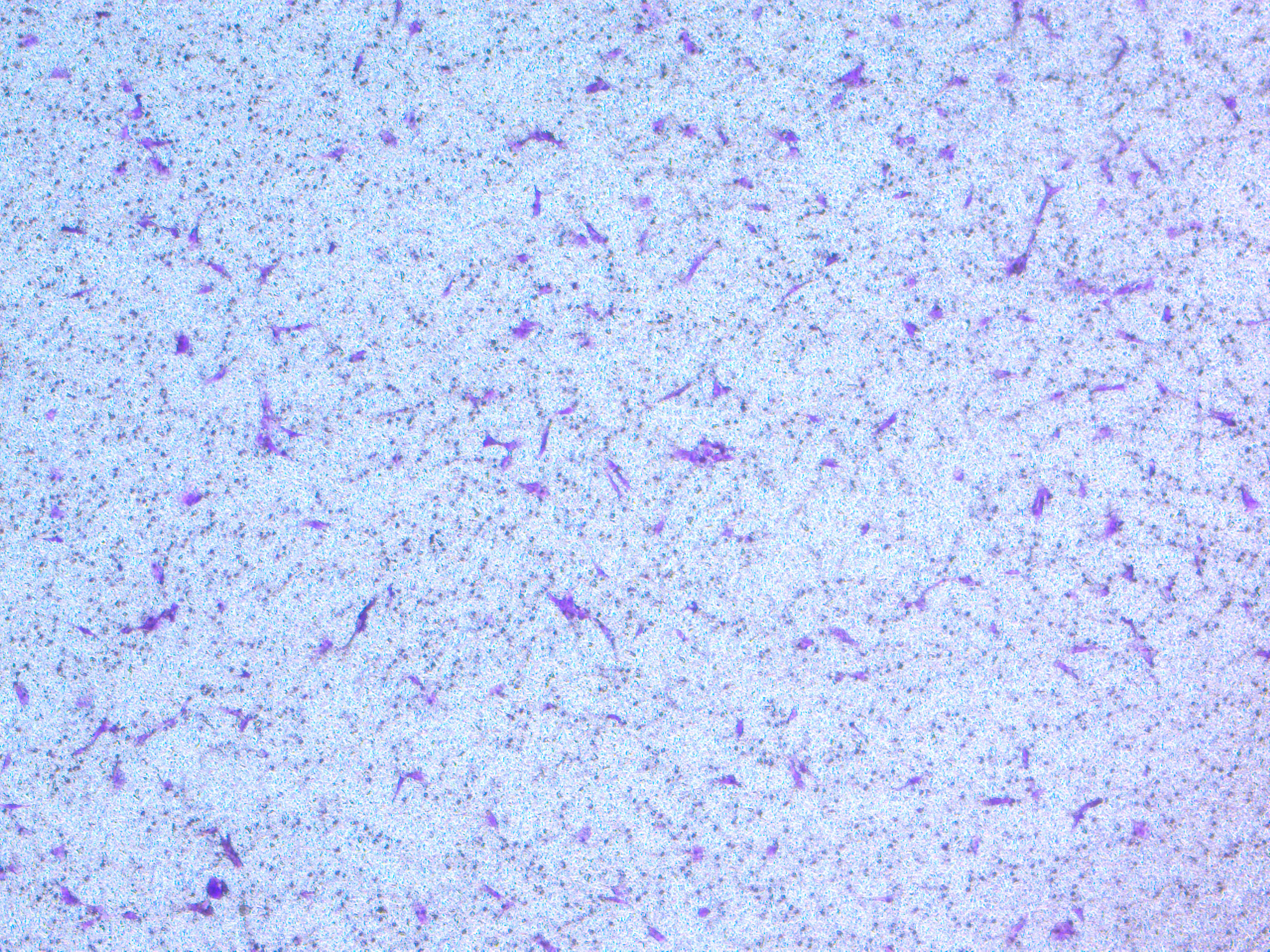

Supplement: Supplementary file 13 — Source data Fig. 2 [file 44321_2025_364_MOESM13_ESM.zip › 2A/B3.1_0nM.tif]

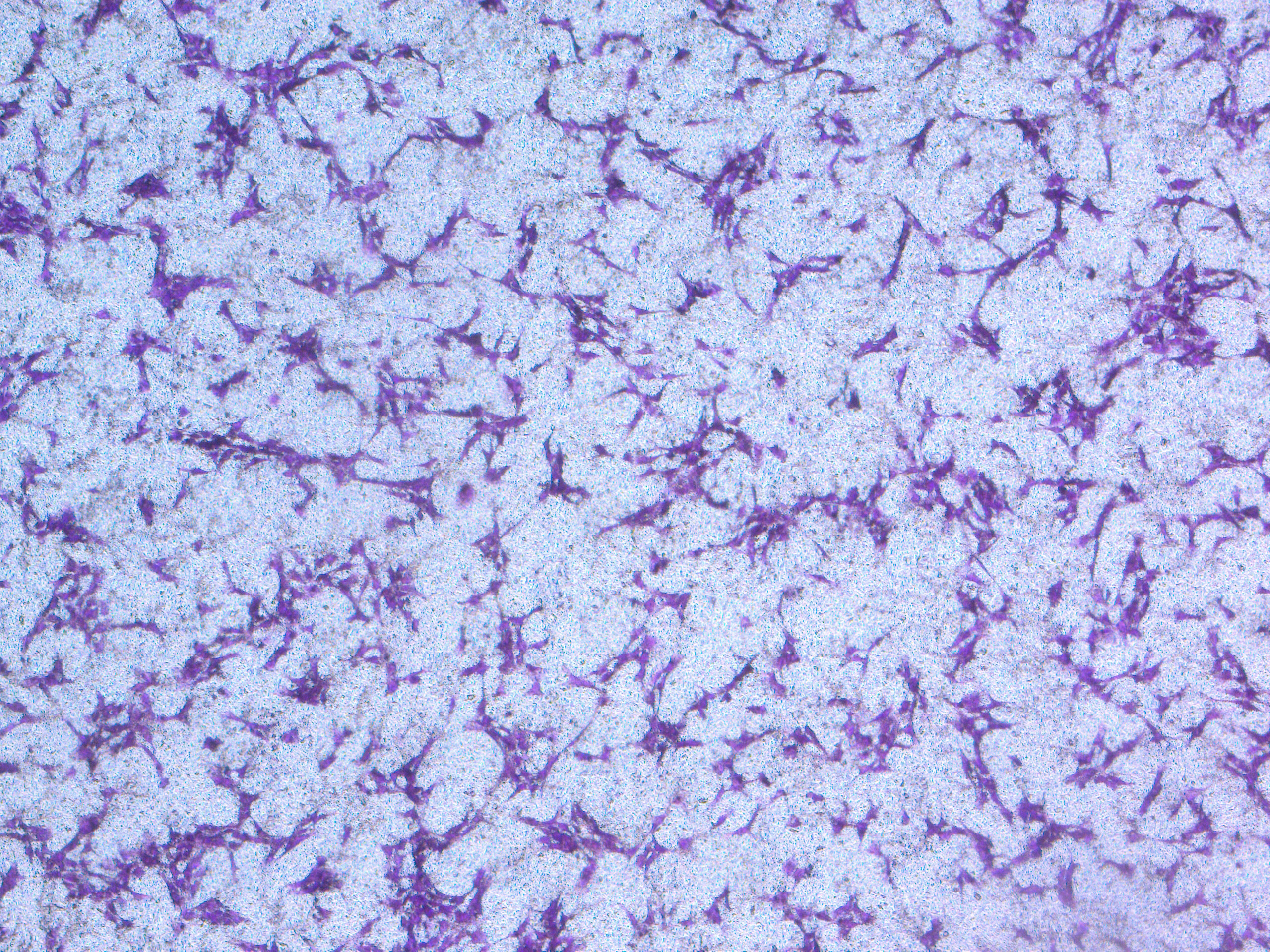

Supplement: Supplementary file 13 — Source data Fig. 2 [file 44321_2025_364_MOESM13_ESM.zip › 2A/B3.1_1.5nM.tif]

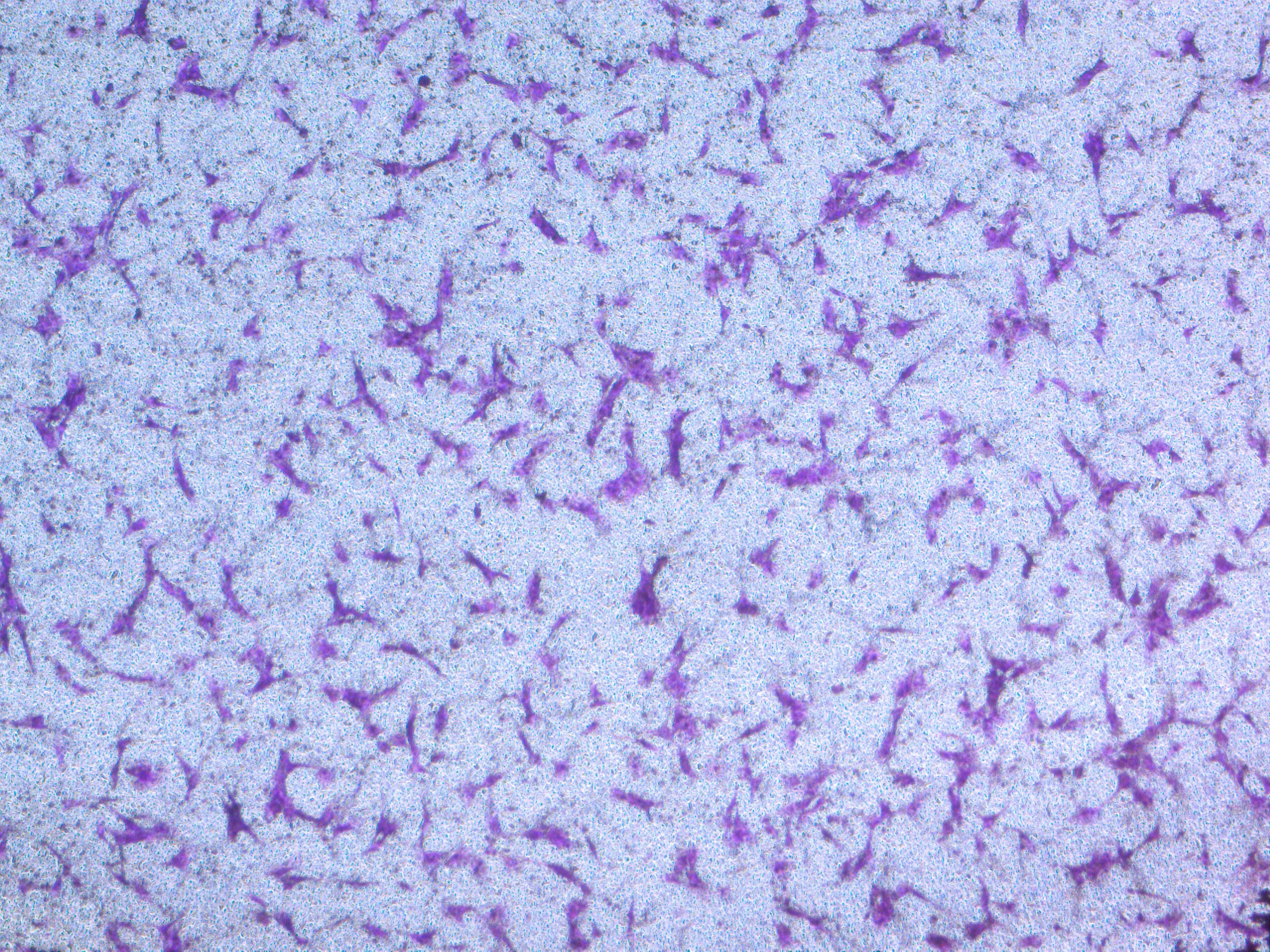

Supplement: Supplementary file 13 — Source data Fig. 2 [file 44321_2025_364_MOESM13_ESM.zip › 2A/B3.1_150nM.tif]

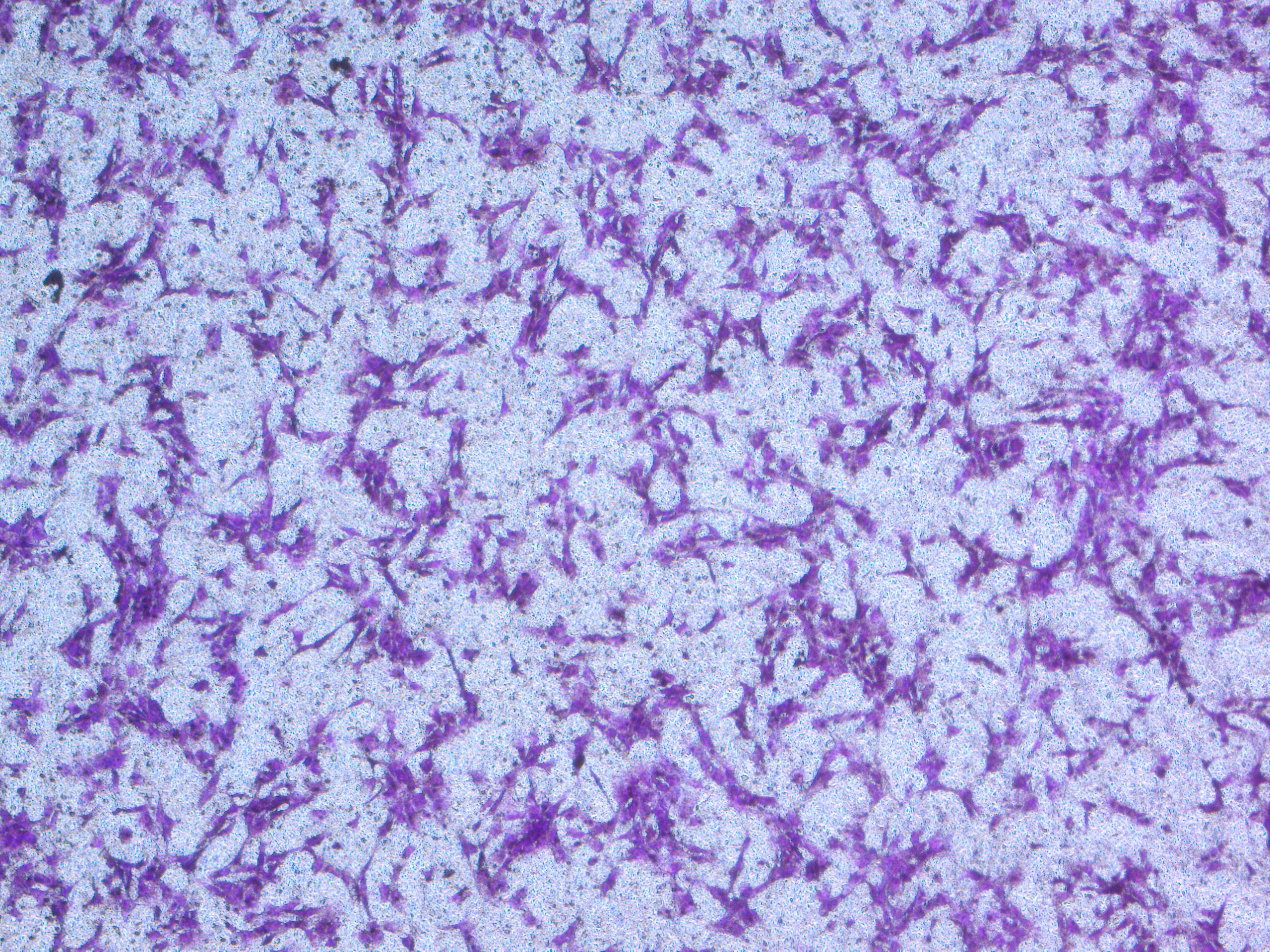

Supplement: Supplementary file 13 — Source data Fig. 2 [file 44321_2025_364_MOESM13_ESM.zip › 2A/B3.1_15nM.tif]

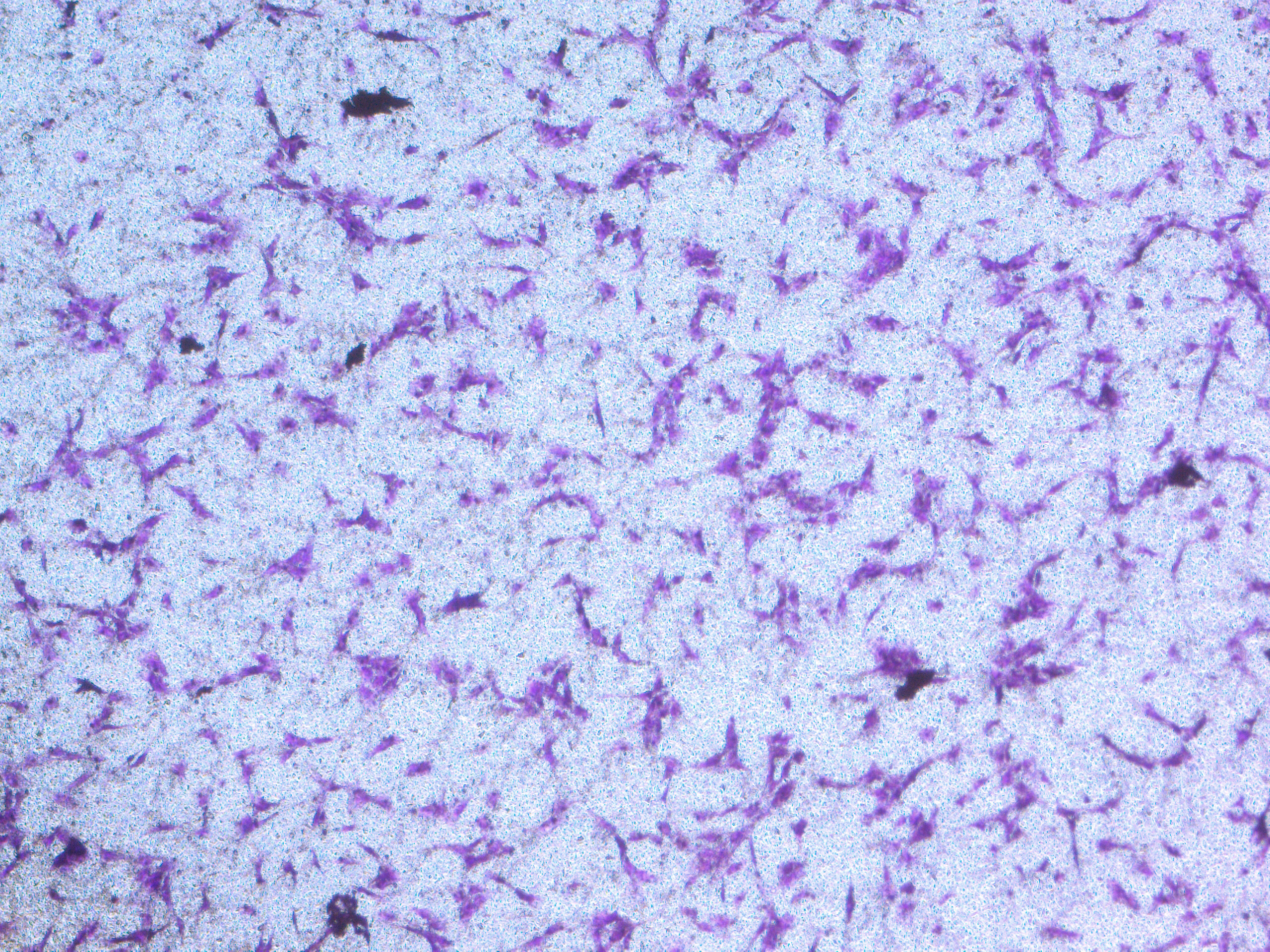

Supplement: Supplementary file 13 — Source data Fig. 2 [file 44321_2025_364_MOESM13_ESM.zip › 2A/B3.1_50nM.tif]

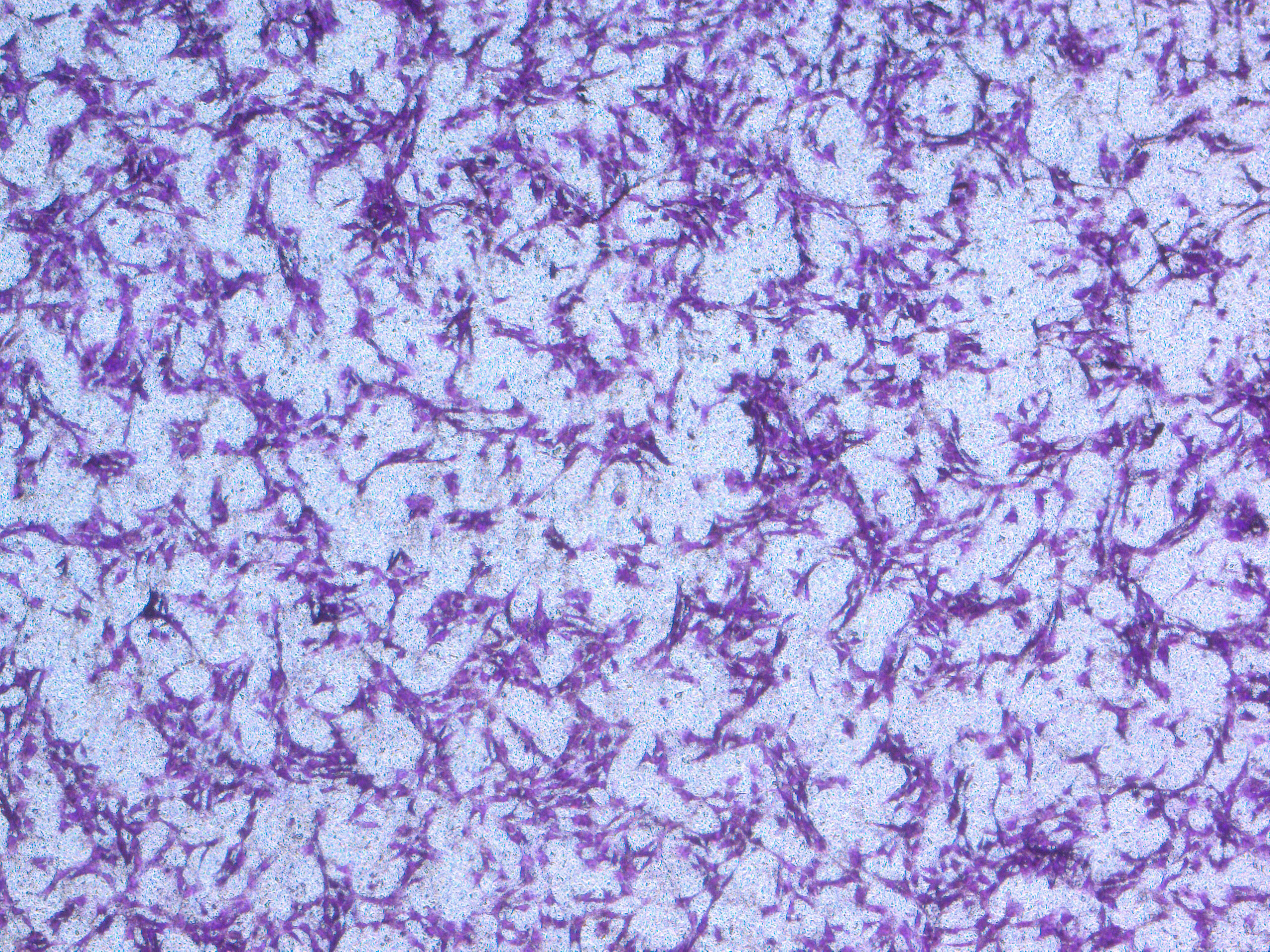

Supplement: Supplementary file 13 — Source data Fig. 2 [file 44321_2025_364_MOESM13_ESM.zip › 2A/B3.1_5nM.tif]

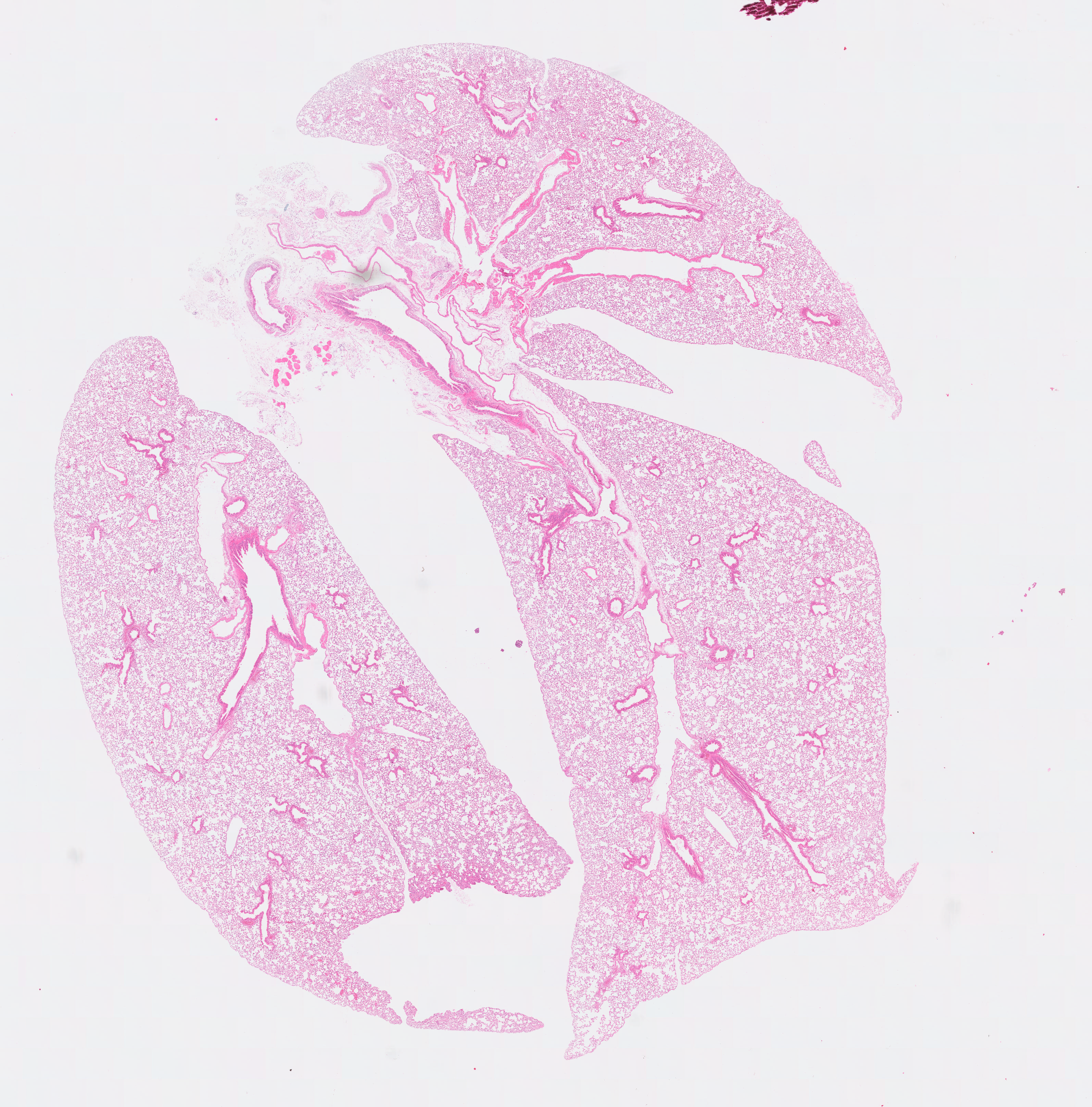

Supplement: Supplementary file 17 — Source data Fig. 6 [file 44321_2025_364_MOESM17_ESM.zip › 6F/7 day EF rescue (15 nM).tif]

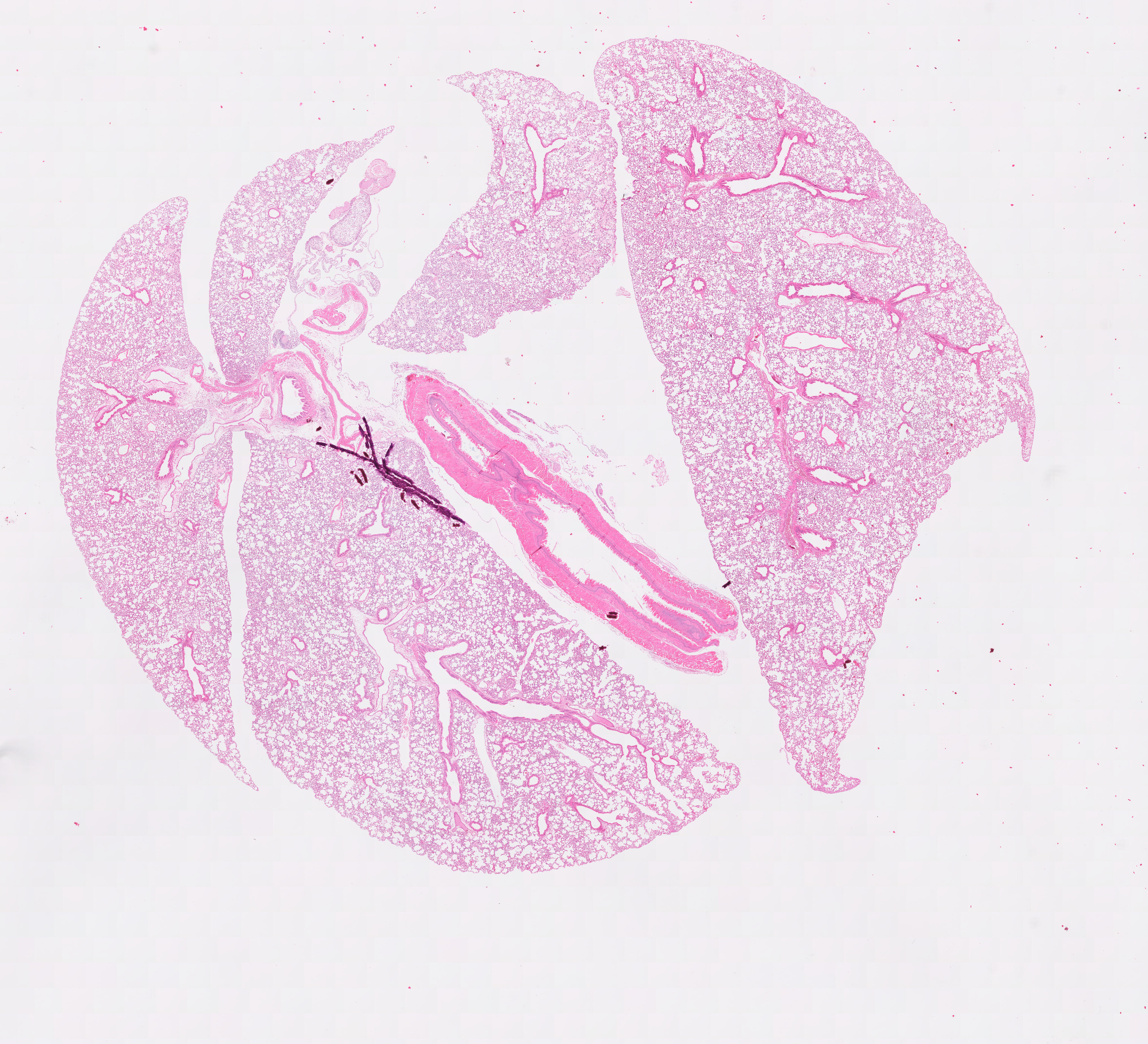

Supplement: Supplementary file 17 — Source data Fig. 6 [file 44321_2025_364_MOESM17_ESM.zip › 6F/Control.tif]

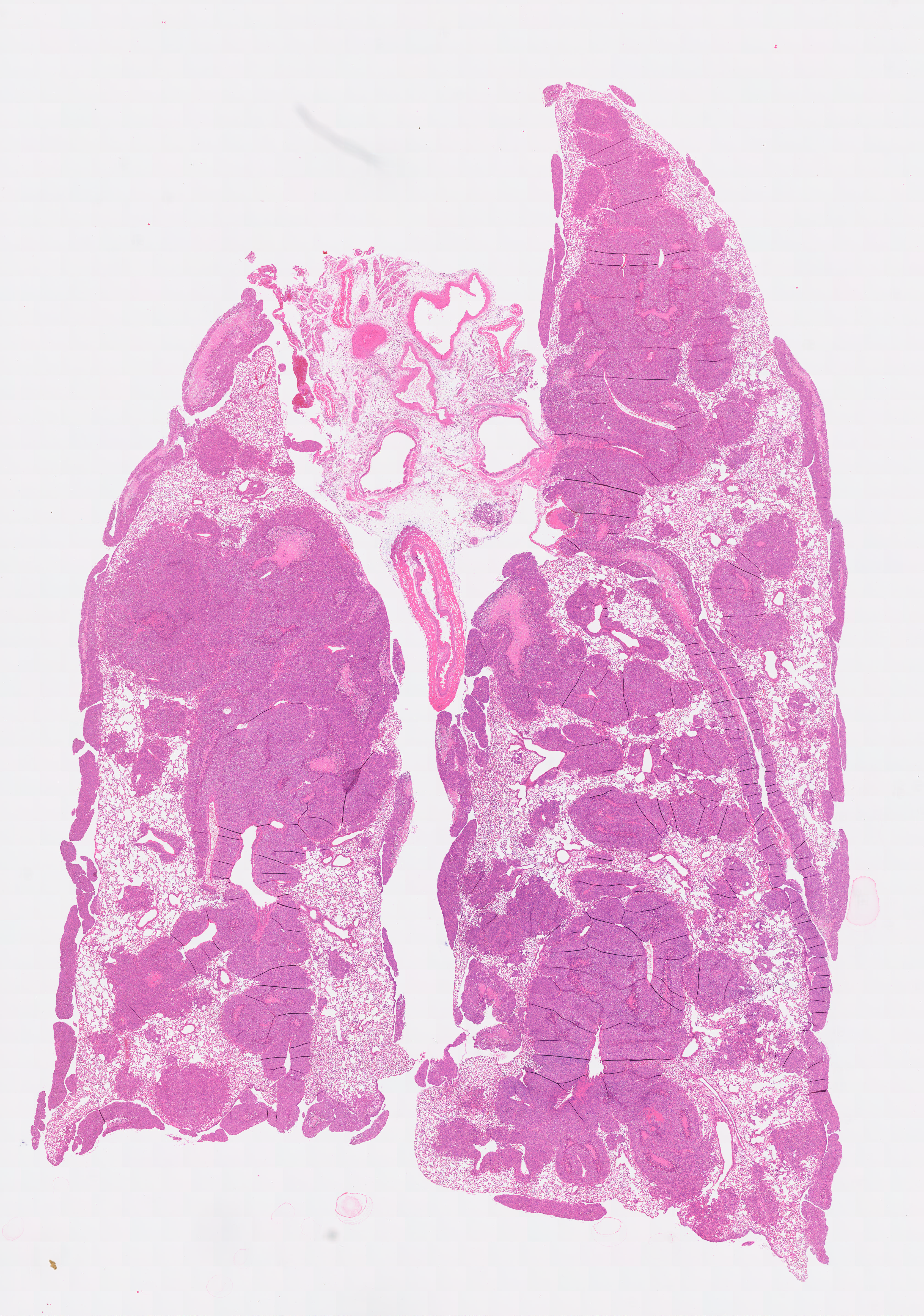

Supplement: Supplementary file 17 — Source data Fig. 6 [file 44321_2025_364_MOESM17_ESM.zip › 6F/dTAGV-1 (15nM).tif]

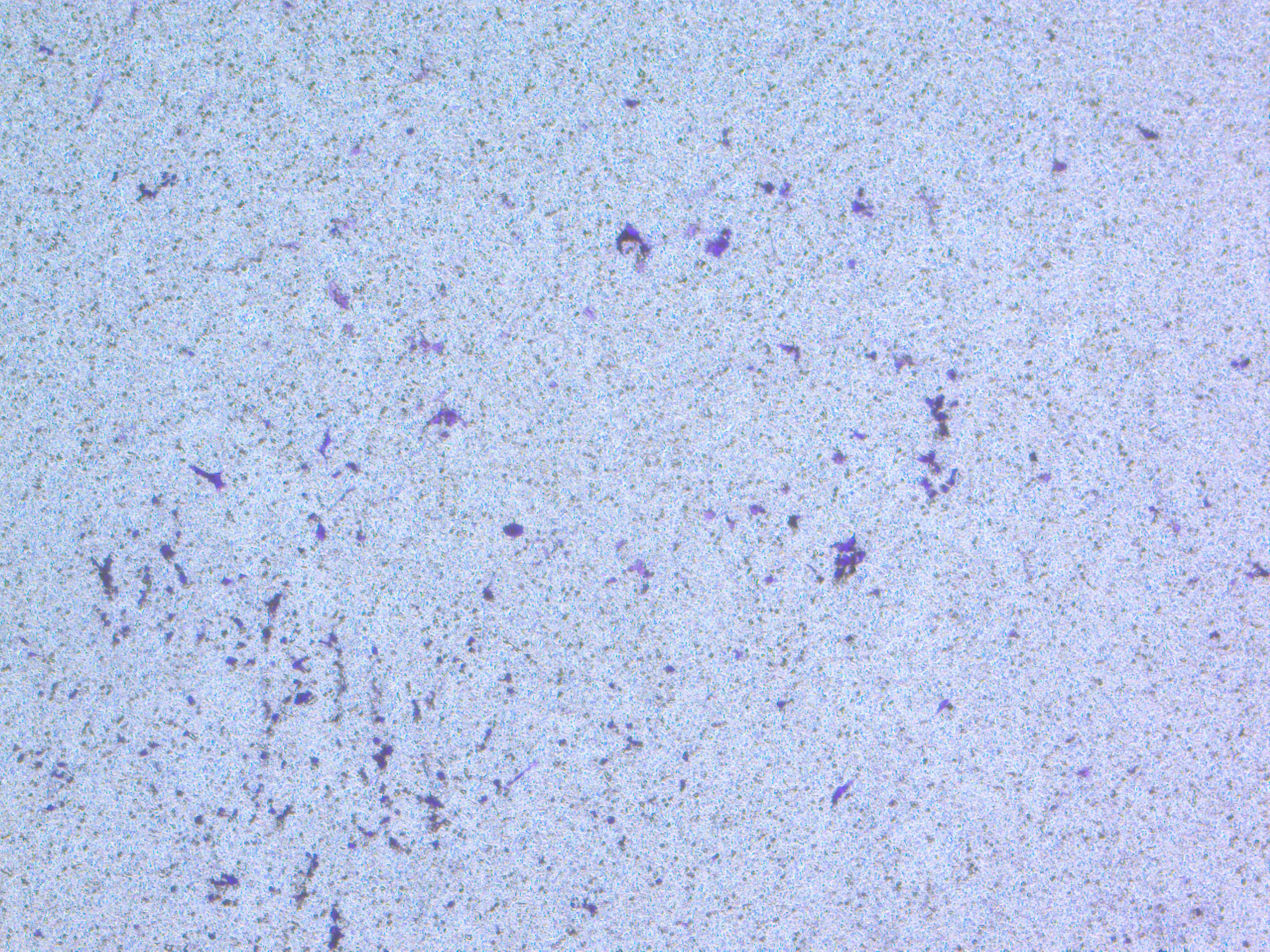

Supplement: Supplementary file 17 — Source data Fig. 6 [file 44321_2025_364_MOESM17_ESM.zip › 6A/A2.2_21d+7dwashout_0nM.tif]

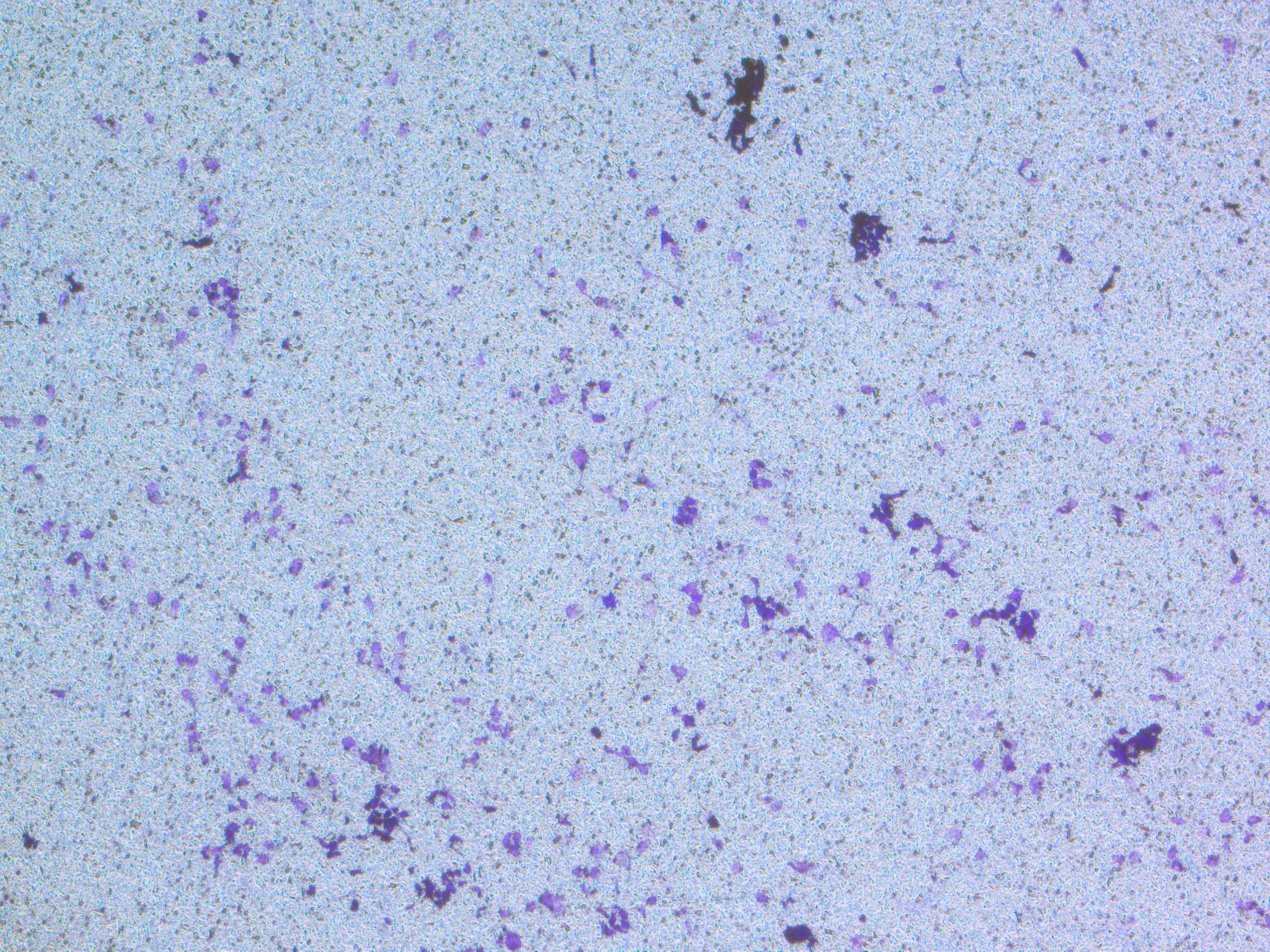

Supplement: Supplementary file 17 — Source data Fig. 6 [file 44321_2025_364_MOESM17_ESM.zip › 6A/A2.2_21d+7dwashout_1.5nM.tif]

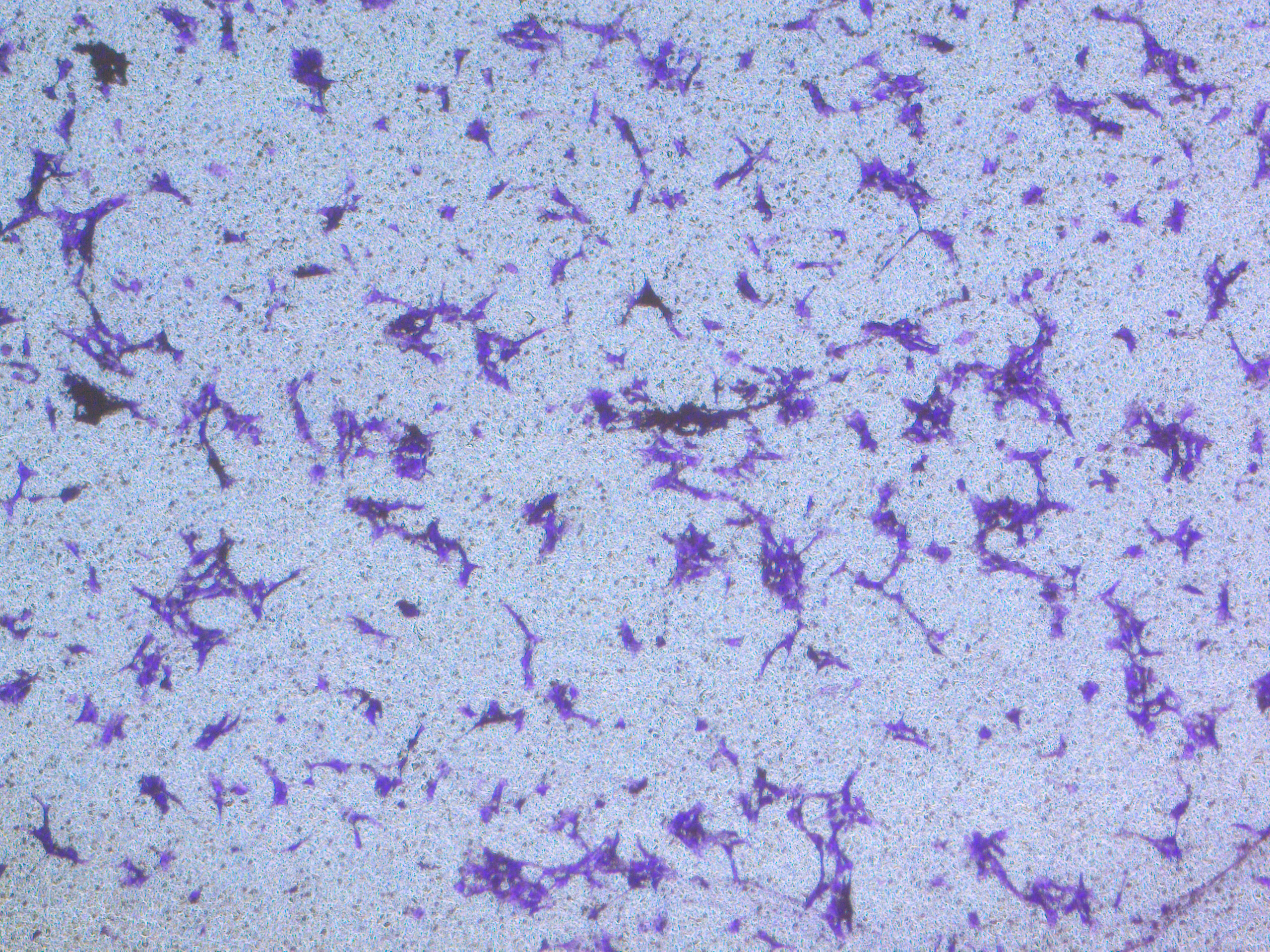

Supplement: Supplementary file 17 — Source data Fig. 6 [file 44321_2025_364_MOESM17_ESM.zip › 6A/A2.2_21d+7dwashout_150nM.tif]

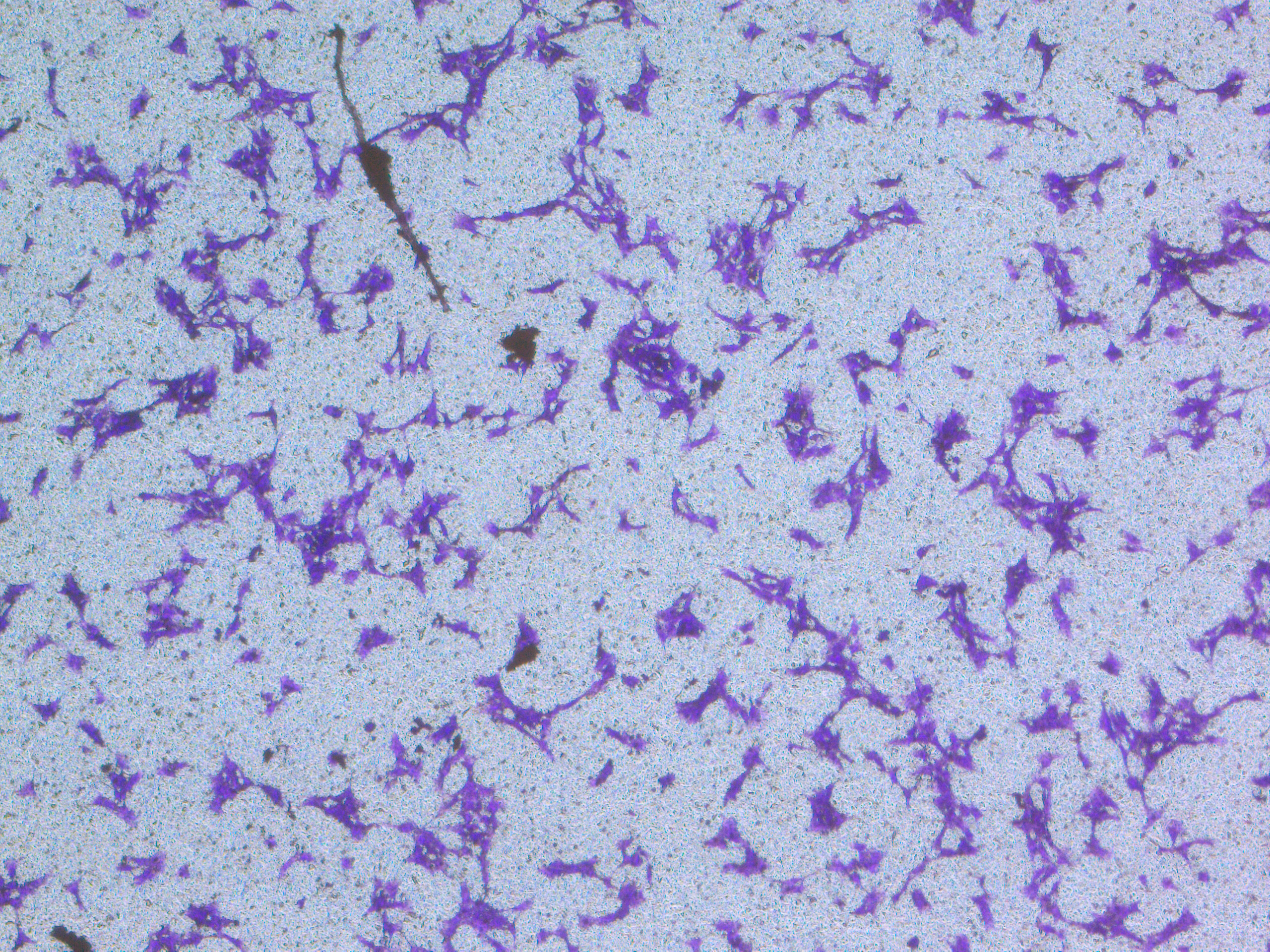

Supplement: Supplementary file 17 — Source data Fig. 6 [file 44321_2025_364_MOESM17_ESM.zip › 6A/A2.2_21d+7dwashout_15nM.tif]

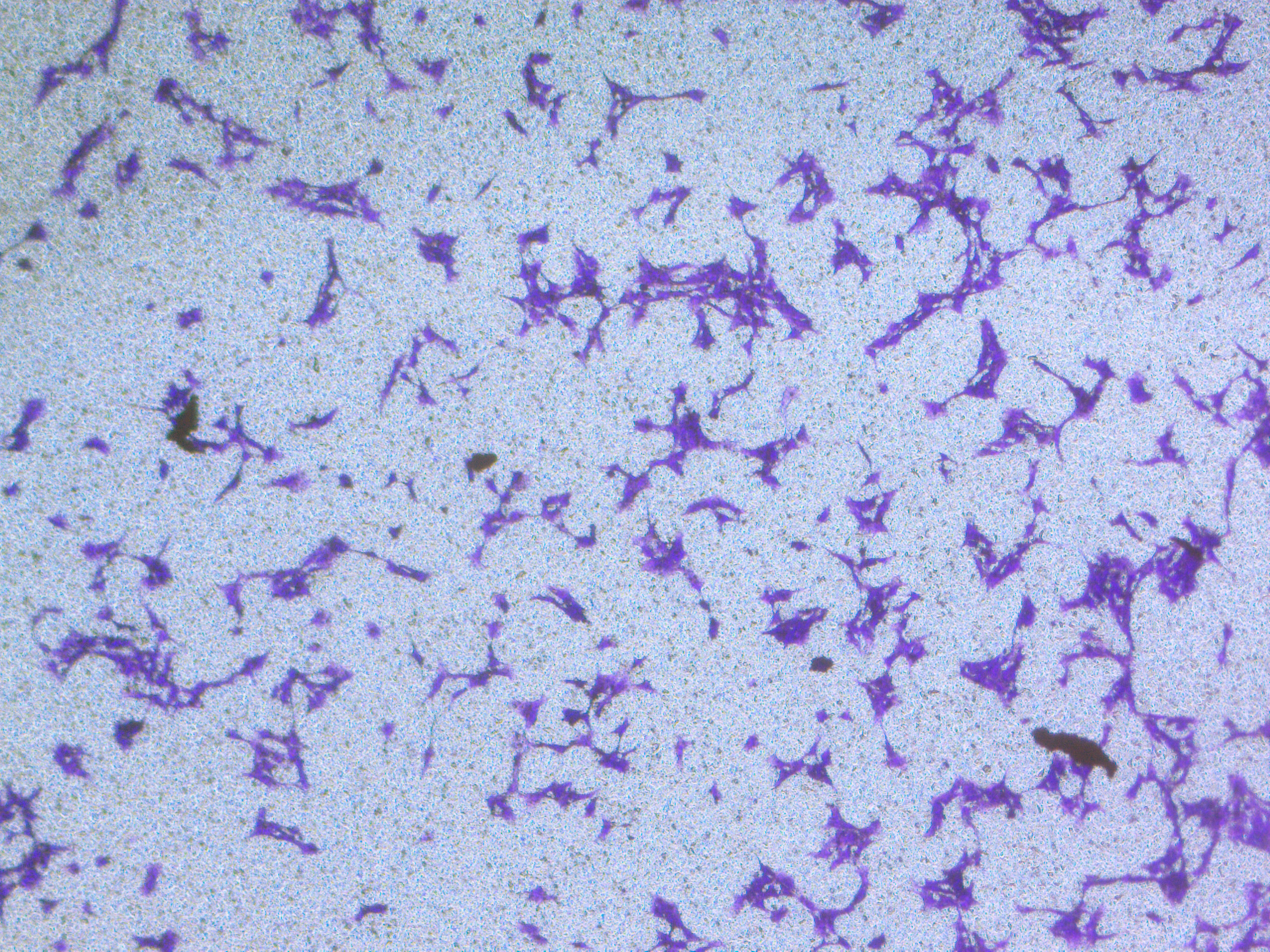

Supplement: Supplementary file 17 — Source data Fig. 6 [file 44321_2025_364_MOESM17_ESM.zip › 6A/A2.2_21d+7dwashout_50nM.tif]

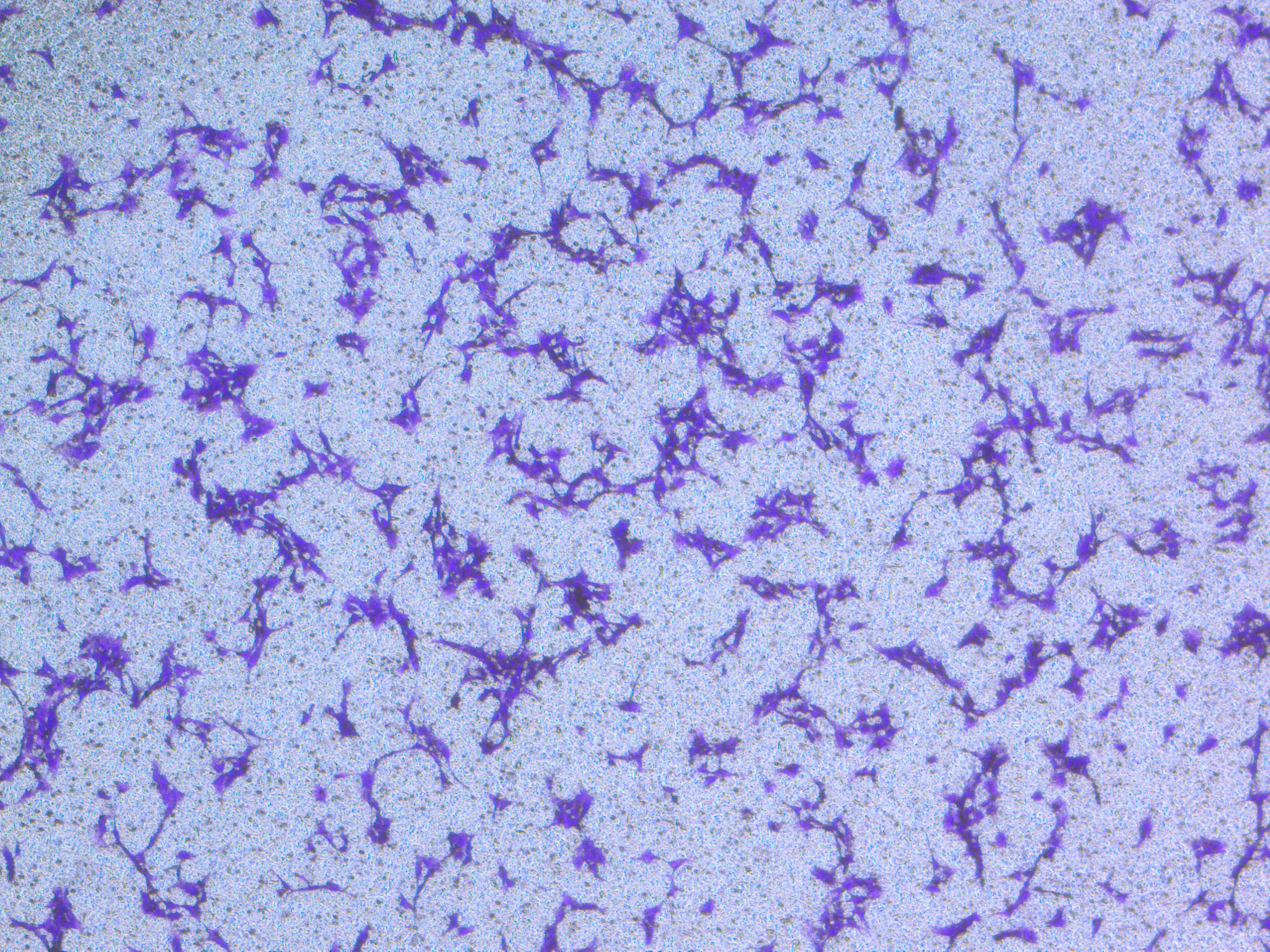

Supplement: Supplementary file 17 — Source data Fig. 6 [file 44321_2025_364_MOESM17_ESM.zip › 6A/A2.2_21d+7dwashout_5nM.tif]

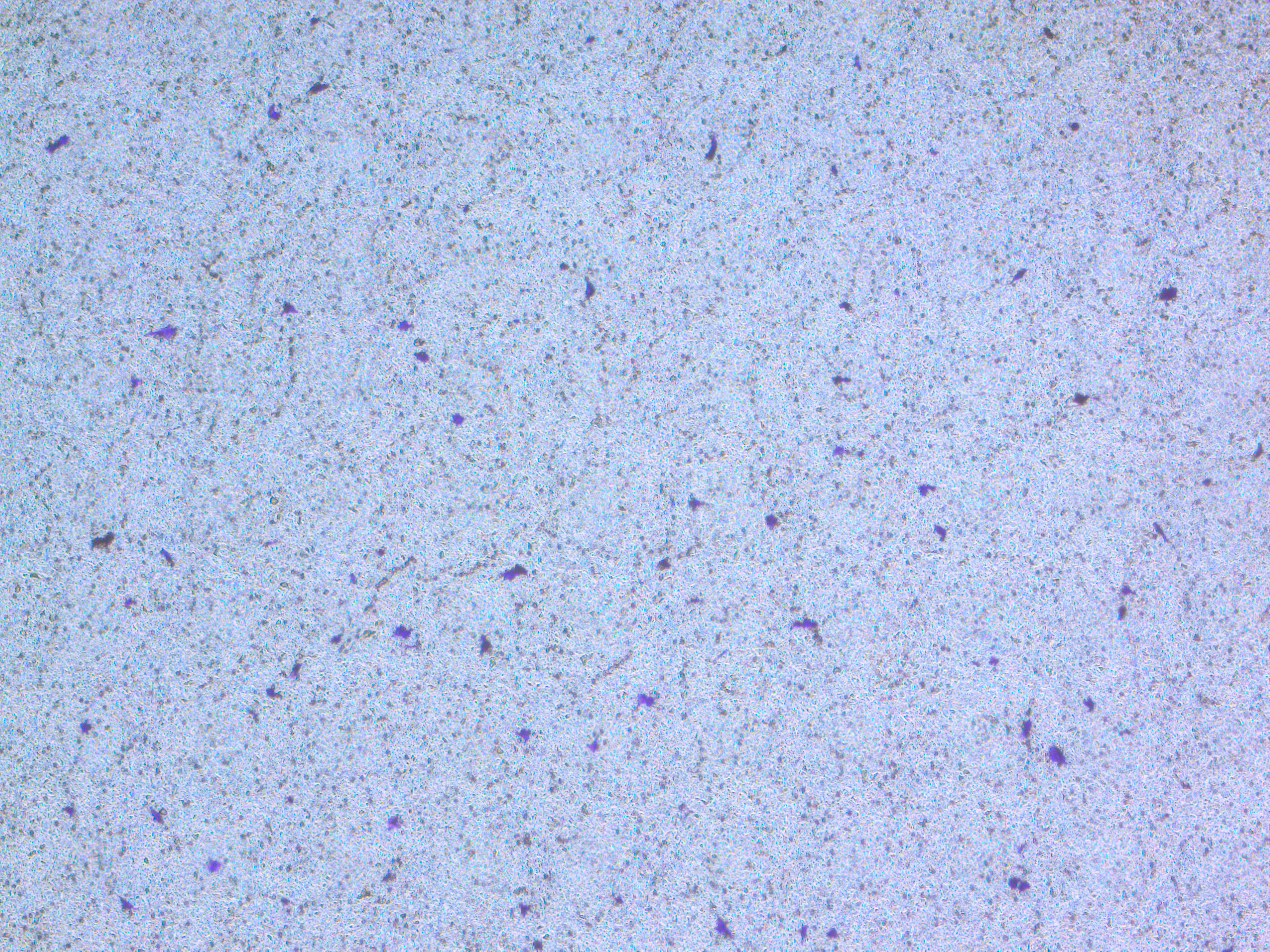

Supplement: Supplementary file 17 — Source data Fig. 6 [file 44321_2025_364_MOESM17_ESM.zip › 6A/A2.2_21d_0nM.tif]

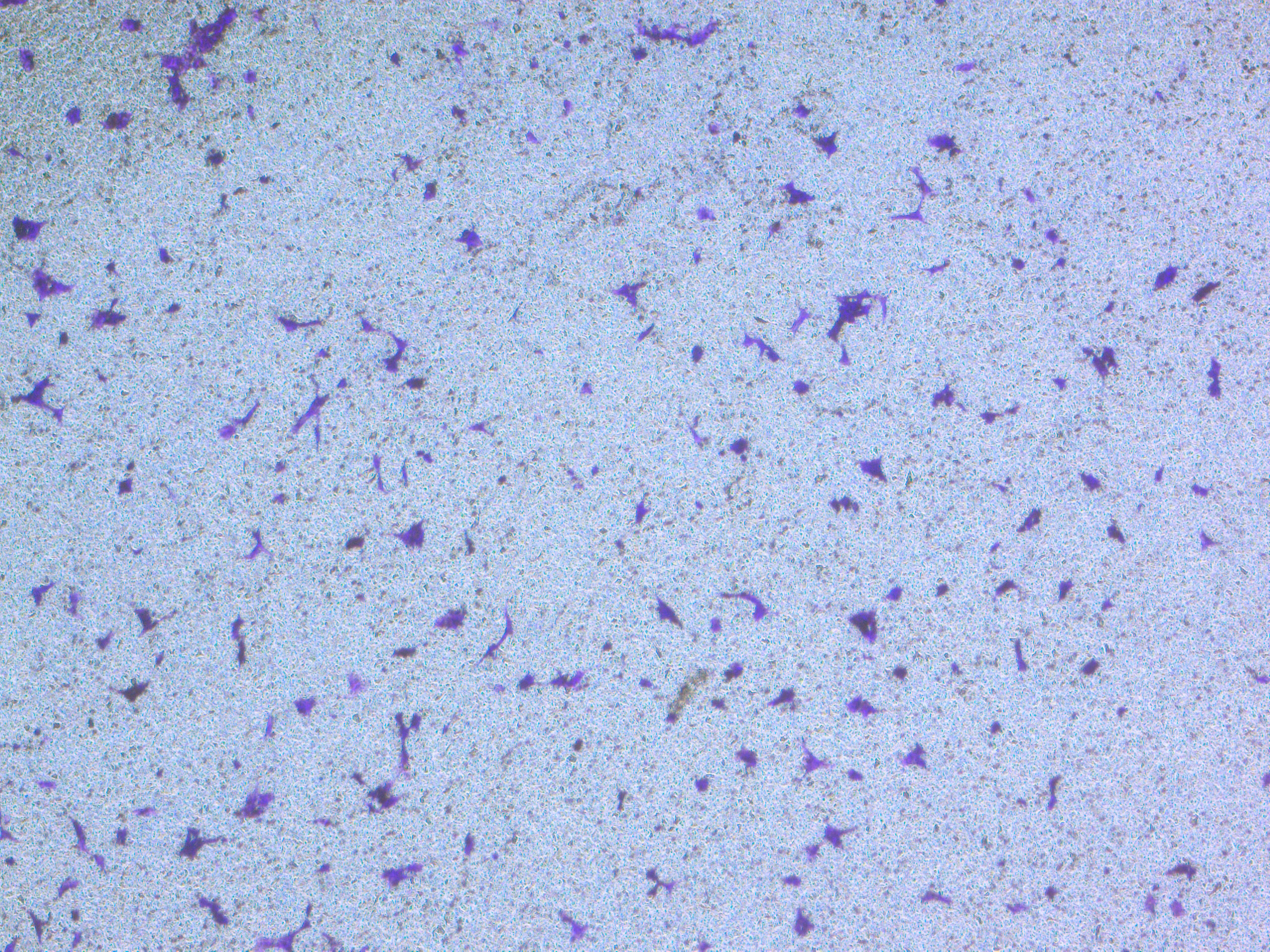

Supplement: Supplementary file 17 — Source data Fig. 6 [file 44321_2025_364_MOESM17_ESM.zip › 6A/A2.2_21d_1.5nM.tif]

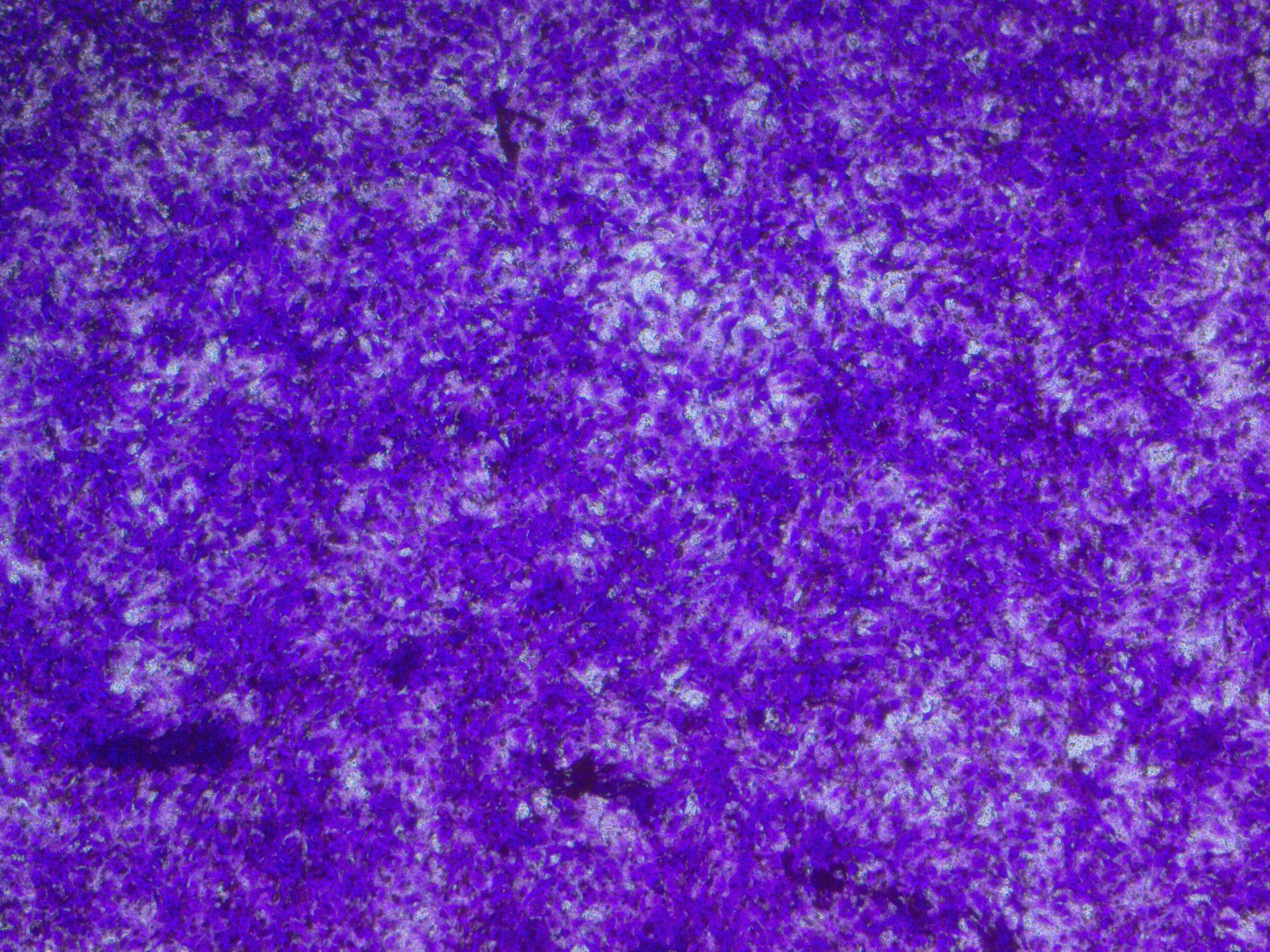

Supplement: Supplementary file 17 — Source data Fig. 6 [file 44321_2025_364_MOESM17_ESM.zip › 6A/A2.2_21d_150nM.tif]

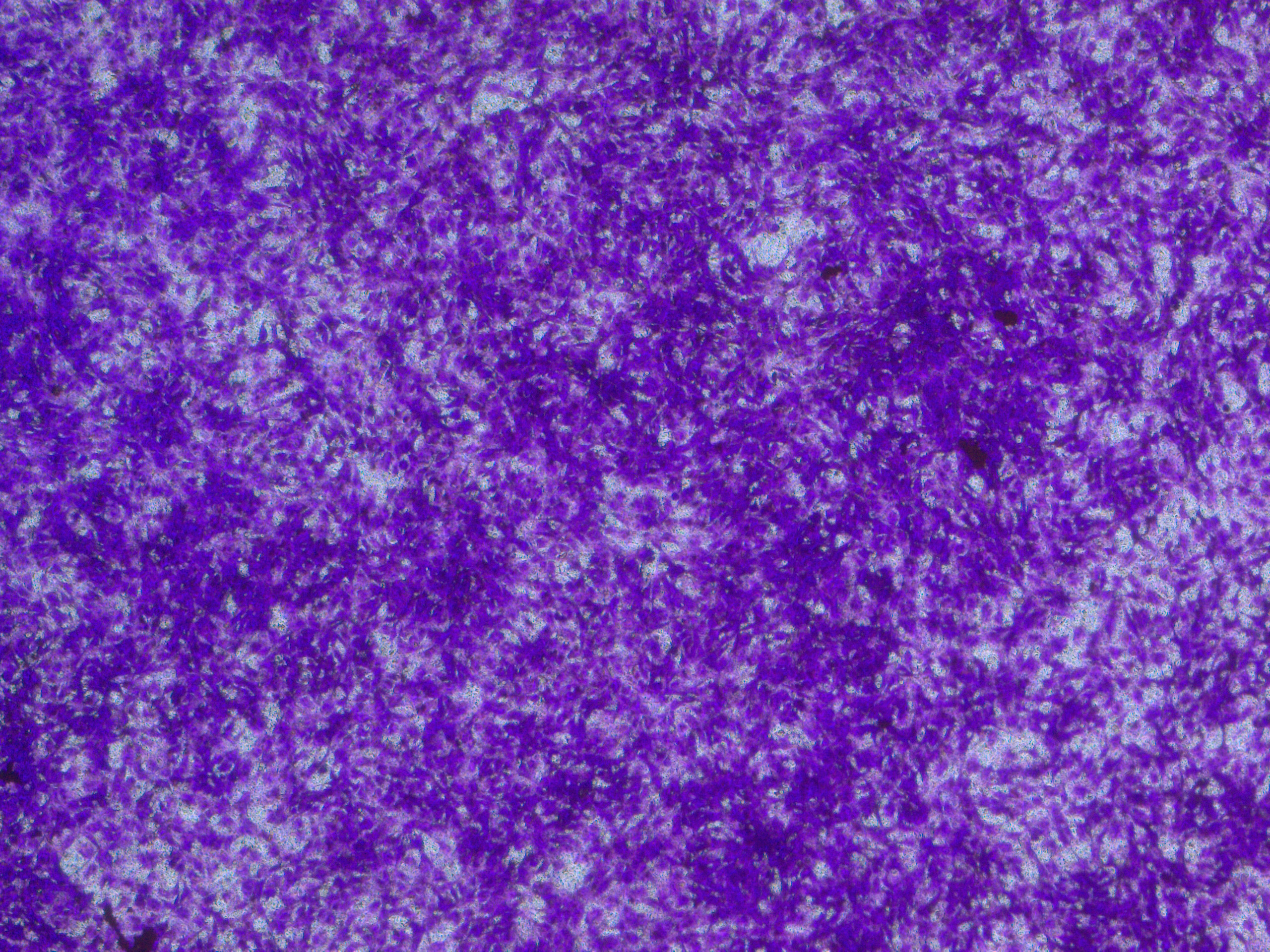

Supplement: Supplementary file 17 — Source data Fig. 6 [file 44321_2025_364_MOESM17_ESM.zip › 6A/A2.2_21d_15nM.tif]

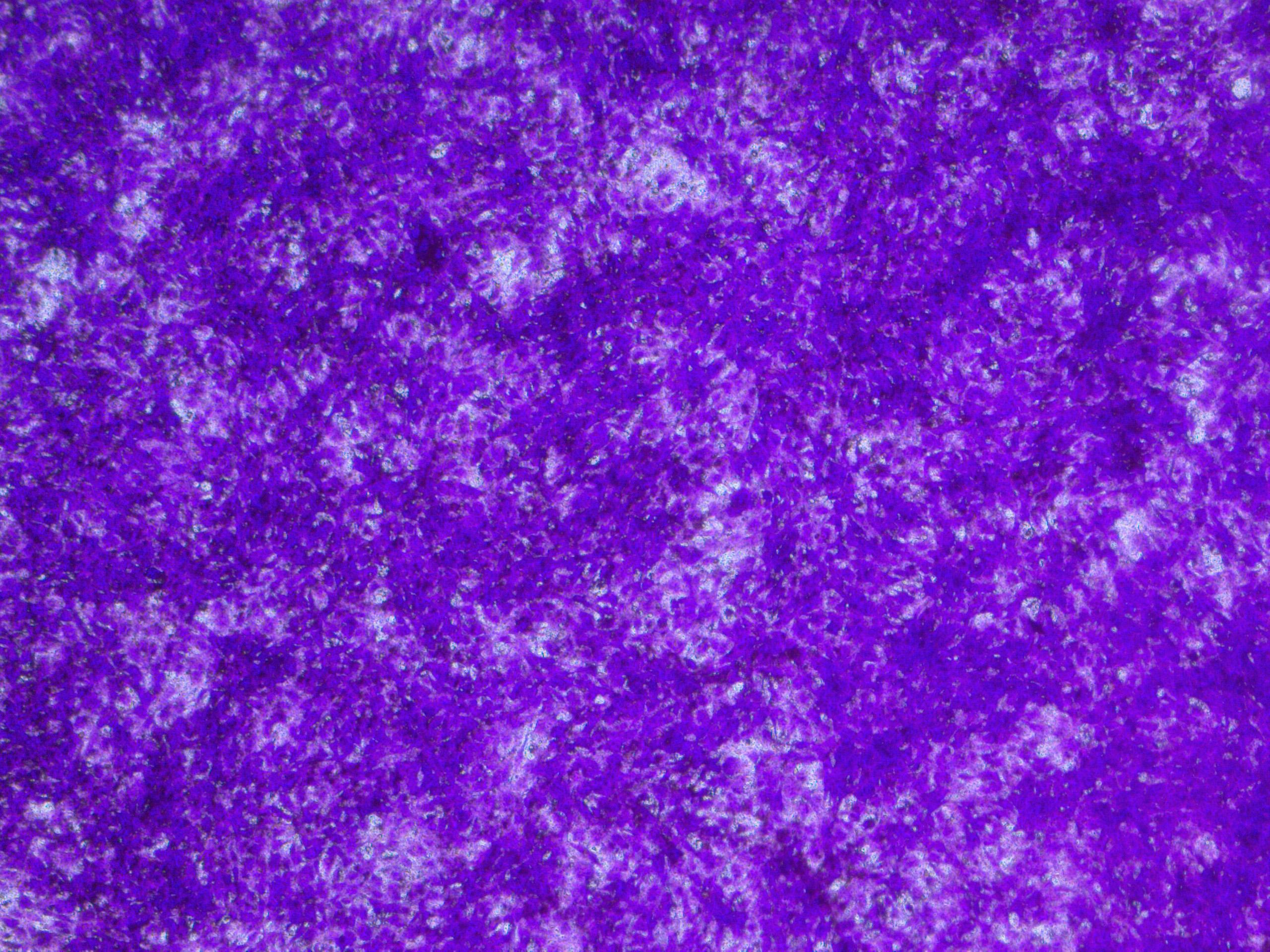

Supplement: Supplementary file 17 — Source data Fig. 6 [file 44321_2025_364_MOESM17_ESM.zip › 6A/A2.2_21d_50nM.tif]

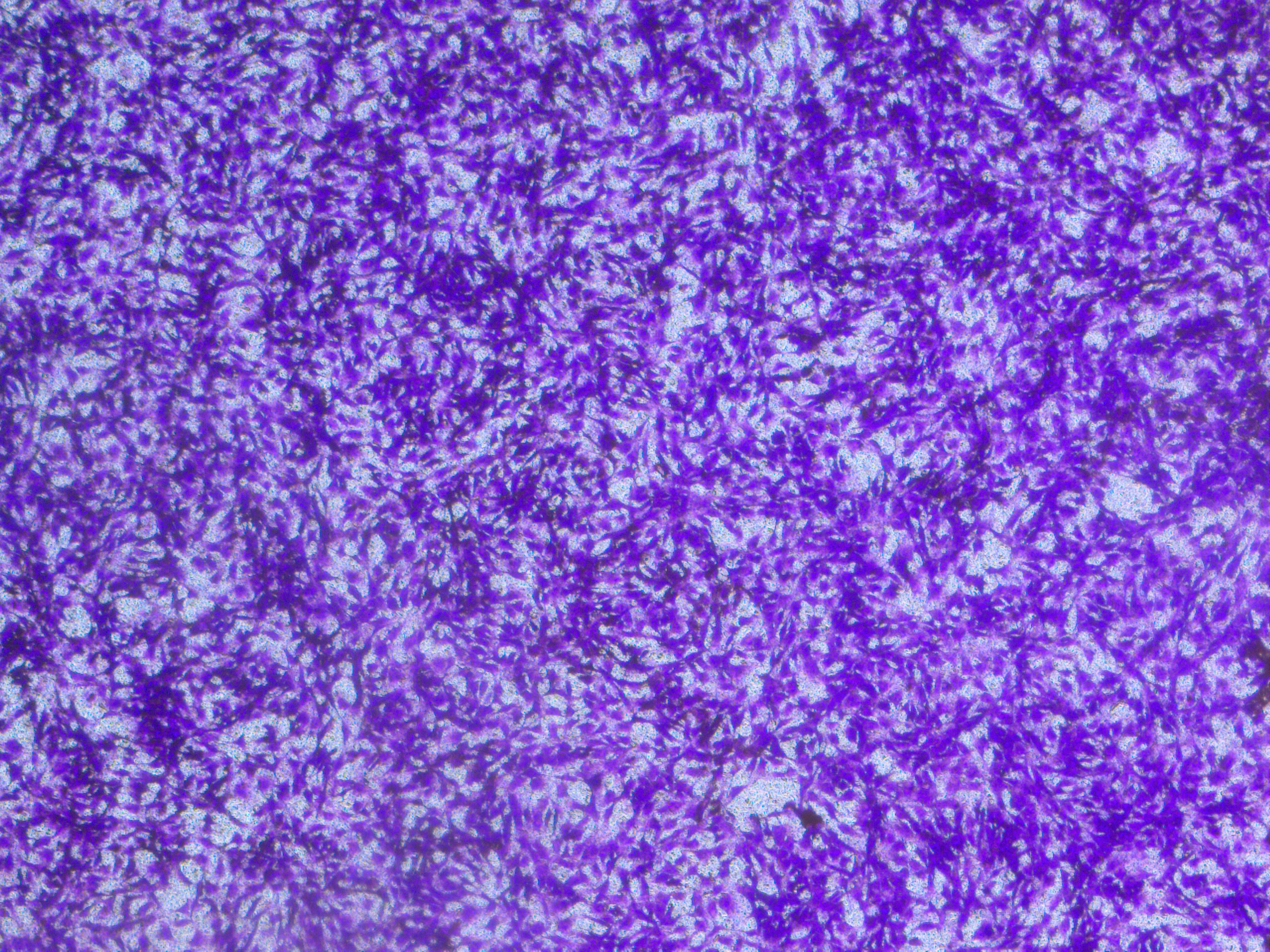

Supplement: Supplementary file 17 — Source data Fig. 6 [file 44321_2025_364_MOESM17_ESM.zip › 6A/A2.2_21d_5nM.tif]

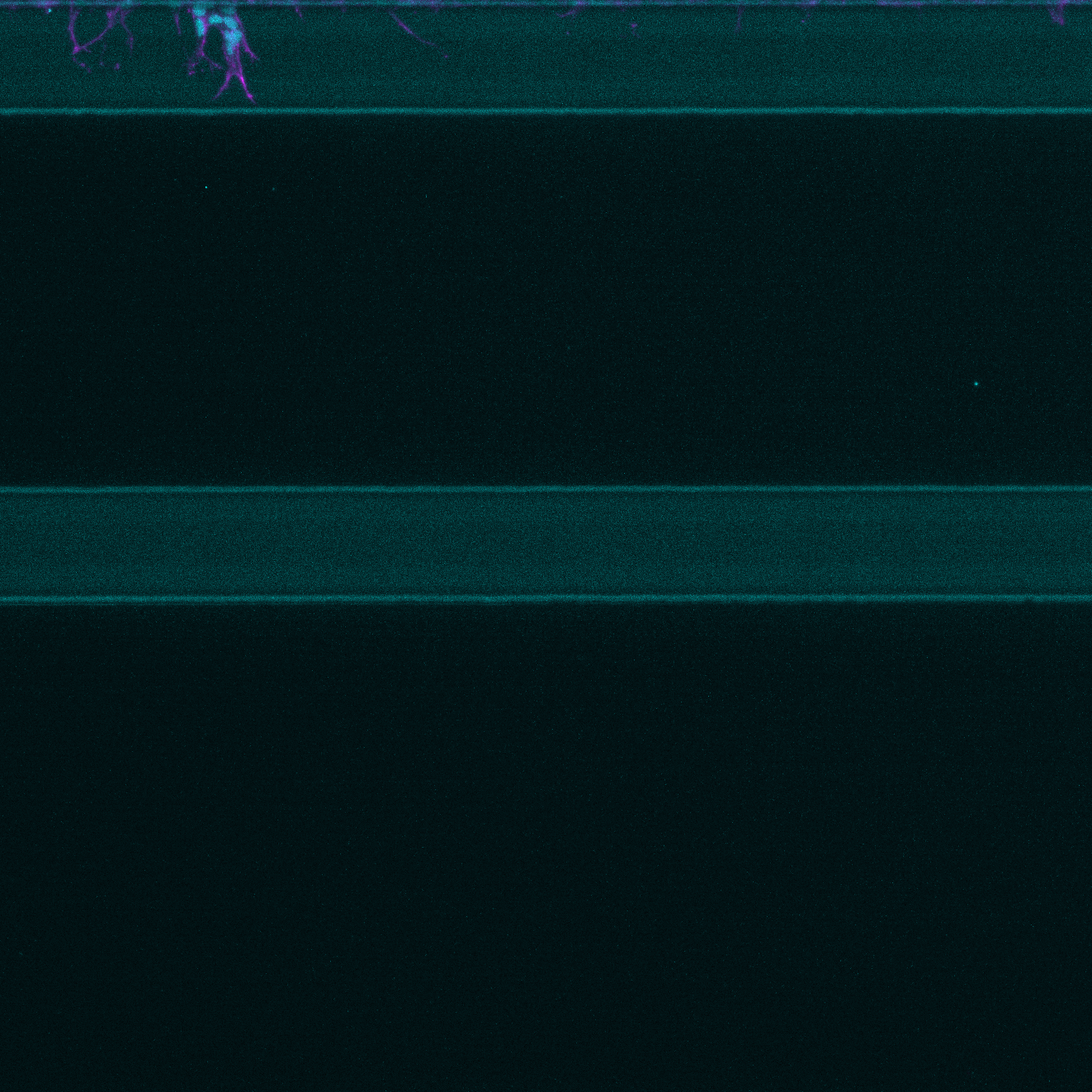

Supplement: Supplementary file 17 — Source data Fig. 6 [file 44321_2025_364_MOESM17_ESM.zip › 6B/A2.2_21d_0nM+7d_Washout_.tif]

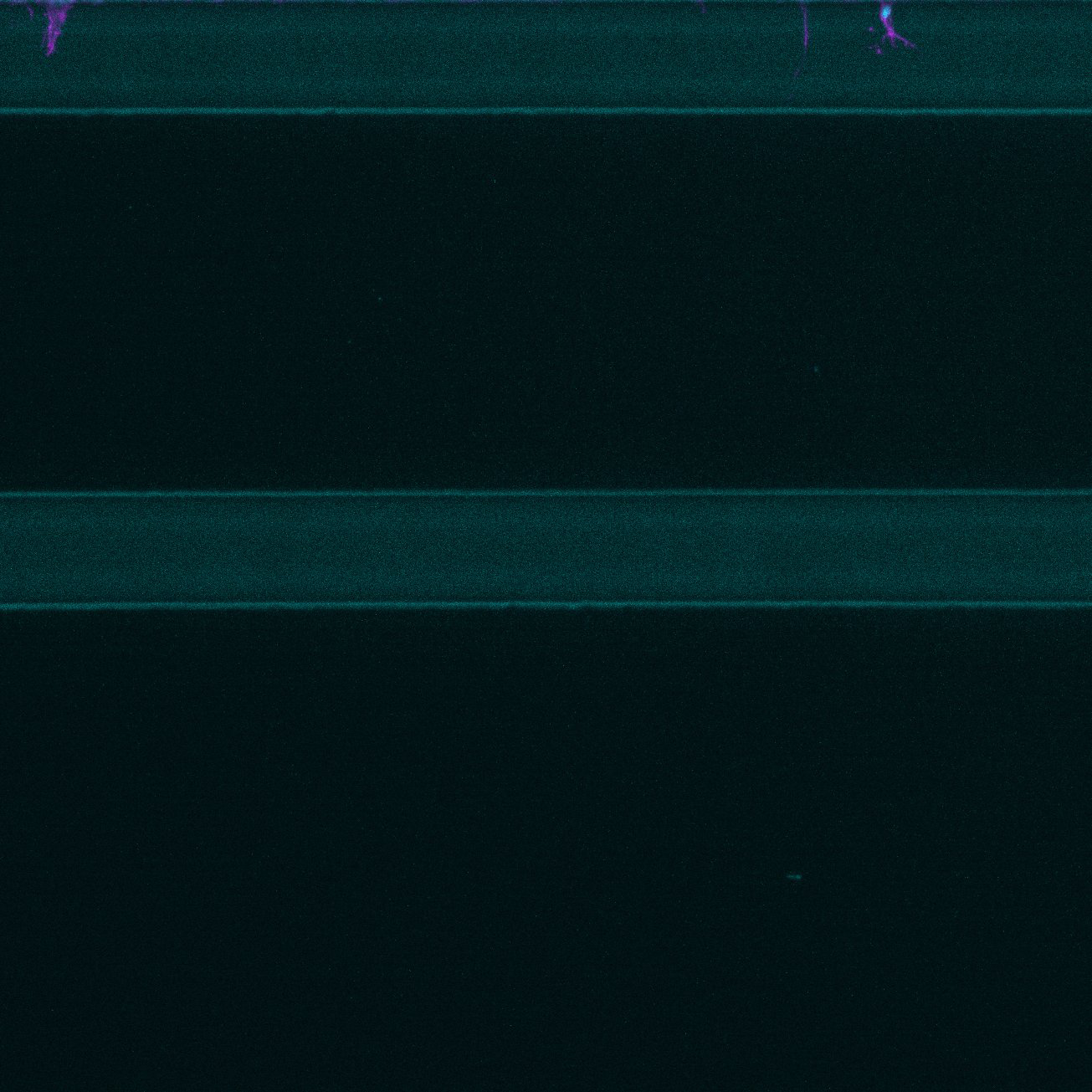

Supplement: Supplementary file 17 — Source data Fig. 6 [file 44321_2025_364_MOESM17_ESM.zip › 6B/A2.2_21d_0nM.tif]

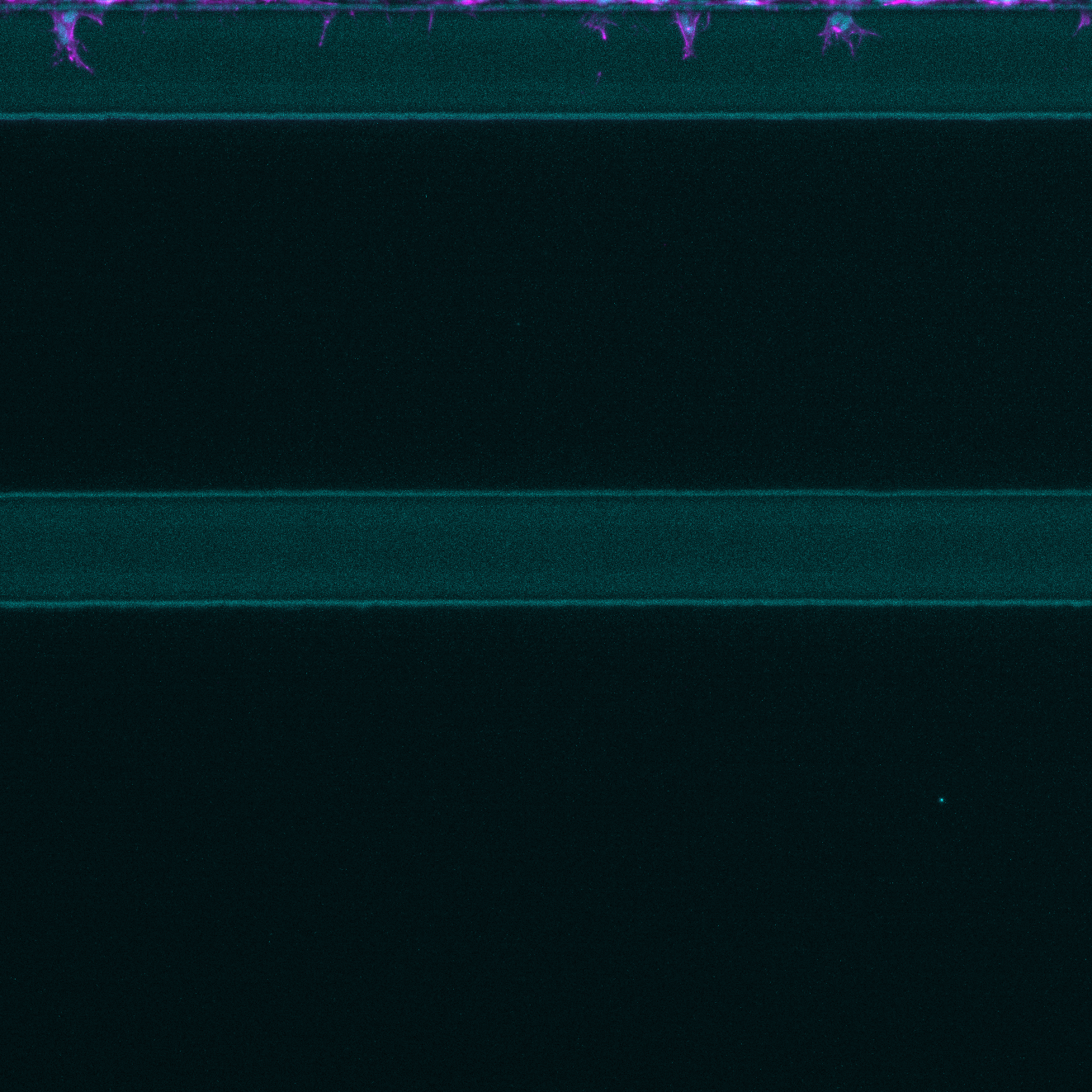

Supplement: Supplementary file 17 — Source data Fig. 6 [file 44321_2025_364_MOESM17_ESM.zip › 6B/A2.2_21d_1.5nM+7d_Washout_.tif]

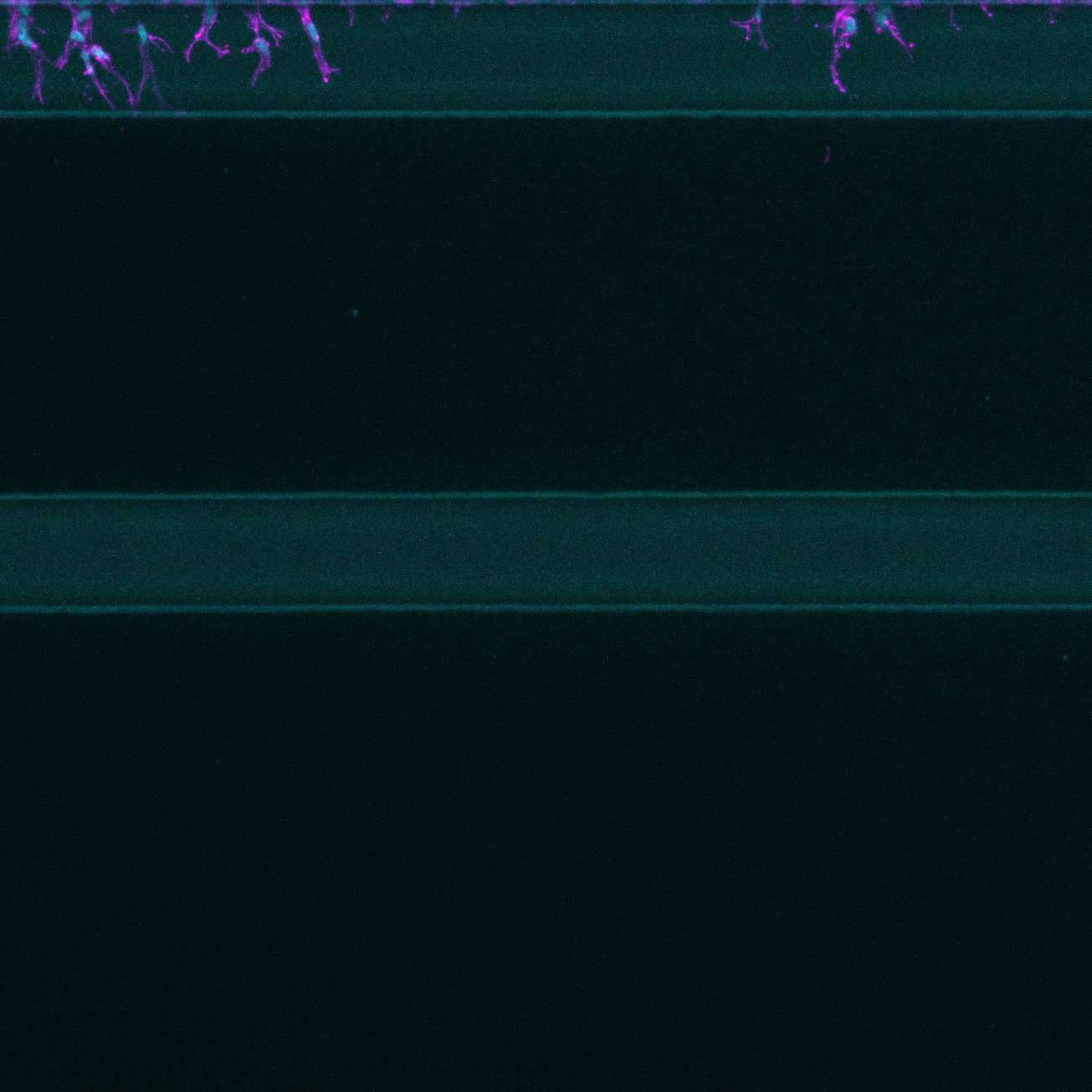

Supplement: Supplementary file 17 — Source data Fig. 6 [file 44321_2025_364_MOESM17_ESM.zip › 6B/A2.2_21d_1.5nM.tif]

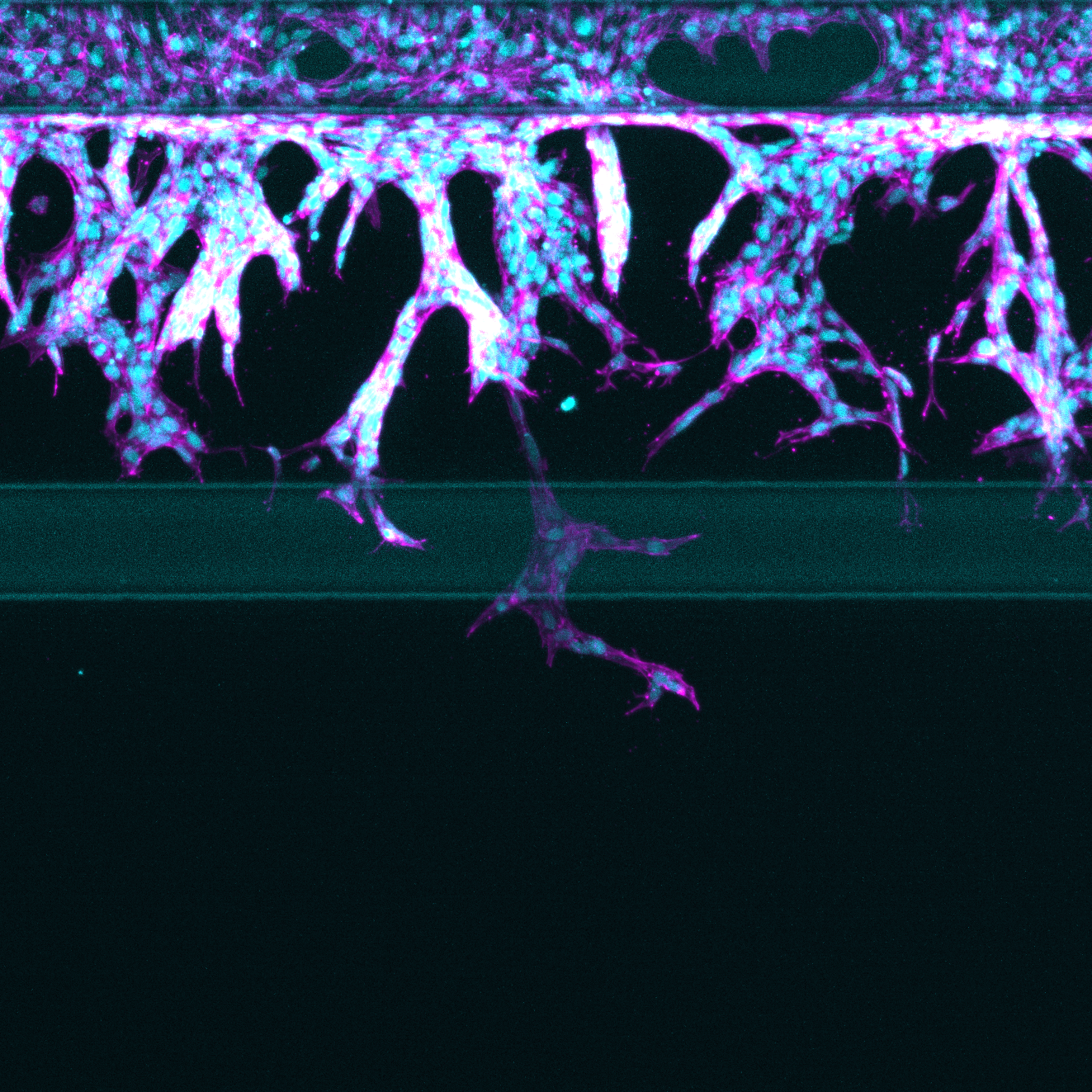

Supplement: Supplementary file 17 — Source data Fig. 6 [file 44321_2025_364_MOESM17_ESM.zip › 6B/A2.2_21d_150nM+7d_Washout_.tif]

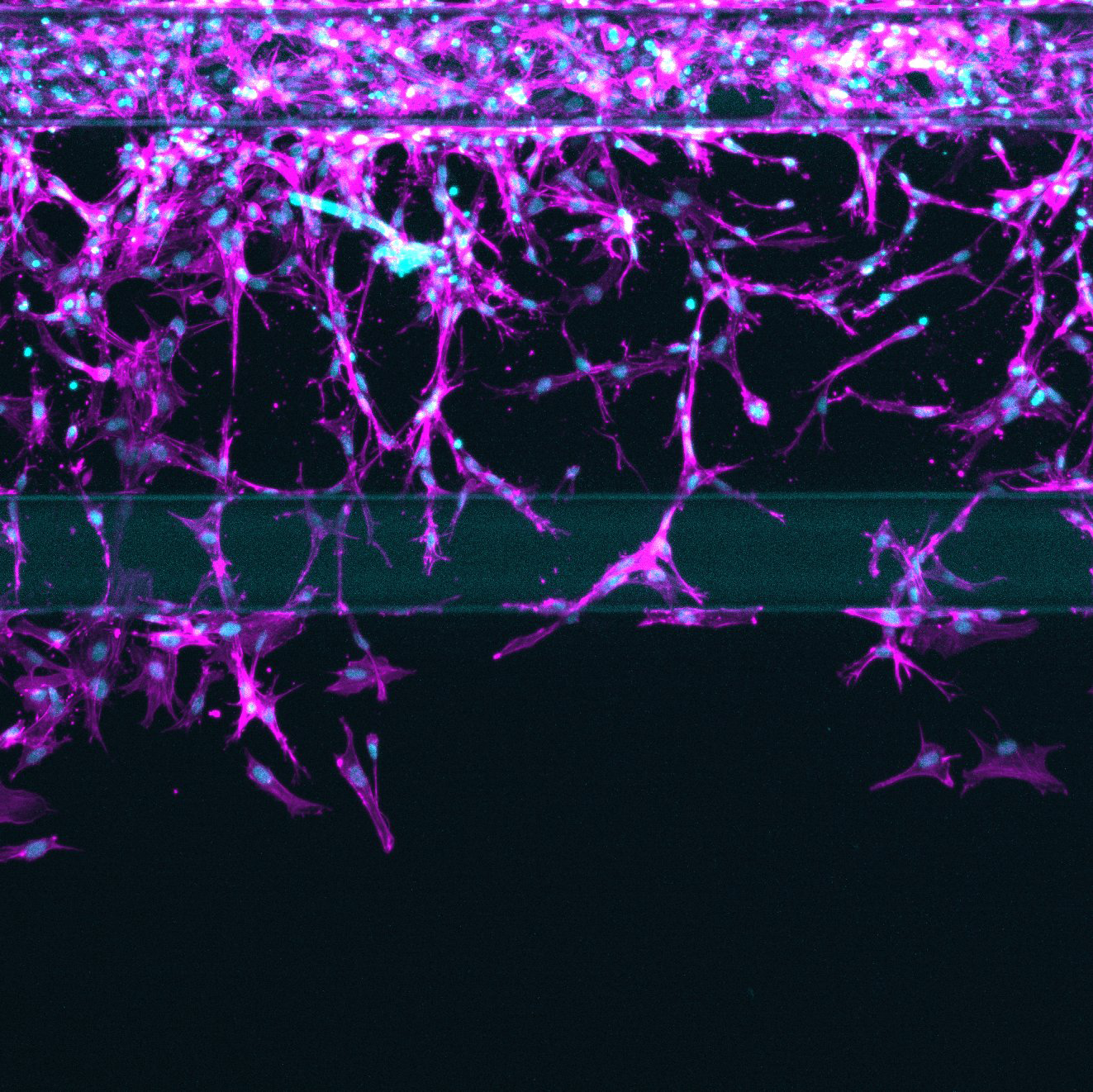

Supplement: Supplementary file 17 — Source data Fig. 6 [file 44321_2025_364_MOESM17_ESM.zip › 6B/A2.2_21d_150nM.tif]

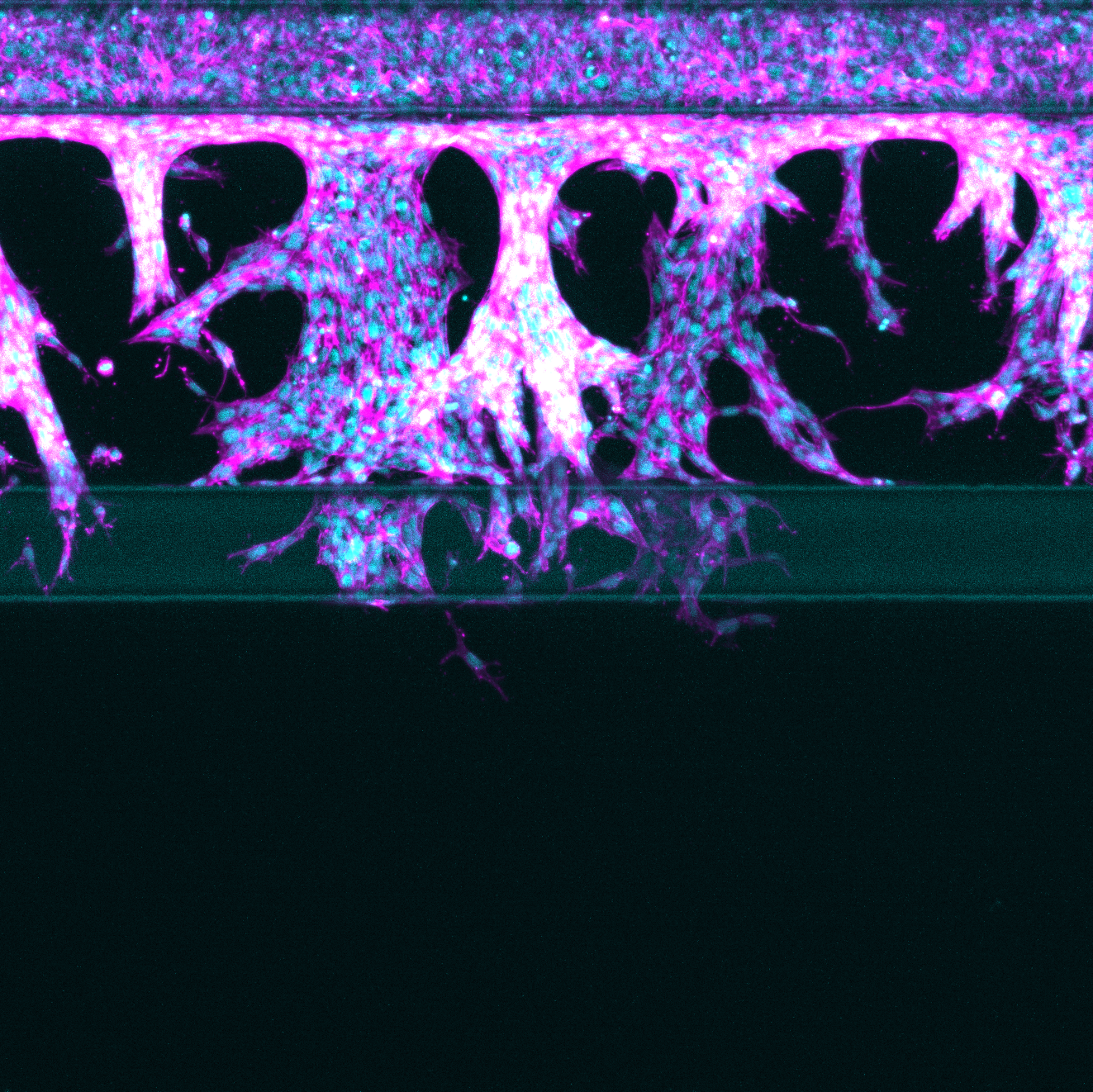

Supplement: Supplementary file 17 — Source data Fig. 6 [file 44321_2025_364_MOESM17_ESM.zip › 6B/A2.2_21d_15nM+7d_Washout_.tif]

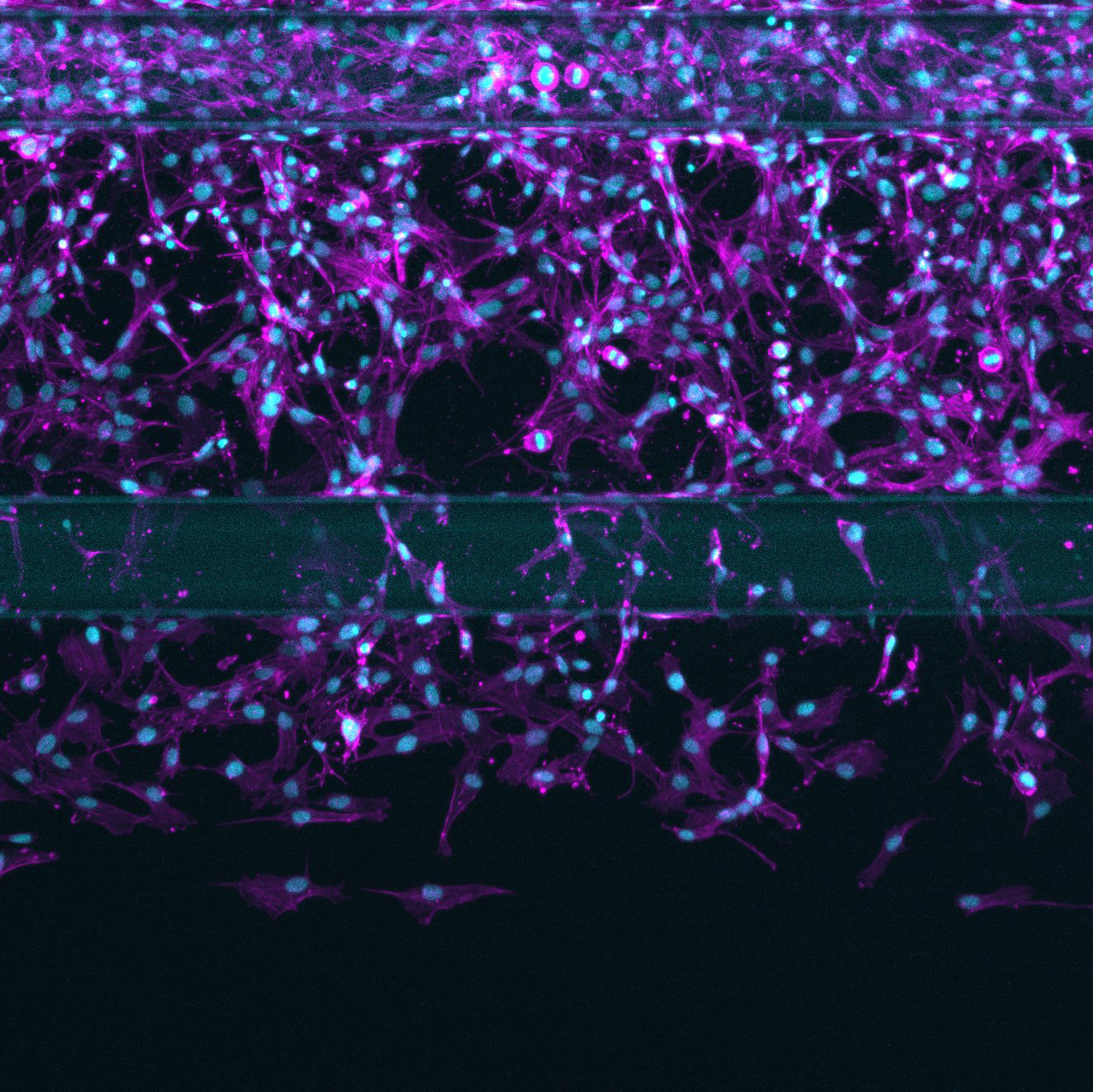

Supplement: Supplementary file 17 — Source data Fig. 6 [file 44321_2025_364_MOESM17_ESM.zip › 6B/A2.2_21d_15nM.tif]

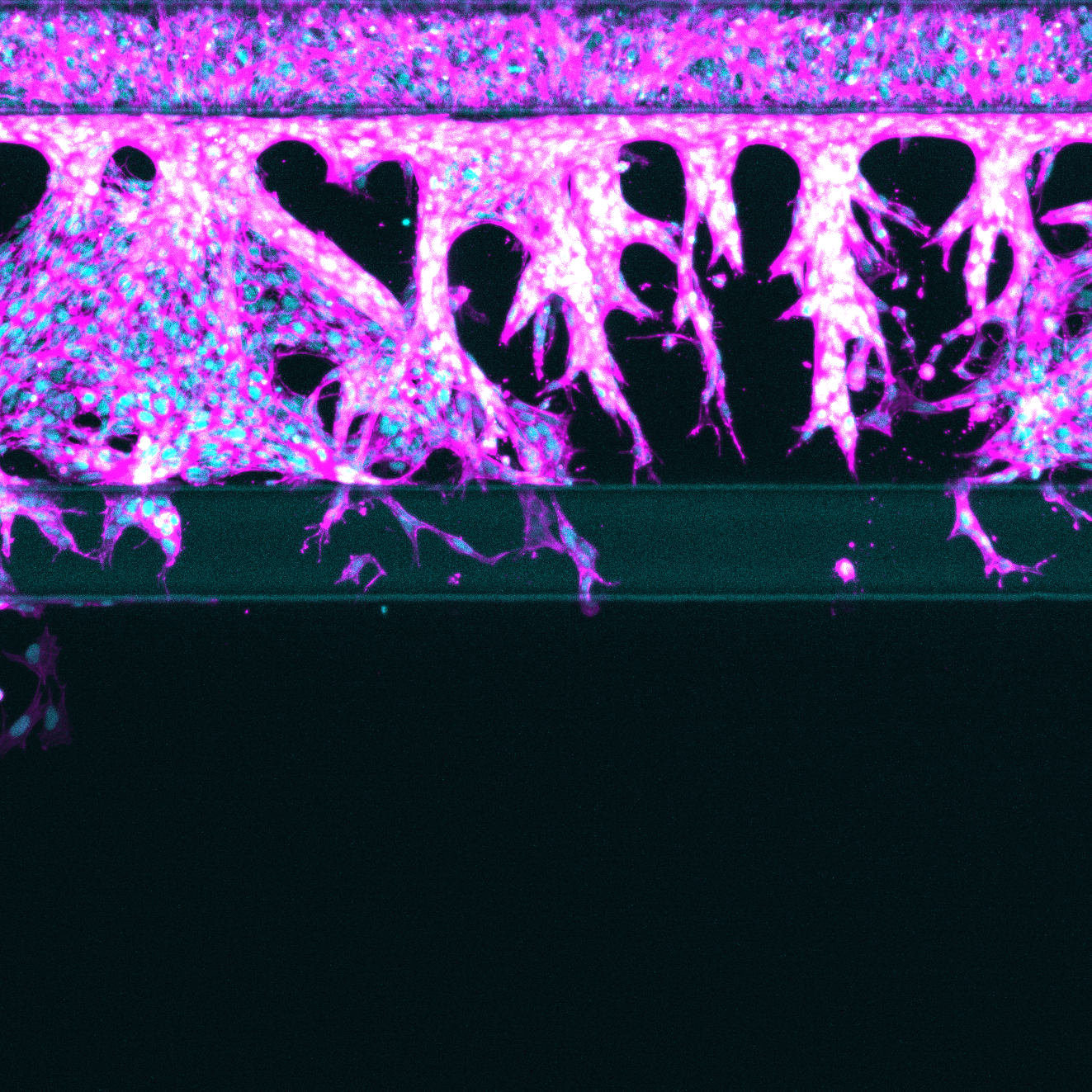

Supplement: Supplementary file 17 — Source data Fig. 6 [file 44321_2025_364_MOESM17_ESM.zip › 6B/A2.2_21d_50nM+7d_Washout_.tif]

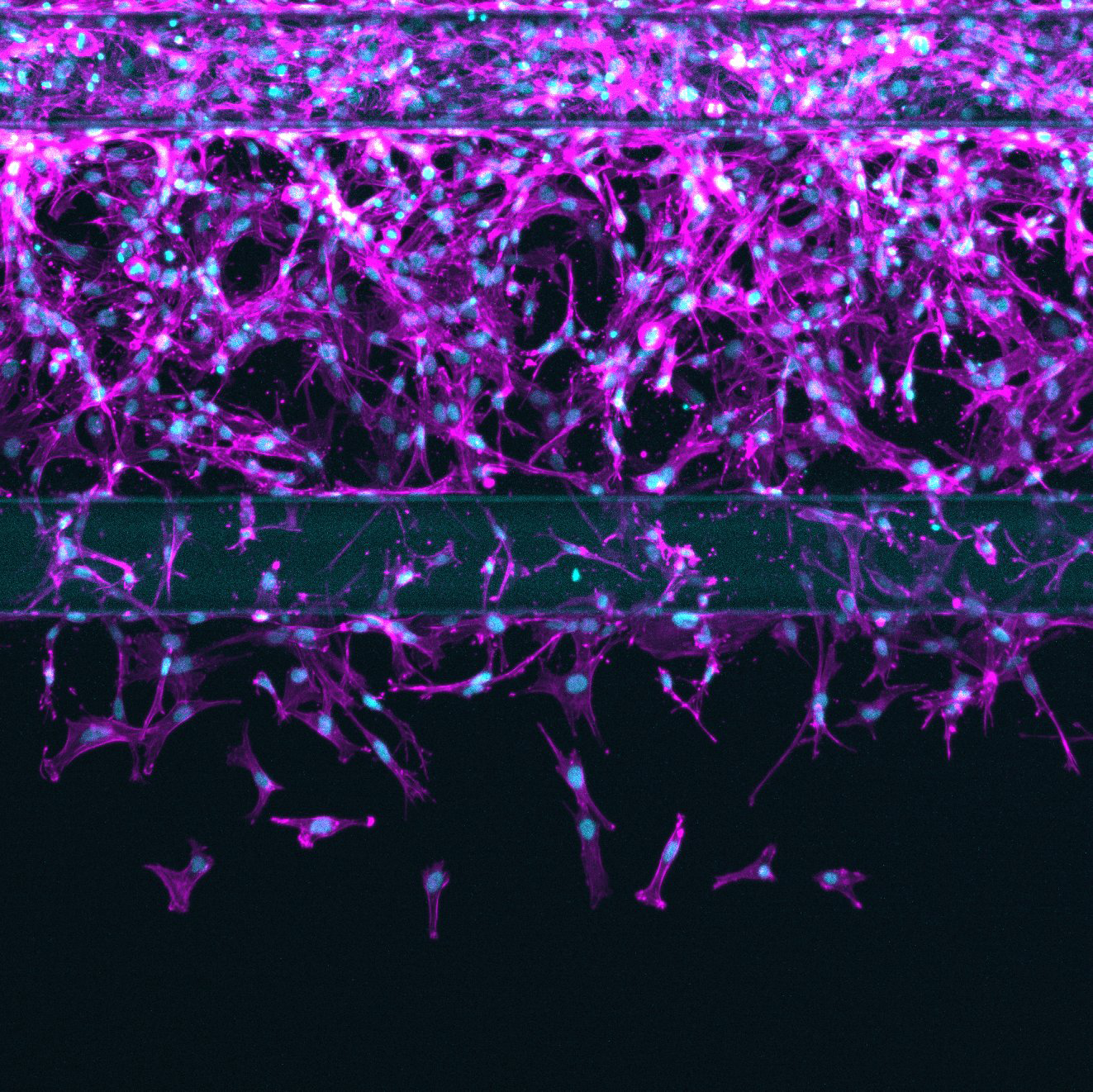

Supplement: Supplementary file 17 — Source data Fig. 6 [file 44321_2025_364_MOESM17_ESM.zip › 6B/A2.2_21d_50nM.tif]

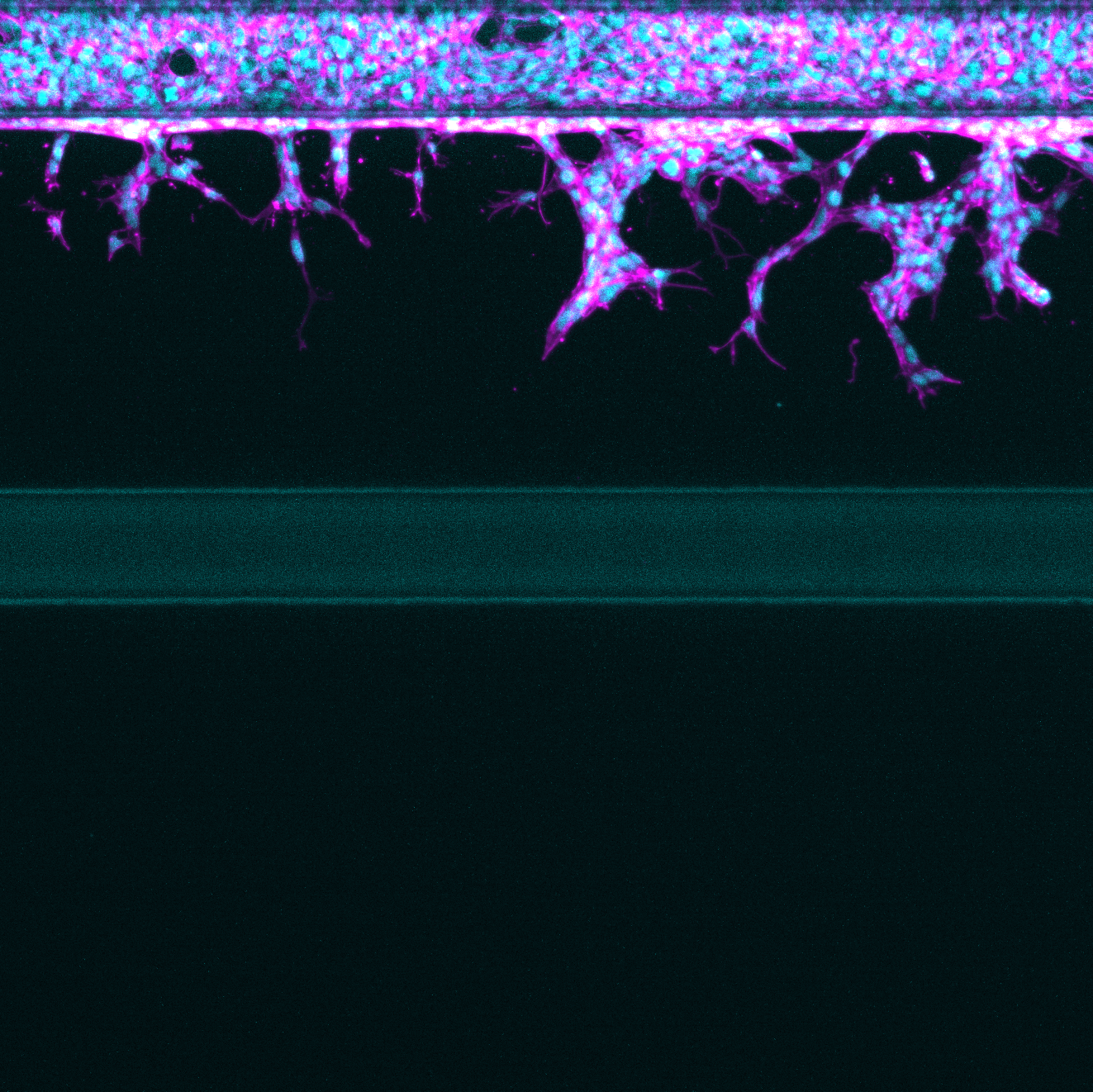

Supplement: Supplementary file 17 — Source data Fig. 6 [file 44321_2025_364_MOESM17_ESM.zip › 6B/A2.2_21d_5nM+7d_Washout_.tif]

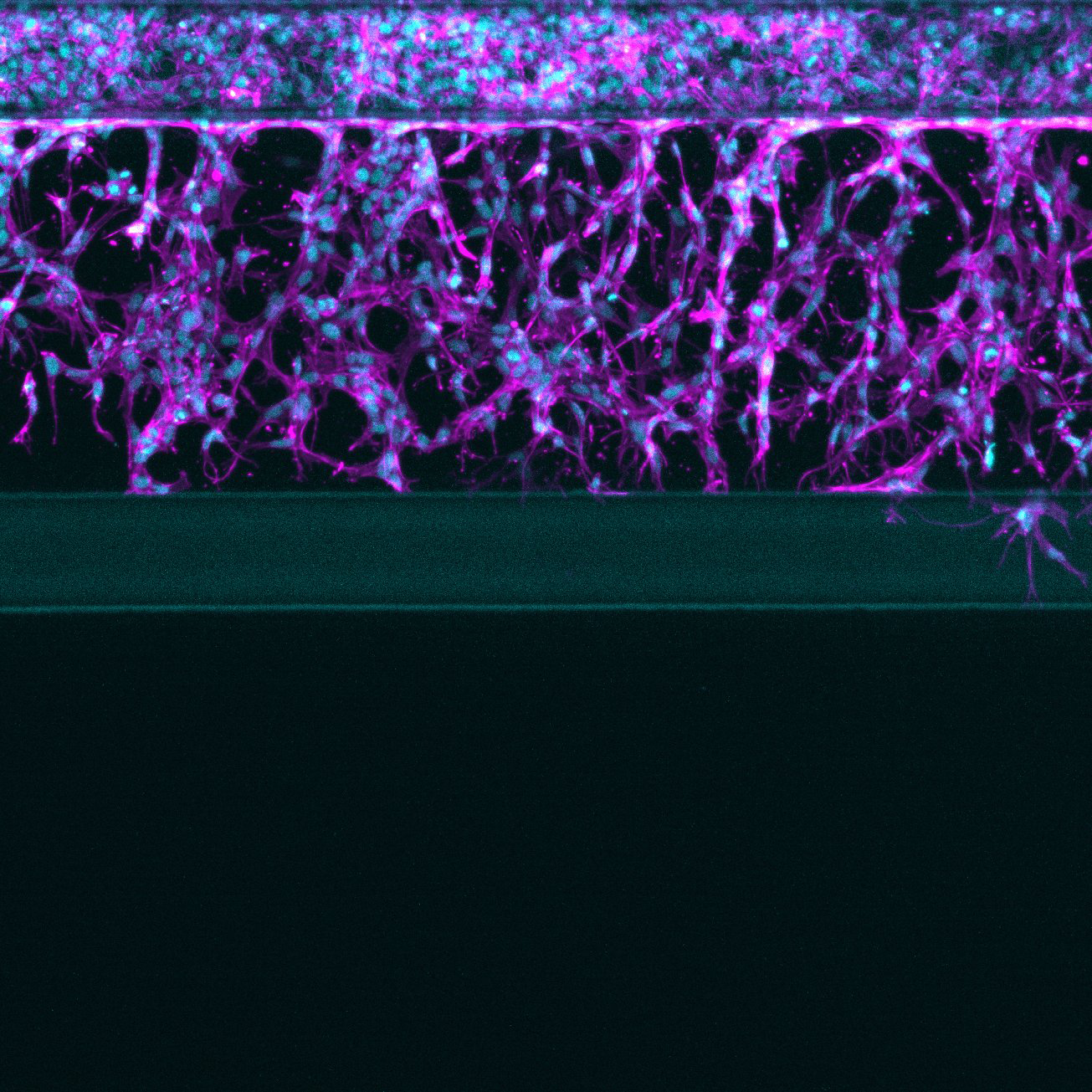

Supplement: Supplementary file 17 — Source data Fig. 6 [file 44321_2025_364_MOESM17_ESM.zip › 6B/A2.2_21d_5nM.tif]

Uncropped 6C

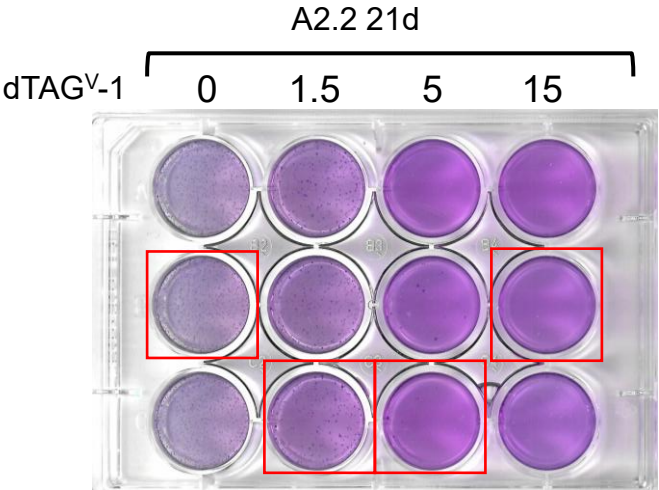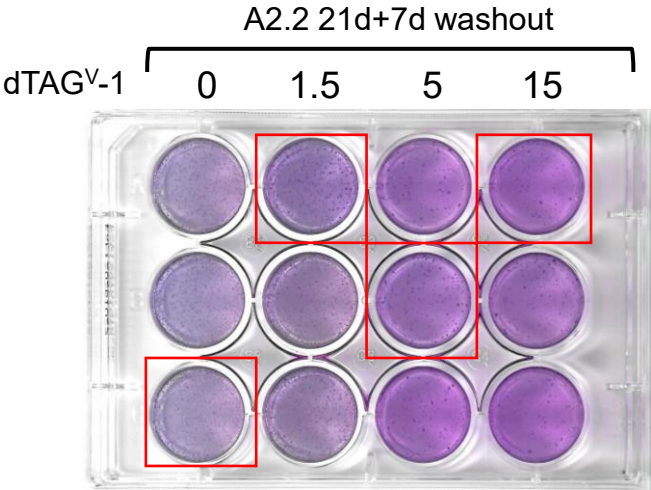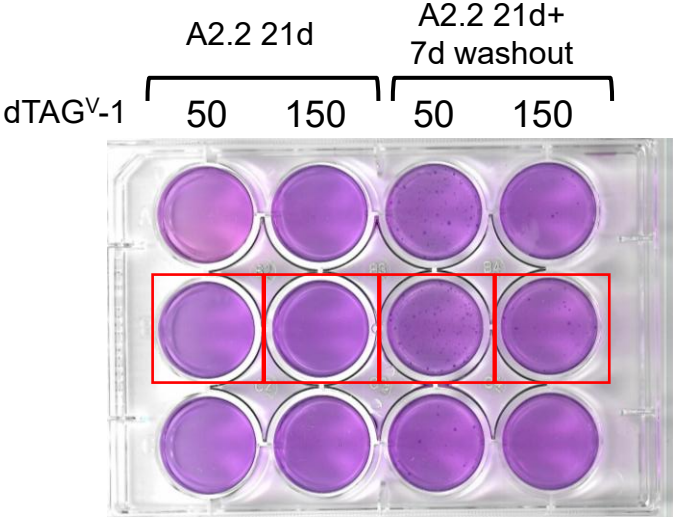

Supplement: Supplementary file 17 — Source data Fig. 6 [file 44321_2025_364_MOESM17_ESM.zip › 6C/Softagar_sourcedata.pdf]

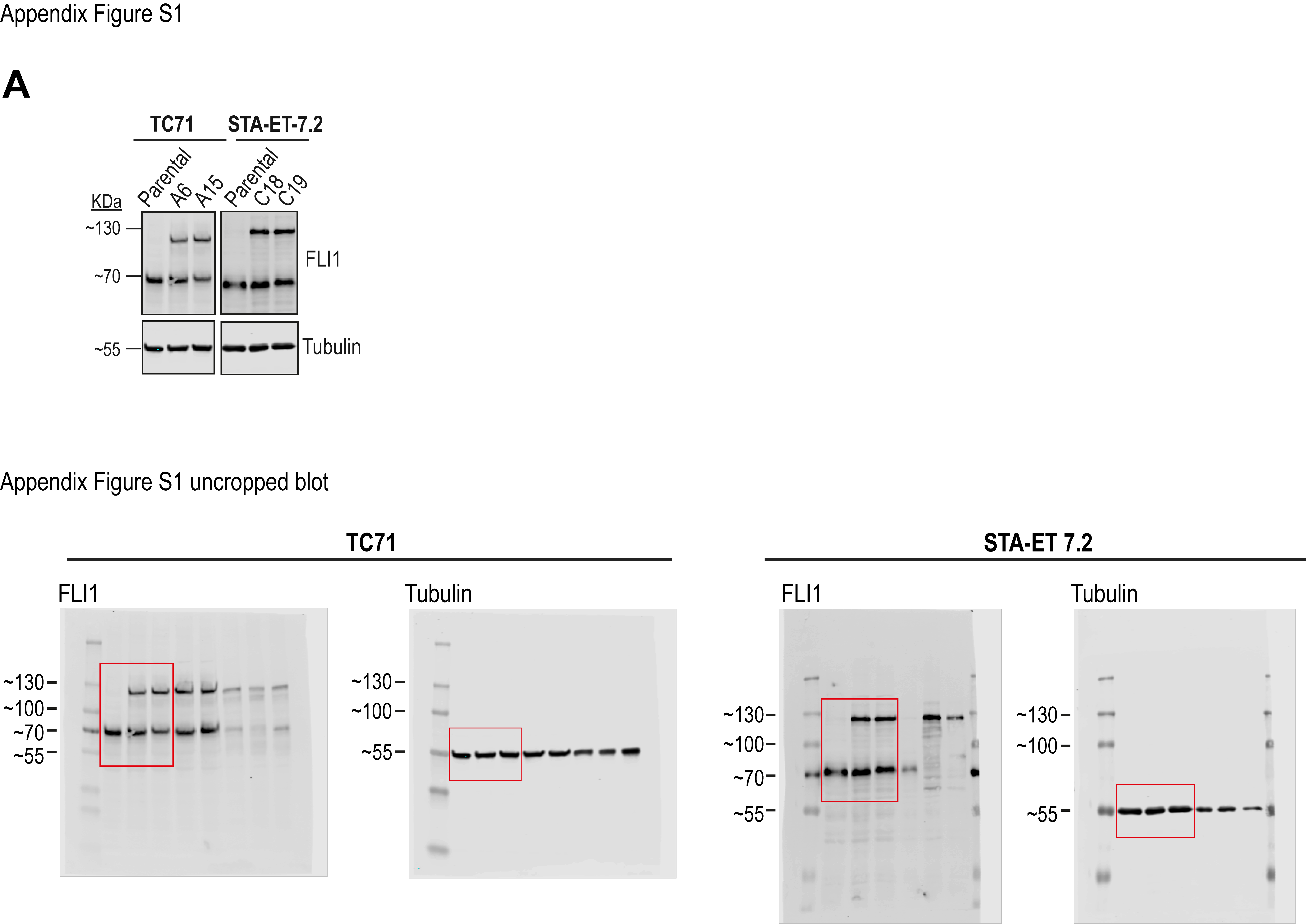

Supplement: Supplementary file 18 — Appendix Figure S1 Source Data [file 44321_2025_364_MOESM18_ESM.zip › EMM-2025-22180-V3-Figure_S1_Source_Data-sd.tif]
