# Supplementary material for: Modelling EWS::FLI1 protein fluctuations reveal determinants of tumor plasticity in Ewing sarcoma
Source: EMBO Mol Med. 2026 Jan 3;18(2):646–76. doi: 10.1038/s44321-025-00364-7 (PMC12905378; doi:10.1038/s44321-025-00364-7)
Supplement: Supplementary file 19 — Expanded View Figures [file 44321_2025_364_MOESM19_ESM.pdf]

# Expanded View Figures

**Figure EV1. Characterization of EF-dTAG clones.**

(**A**) Schematic depicting primer locations (arrows) on the FLI1-LHA-mNG-HA-FKBP12$^{F36V}$–FLI1-RHA homology-directed repair template used for clonal genotyping of the FLI1 locus (top). Agarose gel images showing products of two-step RT-PCR, confirming the presence of the tagged EWSR1::FLI1 fusion transcript in five EF-dTAG clones with biallelic knock-in, using the indicated primers (bottom). (**B**) Western blot analysis of untagged and tagged EF protein levels in parental A673 cells and EF-dTAG clones. Wild-type FLI1 (~52 kDa) was undetectable in all samples tested. Tubulin was used as a loading control. Data shown are representative of at least three independent biological replicates. (**C**) FLI1 CUT&RUN-seq peak signal in A2.2 dTAG clone and A673 parental line. Each dot is one of 12,370 consensus peaks. Values indicate normalized read counts, averaged across replicates. (**D**) Number of differentially expressed genes (DEGs) between single-cell clones and A673 parental line (left, DESeq2, FDR <0.05, absolute $\log_2$ fold change >1). Functional enrichment of DEGs identifies MSigDB Hallmark 2020 and KEGG 2021 pathways as significantly enriched at least once (right, hypergeometric test, FDR <0.001, $\log_2$ odds ratio >3, background: all DESeq2 tested genes). (**E**) Proliferation of EF-dTAG clones and parental A673 cells measured using a CellTiter-Glo assay. Data were presented as mean ± SEM ($n = 3$ independent experiments). (**F**) Transwell migration assays showing migrated cells stained with crystal violet 24 h after seeding, representative images from one of the three biological replicates (left). Migrated cell counts are presented as mean ± SEM after log10 transformation (right; $n = 3$), scale bar = 200 µm. (**G**) Organoplate invasion assays depicting cell invasion into collagen I extracellular matrix (ECM) 7 days after seeding EF-dTAG clones and parental A673. Cells were pretreated with 150 nM dTAG$^{V}$-1 for 24 h where indicated. Cells were stained with Hoechst (cyan, nuclei) and phalloidin (magenta, actin cytoskeleton) (left). Representative images from one of three independent biological replicates are shown, scale bars = 100 µm. Nuclei positions along the Y-axis in the organoplate invasion assays are depicted, with phase guides shown as dotted lines at distances of 535 and 930 µm (right; $n = 3$). (**H**) Soft agar colony formation assays showing crystal violet-stained colonies formed by EF-dTAG clones and parental A673 cells 3 weeks after seeding. Representative images from one of three independent biological replicates are shown (left). Colony counts are presented as mean ± SEM (right, $n = 3$) following log10 + 1 transformation. For panels (**E–H**), *P* values were calculated using one-way ANOVA with Dunnett's post hoc multiple comparisons test. ns not significant.

© The Author(s)

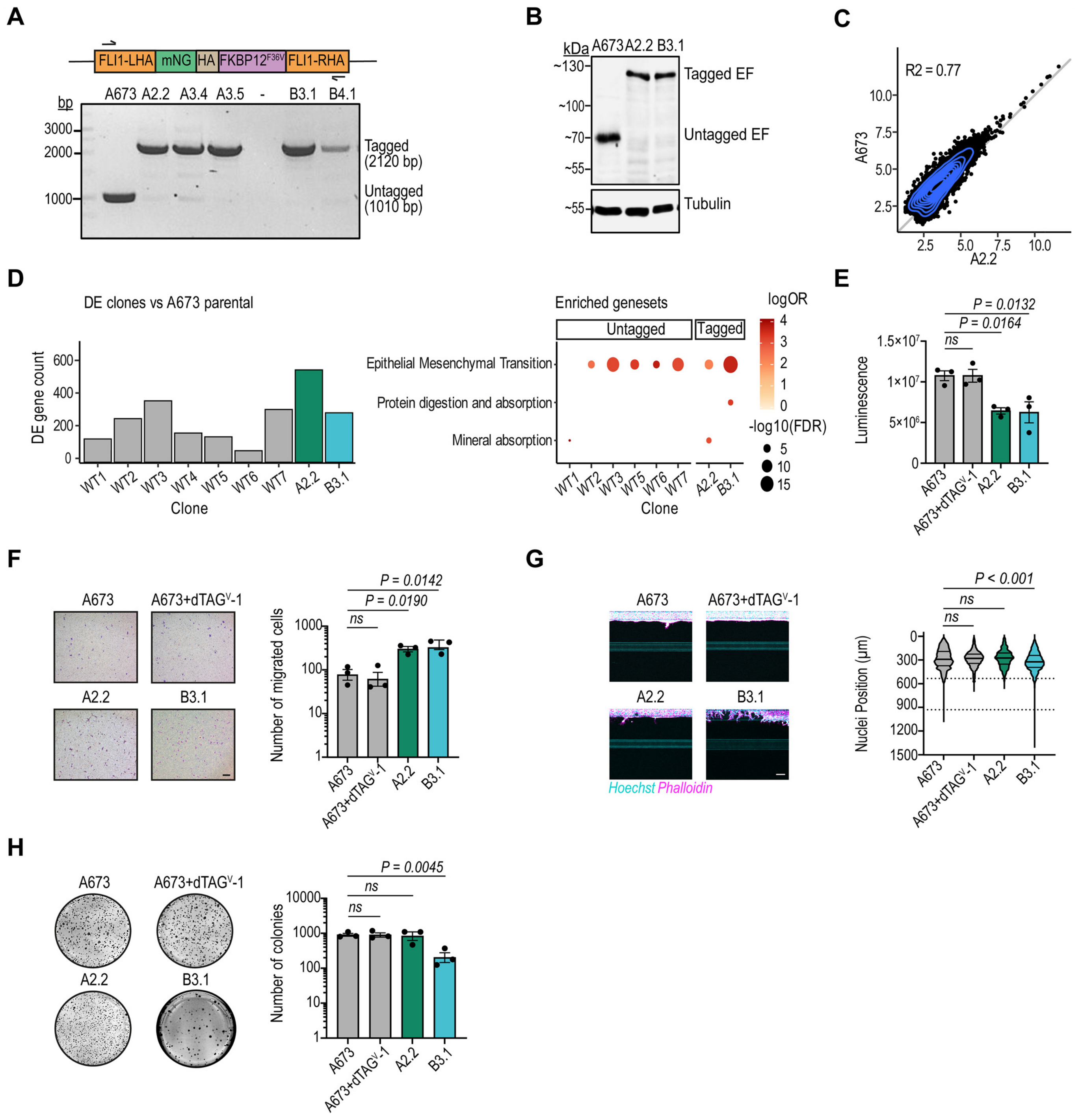
A
FLI1-LHA
mNG
HA
FKBP12F36V
FLI1-RHA
bp
A673 A2.2 A3.4 A3.5 - B3.1 B4.1
3000
2000
1000
Tagged (2120 bp)
Untagged (1010 bp)
B
kDa
A673 A2.2 B3.1
~130
~100
~70
~55
Tagged EF
Untagged EF
Tubulin
C
R2 = 0.77
A673
A2.2
D
DE clones vs A673 parental
DE gene count
Clone
WT1 WT2 WT3 WT4 WT5 WT6 WT7 A2.2 B3.1
Enriched genesets
Untagged
Tagged
Epithelial Mesenchymal Transition
Protein digestion and absorption
Mineral absorption
logOR
-log10(FDR)
E
P = 0.0132
P = 0.0164
ns
Luminescence
A673 A673+dTAGV-1 A2.2 B3.1
F
A673
A673+dTAGV-1
A2.2
B3.1
P = 0.0142
P = 0.0190
ns
Number of migrated cells
G
A673
A673+dTAGV-1
A2.2
B3.1
Hoechst Phalloidin
P < 0.001
ns
ns
Nuclei Position (μm)
H
A673
A673+dTAGV-1
A2.2
B3.1
P = 0.0045
ns
ns
Number of colonies

 © The Author(s)

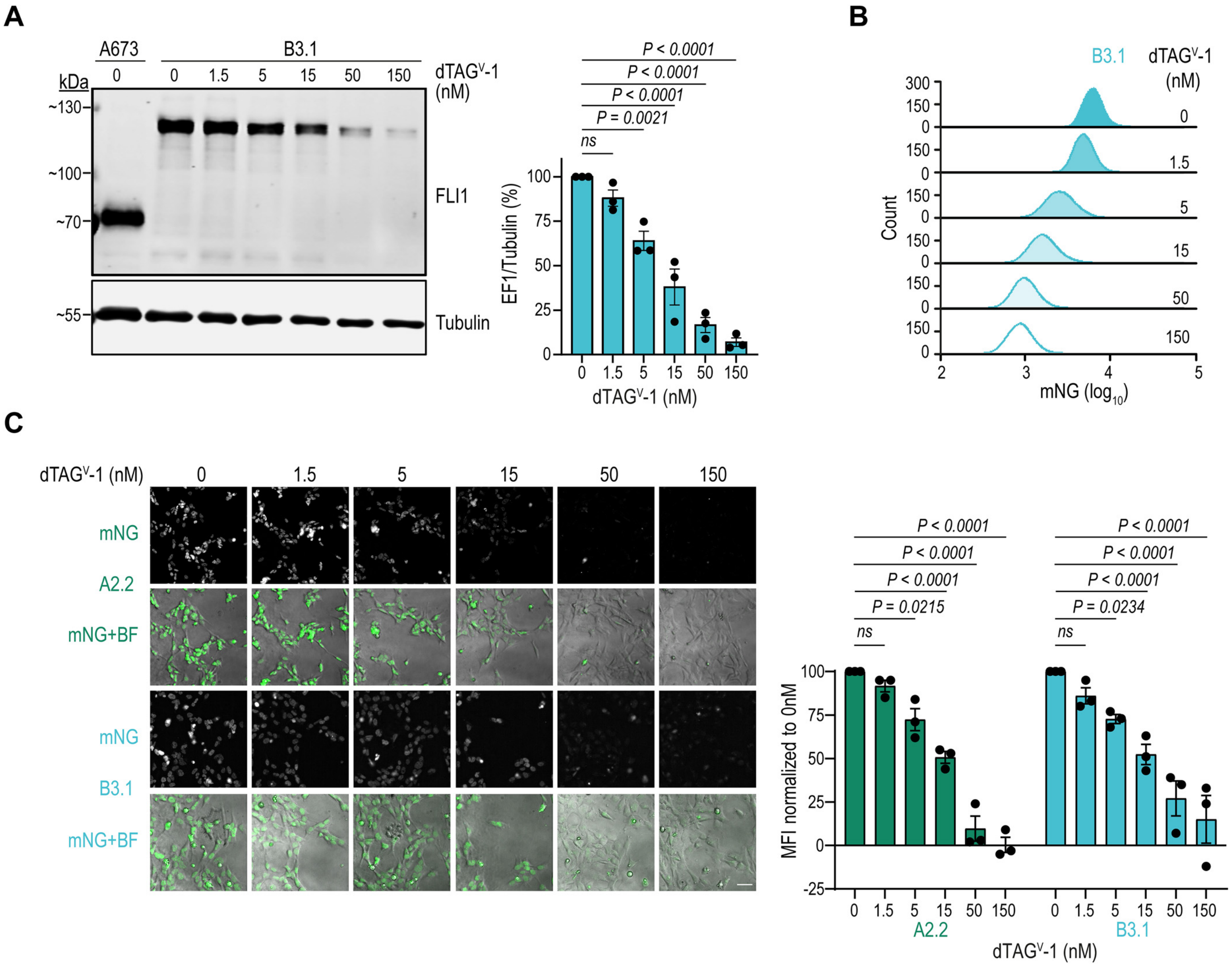


**Figure EV2. EF gradient characterization of EF-dTAG clones.**

(**A**) Western blot analysis of FLI1 protein levels in EF-dTAG clone B3.1 and parental A673 cells treated with the indicated concentrations of dTAG$^{V}$-1 for 24 h. Tubulin was used as a loading control. Bar graphs depict EF protein levels normalized to Tubulin, presented as mean ± SEM (right; $n = 3$ independent biological replicates). $P$ values were calculated using One-way ANOVA with Dunnett's post hoc multiple comparisons. (**B**) Flow cytometry analysis of mNG fluorescence intensity in EF-dTAG clone B3.1 following treatment with increasing concentrations of dTAG$^{V}$-1 for 24 h, data were representative of at least three independent biological replicates. (**C**) Confocal images for mNG fluorescence in EF-dTAG clone A2.2 (top) and B3.1 (bottom) treated with the indicated concentrations of dTAG$^{V}$-1 for 24 h, scale bars = 50 µm. Images are representative of at least three individual experiments; BF brightfield. Bar graphs represent mean mNG mean fluorescence intensity values normalized to the 0 nM control, presented as mean ± SEM (right; $n = 3$ independent experiments). $P$ values were calculated using two-way ANOVA with Dunnett's post hoc multiple comparisons test. ns not significant.

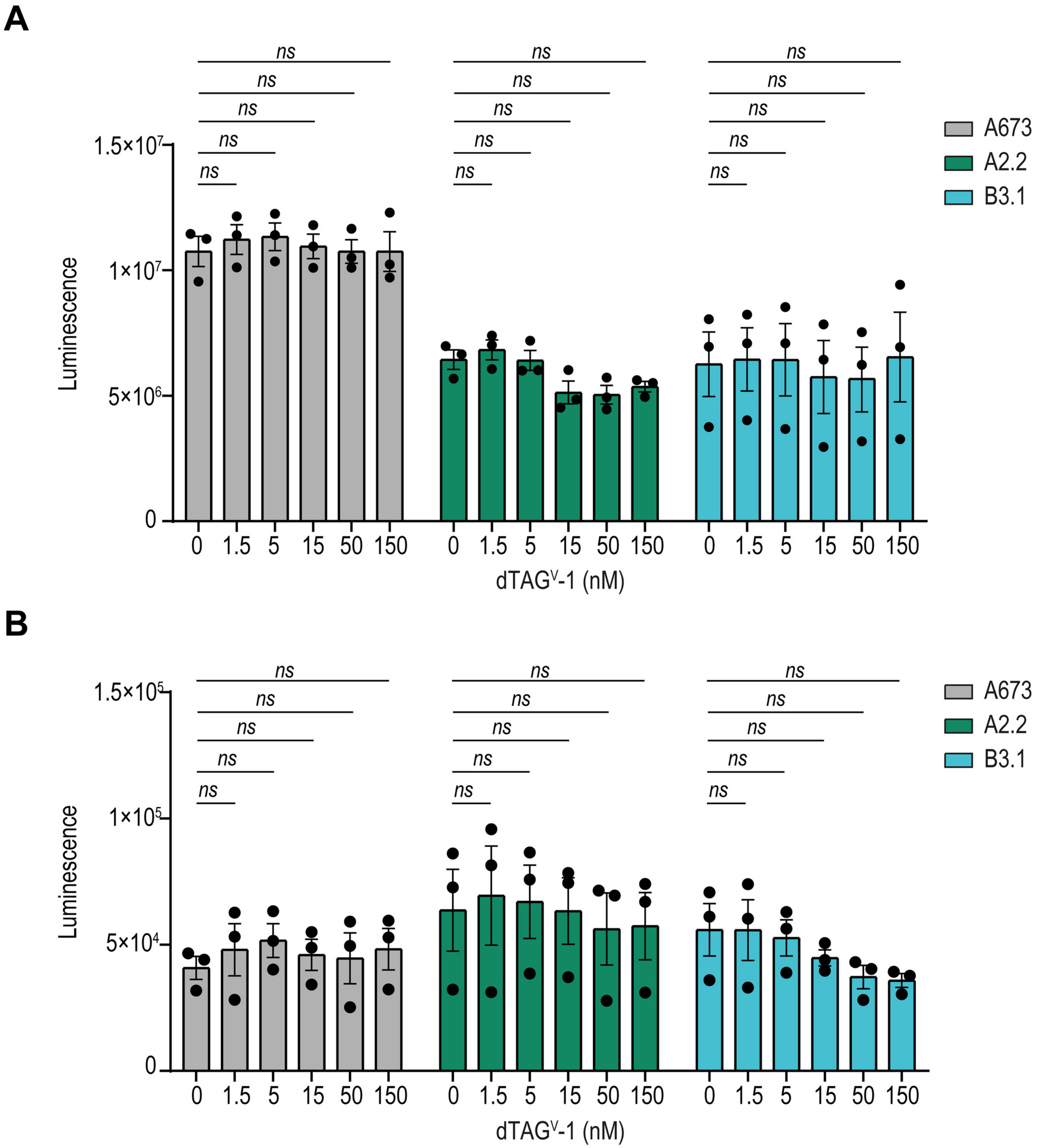


**Figure EV3. Effects of dTAG$^{V}$-1 on proliferation and apoptosis.**

(**A**) Proliferation of EF-dTAG clones and parental A673 cells in response to increasing concentrations of dTAG$^{V}$-1, assessed using the CellTiter-Glo assay. Data represents the mean ± SEM of $n = 3$ independent biological replicates. (**B**) Apoptosis of EF-dTAG clones and parental A673 cells in response to increasing concentrations of dTAG$^{V}$-1, measured using the Caspase-Glo 3/7 assay. Data represents the mean ± SEM of $n = 3$ independent biological replicates. *P* values for both panels were determined using two-way ANOVA with Dunnett's multiple comparisons test. ns not significant.

 © The Author(s)

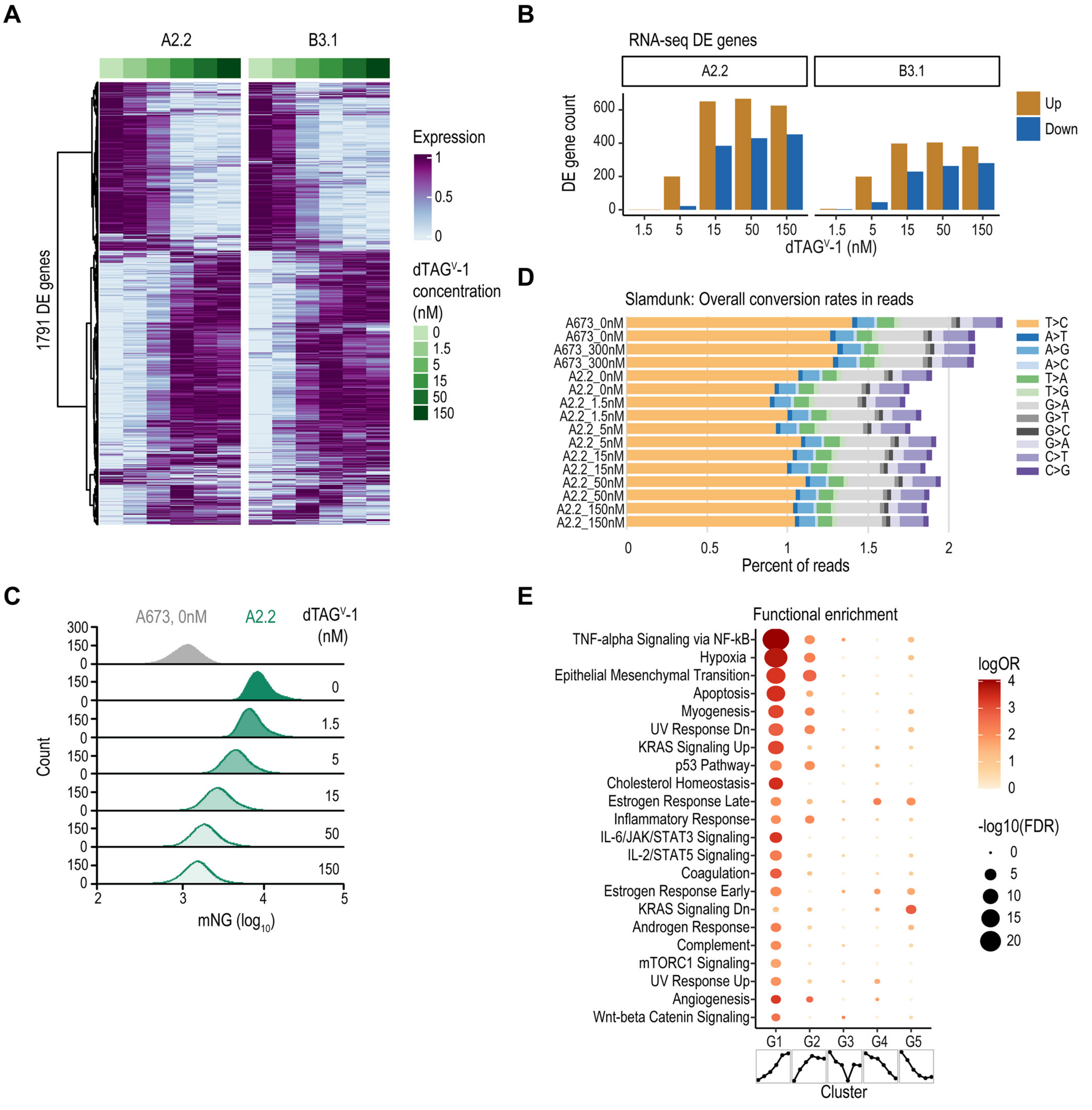


**Figure EV4. Transcription profiling of EF-dTAG clones in response to dTAG$^{V}$-1 treatment.**

(**A**) Heatmaps showing gene expression changes in the 24-hour dTAG$^{V}$-1 gradient treatment experiment. The Y-axis shows all 1791 genes determined to be differentially expressed (DESeq2, FDR <0.05, absolute $log_2$ fold change >2) in at least one EF-dTAG clone at any dTAG$^{V}$-1 concentration. Gene expression was normalized using DESeq2 vst, averaged across replicates, and rescaled per gene per clone. (**B**) Number of DEGs from panel (**A**) across treatment conditions. (**C**) Flow cytometry analysis of mNG fluorescence intensity in EF-dTAG clone A2.2 after 3 h of treatment with increasing dTAG$^{V}$-1 concentrations, representative of samples sequenced in SLAM-seq. (**D**) SLAM-seq base conversion rates in percent of all reads as reported by the Slamdunk processing pipeline. (**E**) Functional enrichment analysis of SLAM-seq cluster genes, showing MSigDB Hallmark 2020 pathways that were significantly enriched (hypergeometric test, FDR <0.05, $log_2$ odds ratio >1, background: all detected genes) in at least one cluster. Icons below the cluster labels show the mean expression pattern per cluster (x-axis shows increasing dTAG$^{V}$-1 concentrations, y-axis rescaled expression).

© The Author(s)

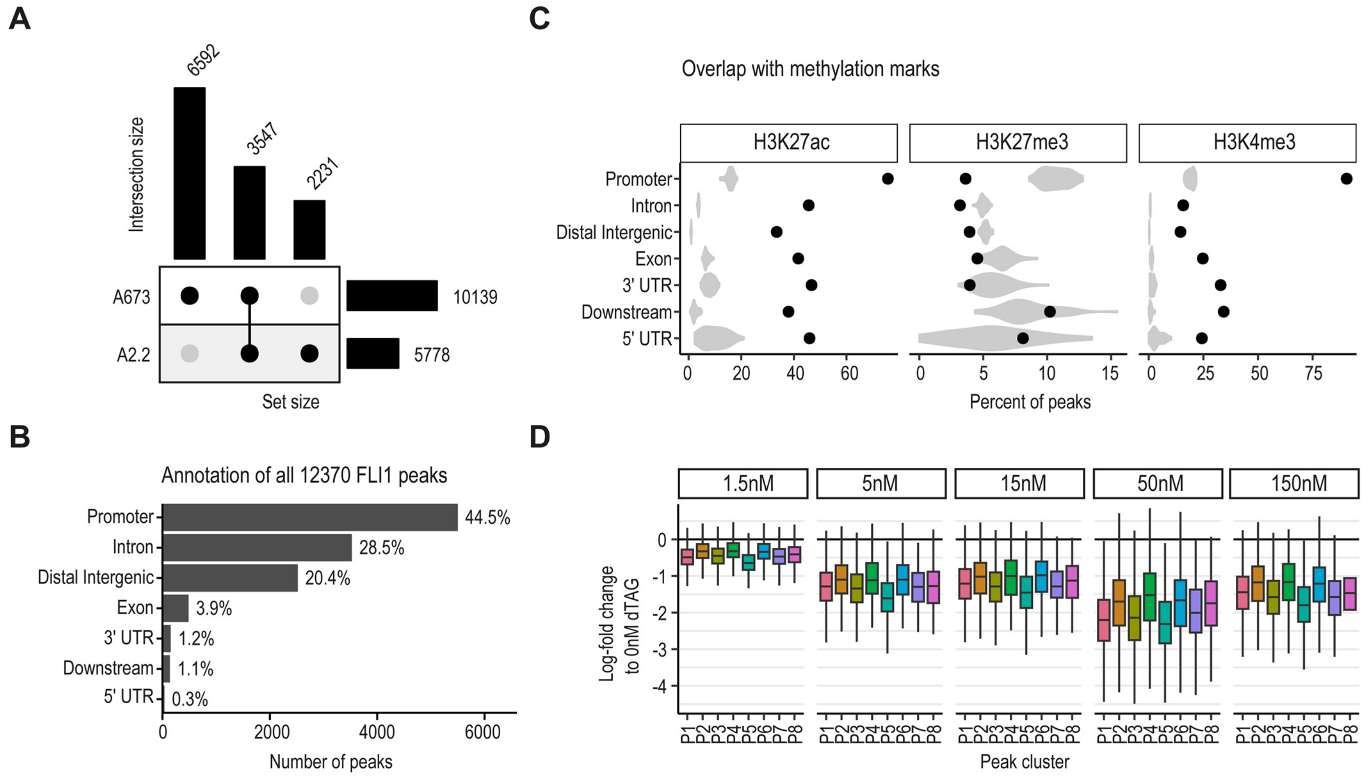


**Figure EV5. Characterization of FLI1-binding peaks and dynamics of peak signals.**

(**A**) UpSet plot showing the intersection of the CUT&RUN FLI1 peaks discovered in A2.2 dTAG clone and parental A673 cells. (**B**) Annotation of FLI1 peaks with respect to genomic location (Promoter = 5501, Intron = 3527, Distal intergenic = 2520, Exon = 485, 3′UTR = 152, Downstream = 137, 5′UTR = 37). (**C**) Percentage of FLI1 peaks (x-axis), grouped by genomic location (y-axis), overlapping methylation marks. Observed percentages are shown as black points and random background distributions of shuffled peaks (all peaks randomly placed in the genome 50 times) depicted as gray violin plots. (**D**) FLI1 CUT&RUN peak signal change for all FLI1 consensus peaks grouped by peak cluster and dTAG$^{V}$-1 concentration in A2.2 clone. Boxplots show log-fold change compared to 0 nM condition as reported by DESeq2 with ashr shrinkage applied (P1 = 4160, P2 = 2409, P3 = 2079, P4 = 1367, P5 = 958, P6 = 661, P7 = 538, P8 = 198). Boxplots show 25% (lower hinge), 50% (horizontal line), and 75% (upper hinge) quantiles. The whiskers extend from the lower/upper hinge to the smallest/largest value within 1.5 times the hinge spread. Outliers are not shown.


© The Author(s)

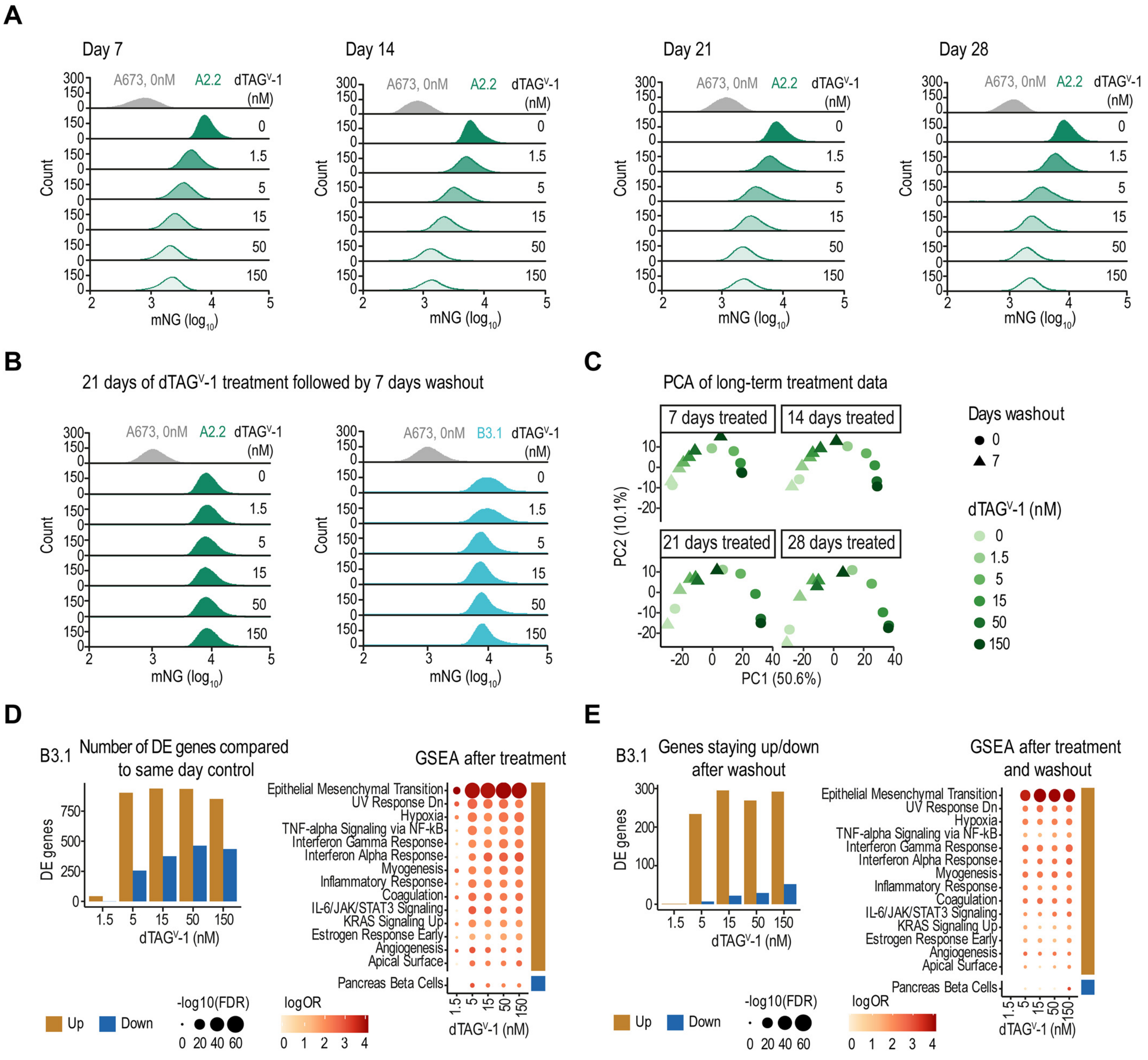


**Figure EV6. Long-term EF gradient treatment and transient effects due to dTAG$^{V}$-1 treatment.**

(**A**) Flow cytometry analysis of mNG fluorescence intensity in EF-dTAG clone A2.2 after 7, 14, 21, and 28 days of treatment with increasing concentrations of dTAG$^{V}$-1. Representative data corresponds to one of the technical replicates used for RNA-sequencing. (**B**) Flow cytometry analysis of mNG fluorescence intensity in EF-dTAG clones after 21 days of dTAG$^{V}$-1 treatment at the indicated concentrations, followed by a 7-day washout period. Representative data corresponds to one of the technical replicates used for RNA-sequencing. (**C**) PCA of prolonged and transient EF dosage modulation on transcription in A2.2 clone (RNA-seq), showing the first two principal components calculated using 1455 highly variable genes (gene expression with standard deviation >0.5 after normalization with DESeq2 vst function and averaging two replicates). (**D**) Number of DEGs (Left, DESeq2, FDR <0.05, absolute $log_2$ fold change >2) and functional pathways enrichment analysis (Right) for clone B3.1 after 21-day treatment with increasing concentrations of dTAG$^{V}$-1. Pathways shown are MSigDB Hallmark 2020 pathways that were significantly enriched (hypergeometric test, FDR <0.001, $log_2$ odds ratio >3) in any treatment condition. (**E**) As (**D**) but showing results for treatment followed by a 7-day washout.

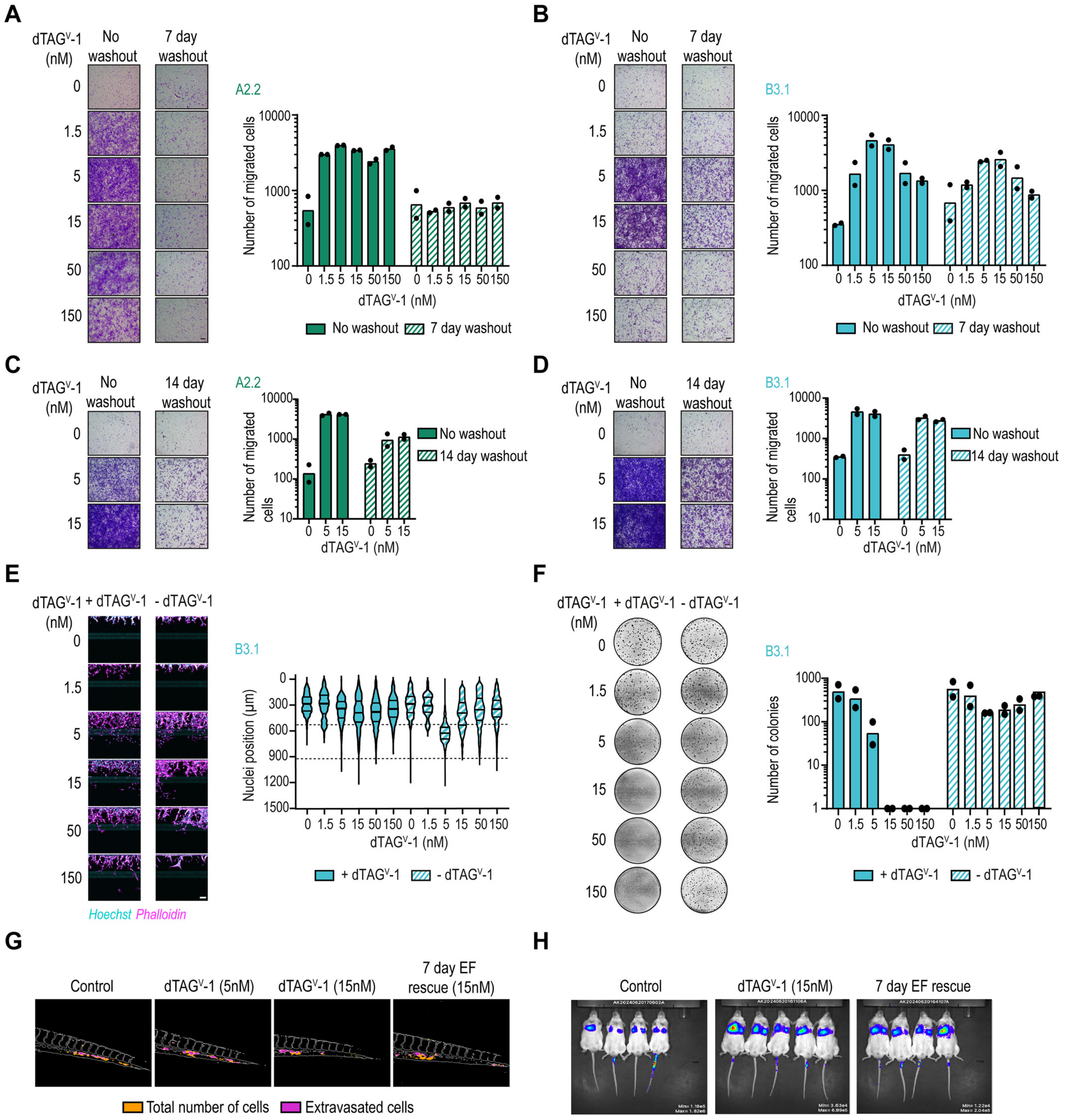
A
B
C
D
E
F
G
H
A2.2
B3.1
No washout
7 day washout
14 day washout
Number of migrated cells
Nuclei position (μm)
Number of colonies
+ dTAG^V-1
- dTAG^V-1
Hoechst Phalloidin
Control
dTAG^V-1 (5nM)
dTAG^V-1 (15nM)
7 day EF rescue (15nM)
7 day EF rescue
Total number of cells
Extravasated cells

 © The Author(s)

**Figure EV7. Metastatic phenotypic analysis of EF-dTAG clones following 21-day dTAG$^V$-1 treatment and washout.**

(**A**) Transwell migration assays showing migrated A2.2 cells after 7-day treatment with dTAG$^V$-1 at the indicated concentrations or treatment for 7 days followed by a 7-day washout. Representative images from one of two independent biological replicates are shown (left). Migrated cell counts are presented as mean after log10 transformation (right; $n = 2$). Scale bar = 200 µm. (**B**) Transwell migration assays showing migrated B3.1 cells following treatment with dTAG$^V$-1 for 21 days at the indicated concentrations, or treatment for 21 days followed by a 7-day washout. Representative images from one of two independent biological replicates are shown (left). Migrated cell counts, presented as mean after log10 transformation, are shown on the right ($n = 2$). Scale bar = 200 µm. (**C**, **D**) Transwell migration assays showing migrated A2.2 cells (**C**) or B3.1 cells (**D**) following 21-day treatment with dTAG$^V$-1 at the indicated concentrations as in Fig. 6A (A2.2 no washout) and EV7B (B3.1 no washout) or following 21 days of treatment and a subsequent 14-day washout. Representative images from one of two independent biological replicates are shown (left). Migrated cell counts are presented as mean after log10 transformation (right; $n = 2$). Scale bar = 200 µm. (**E**) Organoplate invasion assays showing cell invasion into collagen I ECM 7 days after seeding B3.1 cells treated with dTAG$^V$-1 for 21 days at the indicated concentrations, with or without further dTAG$^V$-1 treatment. Representative images from one of two independent biological replicates are shown (left). Mean nuclei positions along the Y-axis are shown, with phase guides indicated by dotted lines at 535 and 930 µm (right; $n = 2$). Scale bar = 100 µm. (**F**) Soft agar assays showing colonies formed by B3.1 cells three weeks after seeding 21 days dTAG$^V$-1 treated cells at indicated concentrations with or without further dTAG$^V$-1 treatment. Representative images from one of two independent biological replicates are shown (left). Colony counts are presented as mean ($n = 2$) following log10 + 1 transformation. (**G**) Representative images of zebrafish extravasation assays 3 days post injection of cells treated with dTAG$^V$-1 for 21 days at the indicated concentrations, or 21 days of 15 nM dTAG$^V$-1 followed by a 7-day washout. (**H**) Representative IVIS images of mice showing the homing of luciferase-expressing A2.2 cells to lungs directly after (Day 0) tail vein injection of cells treated with 15 nM dTAG$^V$-1 for 21 days, with or without a 7-day washout, along with a control. *P* values were calculated using two-way ANOVA with Dunnett's post hoc multiple comparisons test. ns not significant.

© The Author(s)
