# Supplementary material for: Modelling EWS::FLI1 protein fluctuations reveal determinants of tumor plasticity in Ewing sarcoma
Source: EMBO Mol Med. 2026 Jan 3;18(2):646–76. doi: 10.1038/s44321-025-00364-7 (PMC12905378; doi:10.1038/s44321-025-00364-7)

Uncropped 6E- Matched lungs and livers at endpoint

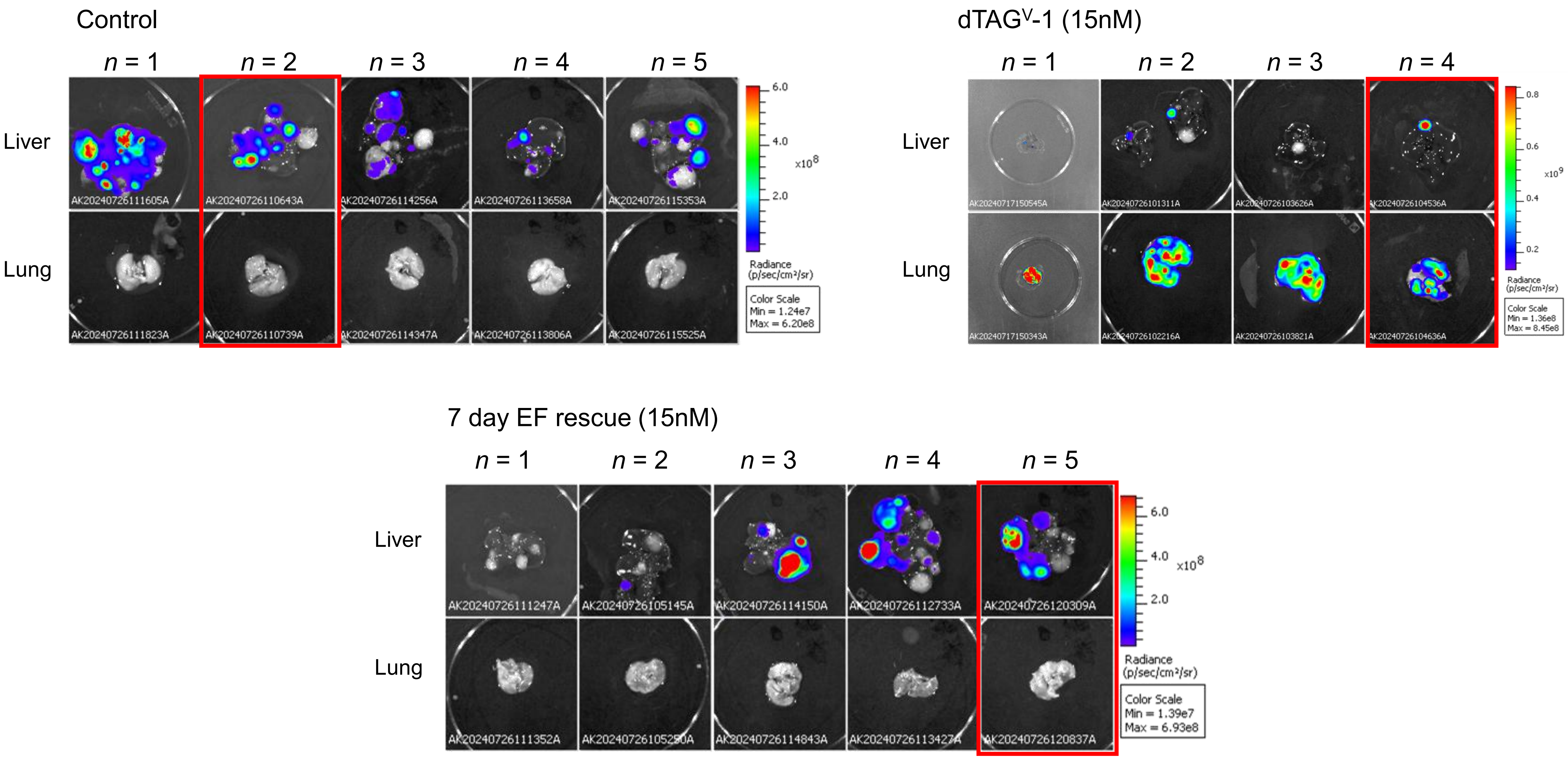

Supplement: Supplementary file 17 — Source data Fig. 6 [file IVIS images.pdf › 6E/IVIS images.pdf]
